# Kinship practices at the early bronze age site of Leubingen in Central Germany

**DOI:** 10.1038/s41598-024-54462-6

**Published:** 2024-02-16

**Authors:** Sandra Penske, Mario Küßner, Adam B. Rohrlach, Corina Knipper, Jan Nováček, Ainash Childebayeva, Johannes Krause, Wolfgang Haak

**Affiliations:** 1 Department of Archaeogenetics, Max Planck Institute for Evolutionary Anthropology, 04103 Leipzig, Germany; 2 Thuringian State Office for Heritage Management and Archaeology, 99423 Weimar, Germany; 3 School of Computer and Mathematical Sciences, University of Adelaide, Adelaide, 5005 Australia; 4 Curt-Engelhorn-Zentrum Archäometrie gGmbH, 68159 Mannheim, Germany; 5 Institute of Anatomy and Cell Biology, University Medical Centre, Georg-August University, 37075 Göttingen, Germany; 6 Department of Anthropology, University of Kansas, Lawrence, KS 66045 USA

**Keywords:** Archaeology, Population genetics

## Abstract

With the beginning of the Early Bronze Age in Central Europe ~ 2200 BC, a regional and supra-regional hierarchical social organization emerged with few individuals in positions of power (chiefs), set apart by rich graves with extensive burial constructions. However, the social organization and stratification within the majority of people, who represent the non-elite, remain unclear. Here, we present genome-wide data of 46 individuals from the Early Bronze Age burial ground of Leubingen in today’s Germany, integrating archaeological, genetic and strontium isotope data to gain new insights into Early Bronze Age societies. We were able to reconstruct five pedigrees which constitute the members of close biological kinship groups (parents and their offspring), and also identify individuals who are not related to individuals buried at the site. Based on combined lines of evidence, we observe that the kinship structure of the burial community was predominantly patrilineal/virilocal involving female exogamy. Further, we detect a difference in the amount of grave goods among the individuals buried at Leubingen based on genetic sex, age at death and locality but see no difference in the types of grave goods.

## Introduction

**Figure 1:**
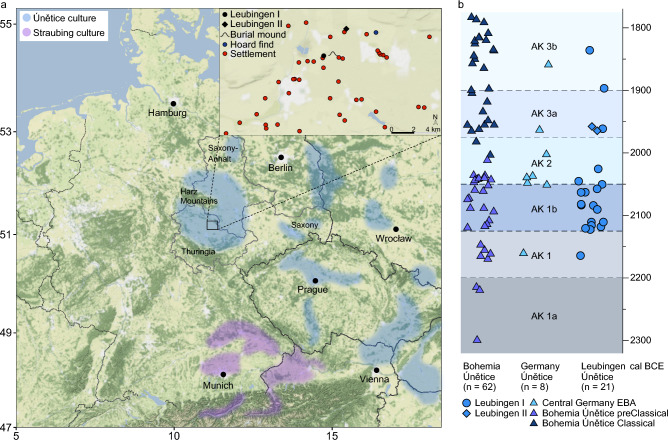
Geographic location of Leubingen and chronology of the burial grounds. (**a**) Leubingen is located in the Thuringian basin in Central Germany within the northern distribution area of the Únětice culture in Central Europe. Contemporaneous to the Únětice culture is the Straubing culture in southern Germany. The map tile set is © Stamen Design, under a Creative Commons Attribution (CC BY 3.0) license and was plotted with ggplot2^41^. (**b**) Chronology of the Circum-Harz group of the Únětice culture and mean calibrated ^14^C dates from published (Bohemia, Central Germany) and newly reported individuals (Leubingen). Here, only individuals with genome-wide data and a ^14^C date are shown.

The Early Bronze Age (EBA) Únětice culture in central Germany (2200-1550 BCE^1^) is characterized by significant changes in economy and social stratification with the emergence of chiefdoms when compared to the preceding Neolithic period^2,3^. The geographic distribution of the Únětice culture extended from Lower Austria and Slovakia in the Southeast, to the eastern edge of the Harz Mountains in Central Germany (Fig. 1a). Within Germany, the Únětice culture was distributed in today’s states of Saxony, Saxony-Anhalt, Thuringia and the southeasternmost part of Lower Saxony. In the region around the Harz mountains, archaeological finds attributed to the Únětice culture are combined into the Circum-Harz group. The Circum-Harz region is characterized by highly fertile loess soils, mostly black soils (Chernozem), and a low mean amount of precipitation (> 500 l/m^2^/year), which provides ideal conditions for agriculture^4^. Here, the discovery of richly equipped ‘princely’ burial mounds such as Helmsdorf^5^, Bornhöck^6^ and Leubingen^2,7^, compared to the poorly furnished graves in burial grounds lead to the assumption of a social stratification within the Circum-Harz group^2,3,8,9^. In addition, geographically close hoard or depot finds of copper and, more recently, bronze artifacts^3,10–13^, as well as a surplus of agricultural products, mark the region as a center of political, military and religious power, with few individuals in a powerful position related with political authority and legitimation^4,14^. This is also reflected by the quality and quantity of grave goods from individuals buried in the tumuli (mounds) compared to regular burials of the Central German Únětice culture. Golden artifacts were found in the princely burials of Leubingen, Helmsdorf and Bornhöck, with a total amount of gold of 256 g for Leubingen and an estimated total amount of ~ 1232 g for the Bornhöck^15^. Social stratification has been already assumed for the preceding Final Neolithic period (2800–2300/2200 BC). Rare finds of outstanding individual burials such as the Bell Beaker-associated archer from Apfelstädt^16^ support this theory, however, such clear signs of a social stratification and inequality, as observed for the EBA Únětice in Central Germany, cannot be supported for this time period.

By contrast, most regular burials of the (early) Circum-Harz group (2200–2000 BC) were only sparsely equipped, sometimes lacking any grave goods. However, this is only specific to this region, whereas burials in the southern distribution range of the Únětice culture show an abundance of high-quality grave goods, e.g., the burial site of Mikulovice, which is located along the Amber Road in Bohemia, Czech Republic^17^. Yet, based on recent growing archaeological evidence for Únětice settlement activity in Central Europe, it has become possible to investigate the differences between Únětice groups and neighboring EBA cultures systematically^4,18–20^. The resulting increase in data has also led to a better understanding of the settlement history, and with that, of the possible origin of the Únětice culture in Central Germany. Its emergence has been long debated, especially in connection with the preceding local Bell Beaker phenomenon (2500–2100 BCE) and Corded Ware complex (2800–2200 BCE) (BB and CW, respectively)^1^. Evidence for settlement activity shows a co-existence of both BB and CW towards the end of the Neolithic for around 300 years by sharing the same settlement area, but not the same settlements of the highly fertile Circum-Harz region^3^. Moreover, both maintained their own distinct burial rites and differences in their material cultures, which ended with the emergence of the Únětice^3,21,22^. These findings have led to the assumption of a succession of the Corded Ware, Bell Beaker and Únětice phases/periods, with the Corded Ware being slowly replaced by the more ‘innovative’ Bell Beaker phenomenon, which was then itself replaced by the Únětice culture. Recent archaeogenetic studies showed that CW and BB-associated individuals did not replace each other, but slowly admixed/amalgamated, resulting in the genetic profile of the EBA Únětice in Central Europe^23^. Comparative investigations of the grave goods of the princely burial mounds of central Germany also support that the Únětice material culture incorporated influences from both preceding groups^3^.

The increase in archaeological context data for the Central European EBA has also opened up the possibilities for new fine scale analyses. Moreover, the study of genetic relatedness and kinship between individuals buried at the same or different sites based on genome-wide data from entire burial grounds improves our understanding of life histories and social organization during the EBA substantially. This approach has yielded the potential to co-analyze the results of genetic-relatedness estimates with indicators of social stratification, such as wealth inequality, on a local scale^24–29^. However, the few integrated studies available thus far do not allow for generalized statements about kinship structures and social organization during the European EBA. Indeed, the EBA sites studied, such as Mokrin (Serbia), La Almoloya (Spain), Schiepzig and the Lech Valley (Germany) differ in their layout and respective burial practices, e.g., burials within the settlements^28^, funerary places directly associated with farmsteads^24,29,30^, smaller necropoles^27^ or closed burial grounds possibly associated with a settlement.

Leubingen is geographically located within the Thuringian basin in the modern-day state of Thuringia, Germany, and hence within the southern distribution range of the Circum-Harz group (Fig. 1a). The site was located near important exchange routes between east, west, north and south. The highly productive agricultural region in the eastern part of the Thuringian Basin was densely settled with hamlets and farmsteads, often only a few hundred meters or a few kilometers apart, as it was typical for the northern settlement area of the Únětice culture^31^. The burial ground and graves described in this study are in close proximity to the princely tumulus of Leubingen, which was dated to the beginning of the classical phase of the Únětice culture (1942 ± 10 BC, tree-ring date)^7,32,33^ (Fig. 1a, Supplementary Fig. S1) and is the oldest known princely burial from the Únětice culture^3^. The male individual buried in the mound must have been in a position of administrative, military and/or religious power. His high extraordinary social status is concluded from being buried in a wooden central chamber with numerous exceptional grave goods, including a vessel, a massive stone axe, a cushion stone, three chisels, two axes, three daggers, a halberd made out of copper/bronze, a bracelet, two pins, two rings and a small spiral made out of gold^9,34,35^. However, further bioarchaeological studies on the male individual from the burial mound are no longer possible since the human remains have been lost over the decades since the first excavation in 1877.

Approximately 800 m southwest of the burial mound, a burial place of at least one settlement associated with the Únětice culture^11^ was excavated between 2009 and 2010, which dates around 100–200 years earlier than the mound (Leubingen I, Fig. 1a, b, Supplementary Table A, Supplementary Fig. S1, S2). Moreover, about 2.5 km northeast of the mound, burials were discovered which belonged to a single farmstead and were partially contemporaneous to the tumulus (Leubingen II, classical Únětice phase) (Fig. 1b, Supplementary Fig. S1, S2).

With the exception of the burial mound, grave goods at both Leubingen burial grounds were sparse and low in quality compared to other regions^17,24^. This is likely due to a manifestation of a custom in which grave goods appear as *pars pro toto* for the entire furnishing of the living, or because the clothing/garment of the local group was less elaborated. However, the variation within the grave goods allows conclusions about the status of the buried individuals within the burial community^36,37^, which needs to be taken into account when making supra-regional comparisons. The individuals of the burial grounds of Leubingen likely represent part of a local population of the northern distribution range of the Únětice culture, which provided the base for the EBA economic and social system based on subsistence farming with a surplus alongside exchange and craft production.

By examining the individuals of the burial ground, including their grave goods, we aim to answer questions about biological relationships, social kinship structures, union and residence rules, economic units as well as the connections between these units. In the context of the political structure, which presumably represents a chiefdom^12^, we aim to find evidence for or against social stratification, as well as ‘horizontal’ relationships between individuals and genetic lineages. Through the co-analysis and contextualization of genome-wide, radiogenic strontium isotope, osteological/anthropological and archaeological data, this study aims to contribute to the understanding of the kinship structure and social organization of EBA communities of the (northern) Únětice culture.

## Results

### Genomic and isotopic data

Leubingen I consists of a core area with 33 graves, which held the remains of 46 individuals. The surrounding excavation area of approximately 22.5 ha uncovered the remains of 30 additional individuals (Supplementary Fig. S1). Leubingen II consists of four graves with the remains of five individuals (Fig. 1a,b, Supplementary Table A). In total, 46 features with 59 individuals associated with the Únětice culture were archaeologically documented (Supplementary Table B). In addition, 22 more features with 23 individuals were identified within Leubingen. However, their attribution to the Únětice culture is uncertain and they were therefore not included in this study (Supplementary Table B).

Applying a set of established authentication and data quality criteria (characteristic DNA damage, negligible levels of contamination, unambiguous genetic sexing), we report new high-quality genome-wide data (1240 k SNP capture data^38^) for initially 47 out of 52 sampled individuals associated with the Únětice culture with a mean coverage between 0.03X and 1.8X (Supplementary Table A, Material and Methods). Two samples (2029-1 and former 2029-2) were found to come from the same individual (Supplementary Information) and were therefore merged into 2029-1, resulting in 46 individuals for downstream analyses (Supplementary Table A, Material and Methods). For 21 of these individuals/samples we also obtained new ^14^C data (Fig. 1b, Supplementary Table A, Supplementary Fig. S2). In addition, we sampled 40 teeth from 40 individuals as well as bones and teeth from 8 supposedly autochthonous animals such as cattle, pig and sheep/goat from the burial ground for strontium isotope analysis (^87^Sr/^86^Sr) (Supplementary Table C) to determine human residential origins and mobility patterns. The majority of individuals (n = 31) indicated a local isotopic signal of between 0.7085 and 0.7095, which is a typical isotope composition of strontium being biologically available from loess and calcareous soils in Central Germany^39^ (Fig. 3c).

The individuals from Leubingen were grouped chronologically and spatially into the main burial site of Leubingen I (early and middle Únětice phase AK 1b^40^, AK = Aunjetitzer Kultur) and the later Leubingen II (classical Únětice phase) 2.5 km to the northeast of Leubingen I (Fig. 1a,b, Supplementary Table B). Three individuals from Leubingen I also date to the classical Únětice period. Their location with respect to the main burial place indicates a later and sparser use of the site. Published Central German individuals with genome-wide data date mainly to the earlier phase AK 2 and published Bohemian Únětice-associated individuals cover the entire range of the Únětice period (Fig. 1b, after Schwarz 2019; 2021^40^).

### Genetic relationships and mobility in Early Bronze Age Leubingen

**Figure 2:**
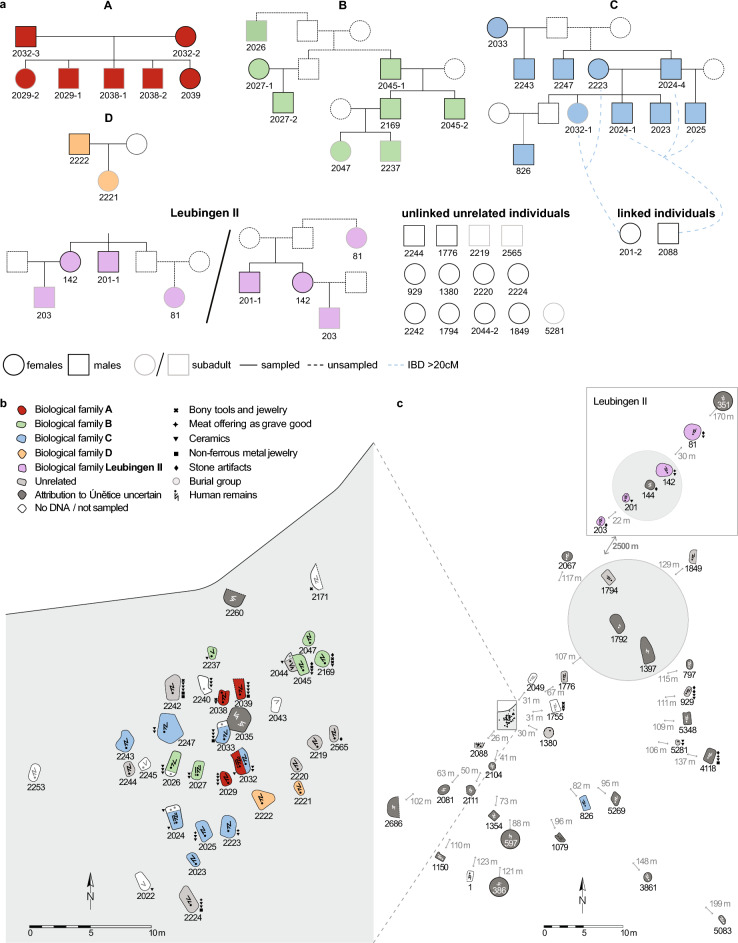
Reconstructed pedigrees and spatial layout of the burial ground of Leubingen I and II. (**a**) Four pedigrees were reconstructed for Leubingen I with up to four generations and one for Leubingen II with either two or three generations. Genetically female and male individuals who are not present at the burial ground, but were inferred to have existed, are indicated by a dashed outline. Present adult and subadult individuals are shown with a black and grey outline, respectively. Individual labels consist of the feature number. (**b**) Zoom-in into the main burial ground of Leubingen I where the majority of the genetically related individuals of pedigrees A-D were buried, as well as graves in their vicinity (**c**) Layout of the wider burial field, with the burial ground of Leubingen II approx. 2.5 km to the northeast. The burials are associated with a single farmstead. Note that the grey circles around feature 386, 597 and 351 do not indicate the burial outline but mark the uncertainty about the attribution to the Únětice culture. Find an overview over the excavation area of Leubingen in Supplementary Figure S1 online.

**Figure 3:**
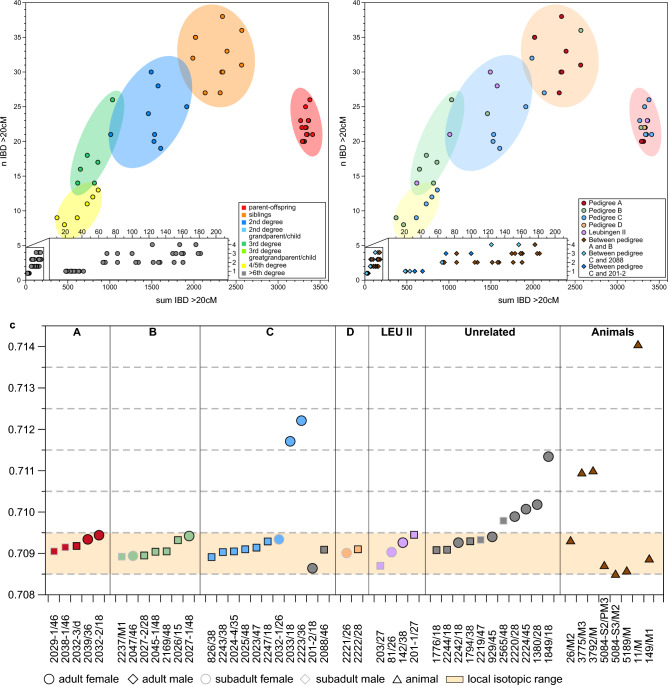
Identity-by-descent (IBD) results and strontium isotope ratios (^87^Sr/^86^Sr) of individuals from Leubingen I and II. Plotting the sum versus the number of shared chunks of IBD in window sizes of > 20 cM resolves degrees of biological relatedness up to the fourth - sixth degree (**a**) between pairs of individuals and (**b**) between pairs of individuals within and between pedigrees. (**c**) Strontium isotope ratios of 40 individuals from Leubingen. Shown are the feature numbers and the tooth that has been sampled (Supplementary Table C). The yellow bar represents the local isotopic range of the area. Two individuals from pedigree C and five unrelated individuals show a non-local isotopic profile. (Color and pedigree codes correspond to Fig. 2).

Using BREADR^42^ to estimate genetic relatedness revealed 41 first-degree relationships and 24 second-degree relationships among the 46 individuals (Supplementary Table D). From these close genetic relationships, we reconstructed four pedigrees for Leubingen I (A–D) and one pedigree with two possible topologies for Leubingen II (Fig. 2a). Furthermore, the results of identity-by-descent (IBD) analysis (ancIBD)^43,44^ of 35 individuals with ≥ 400 k SNPs (Supplementary Table A) confirmed the reconstructed parent-offspring, sibling, avuncular, grandparent-grandchild and third - fifth-degree relationships, and therefore the robustness of the reconstructed pedigrees (Fig. 3a, Supplementary Table E). Lastly, IBD sharing was used to test for higher degree genetic relationships between individuals with > 400 k SNPs covered, with the potential to detect genetic links between pedigrees and with individuals considered ‘unrelated’ based on BREADR (Fig. 3b).

#### Pedigree A

Pedigree A consists of two generations and is also the pedigree with the highest number of children per union observed in Leubingen (Fig. 2a). Out of the five children, only the female individual 2039 reached adulthood and was buried in a single grave. All graves were close to each other and in north–south orientation (Fig. 2b). The other four children (three boys and one girl) died during the infans I or II stage, with the oldest child, 2029–1, reaching a maximum of 10 years of age (Supplementary Table A). Close biological relatedness is also consistent with the Sr isotopic data with all individuals from this pedigree yielding local ^87^Sr/^86^Sr ratios (Fig. 3c), meaning all individuals grew up within the economic region of the settlement community of Leubingen. Of the four subadult offspring 2038-1 and 2038-2 were buried together in one grave, as well as 2029-1 together with 2029-2. Here, the parents, 2032-2 and 2032-3, were also the only individuals from a union that were laid to rest together, albeit with a time difference since the father, 2032-3 was found in Planum 5 and the mother, 2032-2, was found in Planum 3 (Supplementary Information). In Planum 2, 2032-1, the daughter of 2223 (F) and 2024-4 (M) from pedigree C, was buried above 2032-2 from pedigree A. 2032-3 and 2032-2 were not buried with one of their infant children and the infant child 2032-1 was not buried together with any of her parents from pedigree C. This situation poses questions about the concepts of genetic relatedness and social kinship at the site. It is possible that 2032-3 and 2032-2, or maybe only 2032-2 (since her partner died before her) assumed the role of foster parents. Further, out of the individuals with more than 400 k SNPs, IBD analyses show that the female individual 2032-2 is the only individual of the two pedigrees A and B who has no genetic connections to any other individuals than her children (Supplementary Table A, E, Supplementary Fig. S3). Interestingly, all children of pedigree A are related to individuals from pedigree B in the sixth degree (or possibly closer), as shown by up to four shared IBD tracts of > 20 cM length (Fig. 3b, Supplementary Fig. S3).

#### Pedigree B

Pedigree B includes eight individuals across four generations, which are connected through the paternal line, such as the direct father/son/grandson line from individuals 2045-1 to 2169 and to 2237. By contrast, adult female individuals, present and inferred, are linked to the pedigree through their children only and have no parents or siblings buried at the site. Both children of the last generation of this pedigree died as children: between the ages of nine and ten (2047) and two and three (2237). The spatial orientation of the graves is reflected in a ‘left’ and a ‘right’ side of the pedigree with 2026, 2027-1 and 2027-2 (left side) buried closely together in the south-western part of the main burial ground, and 2045-1, 2045-2, 2047 and 2169 (right side) buried closely together in the north-eastern part. Individual 2237, the son of the last generation of this pedigree is buried in a single grave between the two groups (Fig. 3a,b). 2027-1 and 2027-2 are mother and son, and they were buried together in one grave where the son, 2027-2, likely died first as 2027-1 was buried on top of him. However, a simultaneous burial of both individuals cannot be excluded (Supplementary Information). 2045-1 and his adult son 2045-2 were buried together in a stone cist (Fig. 3a,b, Supplementary Information). Directly next to it we find a secondary burial with two individuals, 2044 and another individual from whom no sample was taken due to preservation. All individuals from pedigree B also show local ^87^Sr/^86^Sr ratios (Fig. 3c). In addition, the observed IBD sharing between pedigrees A and B suggests that both families either were residing in the same area or that they had returned to Leubingen.

#### Pedigree C

Pedigree C comprises ten individuals across four generations, also linked through the paternal line (Fig. 2a). Interestingly, we observe two half-sibling constellations, also related through the father’s side, in the second and the third generations. Individual 2243 is the half-brother of full siblings 2024-4 and 2247, and individual 2025 is the half-brother of 2023, 2024-1 and 2032-1. By contrast, female individuals 2033 from the first generation and 2223 from the second generation of the pedigree are linked to the pedigree through their children only and have neither parents nor siblings buried at the site, and thus can be considered exogenous. This is also supported by the ^87^Sr/^86^Sr ratios of their teeth, which are in both cases more radiogenic than those of the other individuals from this pedigree and fall outside of the local range (Fig. 3c). Both individuals were buried in the main burial ground, in single graves with grave goods (Fig. 2b, Supplementary Table B), with their children in close proximity (< 10 m) (Fig. 2b). The only other female individual (2032-1) in this pedigree died between 11 and 13 years of age and is also the only female ‘lineage’ individual that is linked to other individuals in the pedigree through parent-offspring, sibling, and avuncular relationships. Unlike most of the other members of pedigree C who were buried alone, 2032-1 was buried together in one grave with two individuals from pedigree A (Fig. 2b, Supplementary Information). Only two individuals of pedigree C, the father 2024-4 and his adult son 2024-1, were buried together in a common grave with two other individuals who were sampled but did not yield any DNA (Fig. 2b, Supplementary Information). The adult male individual 826 of the last generation is the only relative who was not buried in the main burial ground but ~ 82 m to the southeast of it. It is possible that he was buried at his place of residence at a time when the main burial ground was not in use anymore. A second possibility is that he left the community to start his own family but had no offspring at the time of his death or his descendants died later after moving to another place. Another less likely option is that he was returned after his death to be buried closer to his family. His ^87^Sr/^86^Sr ratio indicates a local upbringing (Fig. 3c). All male individuals from this pedigree had reached adulthood (defined as > 16 years old), but with the exception of 2024-4 no individual had any offspring (buried at the site), yet they are linked to other individuals in the same and other generations via parent-offspring, sibling and avuncular relationships.

Furthermore, through the analysis of shared IBD tracts we were also able to link 2088 and 201-2, two previously labeled ‘unrelated’ individuals, to pedigree C (Supplementary Fig. S4). The male individual 2088 is genetically linked to this pedigree via four tracts of > 20 cM in length to 2024-4, the only male individual with detectable offspring, to his son 2024–1, and with three tracts of > 20 cM to 2025, the half-brother of 2024–1 (Figs. 2a, 3b, Supplementary Table E). In addition, a female individual 201-2 from the chronologically younger burial ground Leubingen II also shares IBD tracts of > 20 cM in length with 2223 and 2032-1 indicating a fifth-degree relationship or higher (Fig. 3b, Supplementary Table E). Both individuals, 2088 and 201-2, have a local ^87^Sr/^86^Sr ratio (Fig. 3c). This observation indicates that both individuals stayed in the area, or, in the case of 201-2, that her family stayed around for another 100 to 200 years after the main burial ground was abandoned and she was later part of the small burial group of Leubingen II.

#### Pedigree D

Pedigree D is the smallest one in Leubingen and consists of a father (2222) and his daughter (2221) (Fig. 2a). The daughter died when she was between 4 and 5 years old. Both individuals yielded ^87^Sr/^86^Sr ratios within the local range (Fig. 3c) and were buried in single graves at the south-eastern end of the main burial ground, in close proximity to each other and with no grave goods (Fig. 2b, Supplementary Table B, Supplementary Information).

#### Leubingen II

Four individuals from Leubingen II were connected in a pedigree with two possible topologies. The first possibility is a pedigree spanning over two generations, in which 81 is the niece of the sibling pair 142 and 201-1, another possibility being that she is their aunt, resulting in a three-generation pedigree (Fig. 2a). With the results available to us and in combination with age at death we cannot determine which option is most likely, since both methods return the relationship between the sibling pair 142 and 201-1 and the third individual 81 as third degree without specification of the quality of the relationship (Fig. 3a, Supplementary Table D, E). Leubingen II is the only pedigree that is linked via the maternal line, in this case through 142. The ^87^Sr/^86^Sr of all four individuals from Leubingen II fall into the local range.

Another individual, 201-2, who is not related to individuals of the reconstructed pedigree Leubingen II, is genetically distantly related (≥ fifth degree) to pedigree C through 2223 and 2032-1 (Figs. 2a, 3b, Supplementary Table E).

### Insights into burial, kinship and residence practices

#### Sex ratios and preferential burial practices

We note a lack of eight female and six male individuals (Supplementary Fig. S4) in all reconstructed pedigrees except pedigree A and excluding hypothetical parents of generation 0. All missing individuals are part of a union and are a parent, four out of the six male individuals are also a brother to another individual in their pedigree. Only one inferred male individual, the father to 203 in Leubingen II is not related to anyone else in his pedigree. By contrast, all missing female individuals are only mothers to their children and are neither daughters nor sisters to other individuals in their pedigrees. Overall, the sex ratio observed at the site is unbalanced. On the basis of the natural male/female sex ratio at birth of 1.05:1^45^, we would expect more female individuals in the pedigrees than we observe. Instead, we find 17 sons and 6 daughters (the first generation is excluded and only individuals that are present are counted) resulting in an observed subadult ratio of 2.83:1 (95% confidence interval of between 1.8:1 and 4.91:1). This bias suggests that daughters who reached adulthood might have left the community and were buried elsewhere.

The sex ratio across the site based on all sampled, inferred and unrelated individuals, including 31 male and 29 female individuals, is balanced (*p* = 0.7962). However, the excess of adult sons without offspring (N = 8) versus adult daughters without offspring (N = 1) buried at the site is significant (*p* = 0.0196). This imbalanced ratio is partially corrected by the unrelated individuals of which the majority are adult females (N = 9). Taken together, this supports the practice of female exogamy. Within the pedigrees we found 19 female and 26 male individuals in total (including inferred individuals) of which 11 female and 20 male individuals received a burial at the site (which was discovered). In total, we find 13 unions, in which mothers (N = 8) and fathers (N = 6) were missing at a similar ratio. We note though that it is only six males that are missing and not seven because we find two half-sibling constellations in pedigree C of which one includes a missing father (Fig. 2a). In total, out of 13 mothers and 11 fathers, five of each are present at the burial ground (Supplementary Fig. S4). None of these ratios are significantly imbalanced. It is of importance that there is a difference between missing male and female individuals and their relationships, meaning that all inferred missing female individuals are only related to their offspring and are not related to other individuals. By contrast, four out of the six missing male individuals are brothers in addition to being fathers and are therefore already linked/nested within the pedigree. The only male individual that is not related to anyone except his offspring is the biological father of 203 in the pedigree of Leubingen II, the only pedigree linked through the female line (142).

The absence of mothers and adult daughters, as well as the non-local isotopic values in two of the mothers and the unrelated female individuals and the fact that all missing mothers are not nested within the pedigrees unlike the missing fathers (Figs. 2a, 3c, Supplementary Fig. S4) indicate higher levels of female mobility, especially for adult women.

In all five pedigrees we find only close biological kinship units buried together, meaning the majority of the successfully studied individuals are parent–child units who were buried over several generations. The pedigrees seem to only reflect the immediate genetic relations which suggests that households likely operated at the level of smaller family units while the spatial layout of the burials mirrors also extended genetic and social relations of the community (Fig. 2b).

#### Patterns of lineality, locality and mobility

To formally test the relatedness of individuals and therefore possible signs of matri- or patrilocality/lineality we performed an analysis of variance of the pairwise mismatch rates (PMR) estimated using BREADR using the age and genetic sex of a pair of individuals as predictors (Supplementary Information). When looking at the PMRs between different genetic sex pairings (XY/XY, XY/XX, XX/XX), we find no significant difference (*p* = 0.999) between PMRs calculated on XX/XX and XY/XX pairs, on average, but a significantly lower PMR for XY/XY pairs when compared to XX/XX and XX/XY (*p* < 2.2 × 10^–16^) (Supplementary Fig. S5). This indicates that male individuals are on average more closely related to each other than they are to females, or than female individuals are to each other. We also find that the age (adult/adult, adult/subadult and adult/adult) was also a significant predictor. There is no significant difference in the average PMR values for subadult/subadult and adult/subadult comparisons over all three genetic sex pairings, however, both age pairings were found to have significantly lower PMR values when compared to adult/adult pairings. This is consistent with the number of sibling and parent–offspring relationships that we find at Leubingen, with the pedigrees representing close genetic kinship units in each generation without extended family and with the majority of unrelated individuals being adults. However, we note that the effect size for the age group was an order of magnitude lower than for sex. To further explore this finding, we performed Wilcoxon-rank sum tests on the mean relatedness of each individual to all other individuals from the burial ground. We expect the mean PMRs to be lower for individuals who are more closely related to other individuals from the site, compared to the unrelated individuals. Here, we find no significant difference in the mean relatedness (*p* = 0.06727) for all individuals combined (Supplementary Fig. S5). However, when partitioned into adult and subadult groups, we find that the adult male individuals were more related to other individuals when compared to the females (*p* = 0.01404) (Supplementary Fig. S5). Conversely, we find no significant difference between male and female subadults (*p* = 0.7551), suggesting that younger children (specifically female) had not left the community before a certain age (around 16 years of age) (Supplementary Fig. S5).

#### Integrating evidence from unrelated individuals

In addition to the 31 closely-related individuals in the above pedigrees, and the two individuals who are connected to pedigree C via shared IBD, we count 13 individuals who are not related to anyone from the burial site, of which nine are females and four are males, respectively. Out of the four males, two are early adults (older than 16 years old) and two are subadults. By contrast, eight out of the nine unrelated female individuals are adult women. Four of the females show non-local ^87^Sr/^86^Sr ratios (Fig. 3c). Interestingly, the unlinked, unrelated subadult male 2565 also shows a non-local strontium isotopic value. Seven of the 13 unrelated individuals have regular burials in the main burial ground similar to the related individuals, while six are buried farther away from the main graveyard (Fig. 2b,c). The number of adult, unlinked, unrelated females (n = 8) roughly equals the number of adult male pedigree individuals with no offspring (n = 6) (Fig. 2a). It is possible that they represent unions from which no offspring were found, which would then link these female individuals to the pedigrees. Therefore, while a genetic connection between the individuals cannot be explicitly defined, a social bond cannot be ruled out, in particular, for the individuals buried in close spatial proximity to pedigree individuals (Fig. 2b).

Two unrelated adult females (2224 and 2242) were buried with very rich grave goods in comparison to the majority of individuals (Fig. 2b, Supplementary Table B), suggesting prominent roles or positions in life. Another special case is 5281, a newborn female individual with a *pithos* burial who is not related to anyone in Leubingen. She is also not part of the main burial ground but was buried around 100 m to the east of the main burial. Here, the most likely explanation is that, due to poor preservation, we were not able to retrieve any genetic material linking her to her parents who may have been buried closer to her. It could also be that her parents left Leubingen after her death.

We note that six of the unrelated individuals have local ^87^Sr/^86^Sr ratios. This indicates that the local strontium isotopic range extends further than the economic region of the settlement community of Leubingen. Hence, it is possible that individuals who were growing up in the surrounding areas cannot be identified as non-local based on their ^87^Sr/^86^Sr ratios but were also coming from areas further away from the settlement area of Leubingen.

In a comparative study from the Lech Valley in southern Germany^24^, the reconstructed pedigrees were linked to single farmsteads, a principle which only applies to the pedigree of Leubingen II. Instead, the main burial ground at Leubingen spatially connects the majority of individuals from pedigrees A-D, and a number of unlinked, unrelated individuals who likely lived at the settlement(s) of Leubingen^11^ around the same time (Fig. 1b, Supplementary Table A). There are several possible explanations of the spatial organization of the burial ground of Leubingen I: (I) it was a collectively managed funerary place for a small settlement (hamlet) consisting of three or four farmsteads, each of which was managed by extended families^11^, (II) the people of several individual farmsteads which did not function as a settlement buried their dead in a common "central" burial ground, or (III) there was no direct reflection of the actual cohabiting and farming communities in the burial place, but there were other rules underlying the choice of the burial place for the deceased. Combined archaeological and genetic evidence^29^ supports possibilities (I) and (II) for Thuringia^12,31^ and the whole Circum-Harz group^4^.

#### Examining the distribution of grave goods in the light of the inferred kinship structure

**Figure 4:**
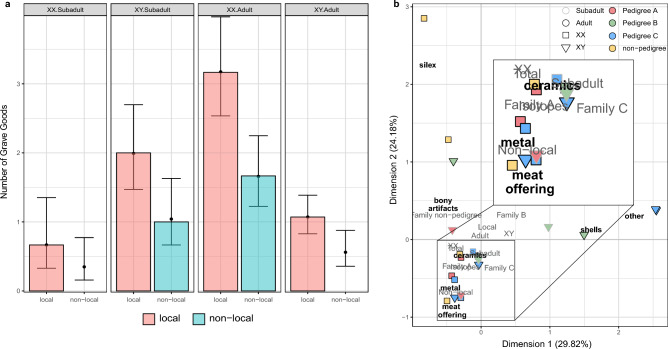
Grave good analyses of Leubingen I. (**a**) Results of the Poisson regression model with the parameters age at death, genetic sex and locality based on Sr ratios. The height of the bars is the observed mean count of the number of grave goods, the points are the predicted average, with ± one standard error. A missing bar means no individuals with the mix of variables were present. (**b**) A correspondence analysis of the counts of different grave goods types per individual. Individuals with no grave goods were excluded (Supplementary Information). Dimension 1 differentiates between individuals buried with shells/other and individuals without these grave goods. Dimension 2 differentiates between individuals buried with silex/bone artifacts and food offerings. Plots were created with ggplot2^41^.

Leubingen I and II comprise burials with ceramics (n = 26), food offerings (n = 5), metal objects/green coloration (n = 4/2) and weapon parts (n = 2) as well as graves without grave goods were also represented (n = 36) (Supplementary Table B). Among the 34 graves in the main burial ground of Leubingen I, there were 21 burials with ceramics (up to four vessels), four with food offerings, three to five with metal objects and one with an arrowhead. We were interested in investigating if the sex, age at death, or whether an individual was “local”, affected the type or number of burial goods an individual received. Using Poisson regression, we find differences in the total number of grave goods between related and unrelated, local and non-local, and adult and subadult individuals in Leubingen I (Fig. 4a). Adult isotopically local female individuals were equipped with the highest number of burial goods compared to all other present categories (Supplementary Table B, Supplementary Information). Subadult local male individuals had the second highest total number of grave goods. Non-local individuals received fewer grave goods than their local counterparts. However, adult non-local females and non-local subadult males have a higher total number of grave goods than local subadult females. When investigating a possible connection between these same variables and the number of different types of grave goods that each individual received, we find no significant single predictor in the model, meaning that age at death, genetic sex, locality and relationship status do not determine how many different types of grave goods an individual received (*p* = 0.4588).

One reason for the higher number of grave goods in adult females, compared to adult male individuals, could be the presence of copper/bronze artifacts in only female burials (Supplementary Table B), which represent part of the adult female clothing set. However, subadult local female individuals were buried without this type of artifact. The appearance of copper artifacts in Leubingen is earlier than described in previous studies^1,40^, where they are only found in the region during the phase 2b. This suggests that individuals buried at Leubingen were well-off for this time period and region, albeit not as rich as in other regions of the Únětice culture^46^.

At Leubingen I, the total number of grave goods an individual received after their death depended on their age at death, their genetic sex, and whether they were local or non-local. However, the number of different types of grave goods was independent of all of these variables (Supplementary Table S1) and hence it could be speculated that the choice of what an individual received as grave goods was dependent on their position within the family or community without there being a clear social stratification or social inequality. The choice of burial items appears to be a more selective process on an individual level rather than on a societal level.

We then used correspondence analysis (CA) to explore the relationships between different combinations of grave good types, and the individuals who had them. We find that individuals buried with shells/other grave goods (Supplementary Table B) did not receive any other type of grave goods (except ceramics), and vice versa, and that a similar negative correlation existed between silex/bone artifacts and food offerings (Fig. 4b, Supplementary Information). Ceramics were the most common grave good in combination with others, and thus the least informative grave good type, hence the position in the CA plot near the origin (Fig. 4b). With respect to demographic variables (age at death, genetic sex, local/non-local, pedigree) we find no correlation with the different types of grave goods (Supplementary Table S1). Of note, for the CA we excluded individuals with a total count of zero grave goods, such as those of pedigree D and seven unrelated individuals.

When compared to the results from the Lech Valley in southern Germany, where almost all unrelated and non-local individuals are well equipped with grave goods and only two had no grave goods^24^, we find this to be the opposite in Leubingen. Here, only two well-furnished graves of unrelated and non-local female individuals were found whereas the remaining ones have few to no grave goods (Supplementary Table B,C). For the Lech Valley it was speculated that individuals with a different social status lived in the same households. Here, it is not clear to determine whether the unrelated and/or non-local individuals who are not part of a pedigree were living with the families in extended family units. It is also possible that these individuals, especially the adult women who were buried within the main burial ground, were part of a unit with local adult males but without offspring at the time of their death (or adult offspring that had left the community). All would be consistent with the practice of female exogamy, in which women were leaving Leubingen to form family unions in a different village or area, but also coming in from other areas to form local unions.

#### Comparative studies

Kinship structures involving patrilineality and female exogamy at Leubingen are suggested by I) pedigrees B and C which are linked via the paternal line, II) by a higher mean relatedness between adult male individuals compared to female individuals (Supplementary Fig. S4a, b), III) by the position of the missing adult female individuals in the pedigrees (Supplementary Fig. S4), and IV) higher isotopic values of some female individuals compared to male individuals (Fig. 3c). However, we caution that the interpretation of local Sr isotope ratios can be difficult given the highly similar wider isotopic landscape around the settlement hub of the eastern Thuringian Basin (Fig. 1a)^39^. In fact, individuals who fall within the Sr isotope variation of the site, could still be geographically non-local but from an area with a similar isotopic profile. It can be assumed that close neighboring communities were considered non-local, ‘foreign’ or different from oneself and therefore mobility of female individuals occurred between close villages that were different enough but with a similar isotopic profile^39^.

Similar patterns in support of female exogamy were also reported in the Lech Valley study from southern Germany and EBA sites in Iberia, such as La Almoloya^24,28^. Our findings from Leubingen seem to confirm that this practice was not an exception. At other EBA sites, e.g., Mokrin, Serbia^27^, female exogamy was practiced during the EBA, but the reported higher diversity of Y-haplogroups suggest that the effects of patrilocality were not as pronounced as in the Lech Valley and in Leubingen. Compared to the Lech Valley and Mokrin, parent–offspring burials were more common at Leubingen, with two father-son and one mother-son burials. By contrast, adult double burials were more prevalent in La Almoloya, but parent–offspring burials were equally common^28^.

When comparing to broader chronological references, patterns in support of patrilocality and female exogamy were also reported in Late Neolithic sites associated with the BB phenomenon^25,47^, the Globular Amphora culture^48^ and also Middle and Early Neolithic sites from France and England, respectively^49–51^. While it is tempting to infer broader trends from the observed similarities across Neolithic and Bronze Age societies from Europe, and even though the Únětice material culture is largely influenced by the locally preceding BB and CW^3,40^ horizons, more directly comparable, integrated studies are needed to explore whether these cultural influences also affected social practices and kinship structures.

## Concluding remarks

The burial ground of Leubingen represents a farming population of the EBA Únětice culture in Central Germany. Both funerary places, Leubingen I and II, contribute to a further understanding of EBA societies and their social organization. It could be shown in the past that the cultural emergence of the Únětice culture in Central Germany was the result of the CW dissolving into the BB culture. Our new data (Supplementary Information) shows that individuals associated with the Únětice culture in Central Germany and Bohemia carried a high amount of CW-related ancestry, and therefore form a distinctive cluster with little overlap in PCA space with EBA individuals from southern Germany. This result is consistent with previous genetic studies^13^ and with the archaeological evidence that the Únětice culture combines elements of the material culture of both the BB and the CW^3^.

Kinship structures on the basis of patrilineality and virilocality involving female exogamy have been reported from various Neolithic and Bronze Age sites across Europe and were likely adapted by many Bronze Age societies and cultures including the Únětice culture in Central Europe. Even though the amount and quality of grave goods in the Circum-Harz group is lower than in other regions of the Únětice distribution area, burials in Leubingen were well equipped and also contained copper/bronze grave goods that were more commonly found only around 100 years later. The observed difference in the amount of grave goods between local and non-local individuals but also between all groups and female children indicates an internal social differentiation which can be found in other EBA sites in Europe but with local variation.

The practice of female exogamy and patrilineality including a higher mobility of female individuals is consistent with previous studies and is supported by isotopic as well archaeogenetic results. Furthermore, Leubingen provides insights into non-genetic kinship relations based on burial practices and grave good distributions. With the genome-wide data from Leubingen we contribute to the amount of Únětice period individuals available from Central Germany. These results add further evidence to principal burial and societal practices in central European EBA societies which seem to be consistent with previous studies. Therefore, future studies focusing on comparable burial grounds from the region but also from other contemporaneous societies have the potential to investigate microscale variations but also contribute to a more nuanced understanding of social and economic organization.

## Methods

### Radiocarbon dating

All individuals used in this study were obtained from the Thuringian State Office for Heritage Management and Archaeology, Weimar, Germany with permission from the lead archaeologist M. Küßner. Of the 47 individuals reported in this study we obtained direct ^14^C dates for 22 individuals. Radiocarbon dating was carried out using accelerated mass spectrometry (AMS) at the Curt-Engelhorn-Zentrum Archäometrie gGmbH in Mannheim, Germany (Supplementary Table A). All samples were calibrated using the IntCal20 database and using OxCal v.4.3.2 as well as OxCal v4.4.2. All ^14^C dates in this study are consistent with the archaeological chronology based on stratigraphy and grave goods.

### Isotope analysis

Molars of individuals were primarily selected based on preservation and availability. The enamel of first molars (M1) forms in early childhood from around 0–3 years of age, the M2 (and premolar) from around 3–8 years and the M3 from 8 to 14 years. The incorporated Sr originates from foodstuffs and drink taken up during tooth formation. Hence, it reflects different episodes of childhood and adolescence depending on the sampled tooth. We preferentially selected M3s and M2s to ensure consistency in the ontogenetic stage reflected and to avoid an influence of breastfeeding on the isotopic composition of oxygen to be potentially determined for the same samples. If M3s were absent due to the young age of an individual or preservation, we sampled first molars od deciduous teeth instead. Data interpretation refers to infancy and adolescence in general, without temporal differentiation that would have required two or more samples per individual.

Sample preparation and analyses of strontium isotope compositions of tooth enamel of 39 individuals (Supplementary Table C) followed previously described steps^52–54^. Enamel fragments were cut from the crowns using a diamond-coated cutting disc attached to a dental drill. All surfaces and remaining dentin were removed using diamond-coated milling bits, and the samples powdered in an agate mortar. For Sr isotope analysis, 11–12 mg of sample material were pre-treated to remove diagenetic carbonates. In successive steps, the powder was placed in an ultrasonic bath for 10 min each with 1.8 ml of supra pure H_2_O, 1.8 ml of 0.1 M acetic acid buffered with lithium acetate (pH ca. 4.5) and three times with 1.8 ml of H_2_O. Samples were afterwards dried overnight (50 °C) and ashed to remove remaining organic components (3 h at 850 °C). All subsequent steps were carried out under clean lab conditions. The samples were dissolved in nitric acid (3 N HNO_3_) and the strontium separated using Sr-Spec ion exchange resin. Strontium concentrations were determined using an optical emission spectrometry with inductively-coupled plasma ionization (ICP-OES iCAP 7200), the solutions diluted, and the isotope ratios determined using a High Resolution-Multi Collector-Inductively Coupled Plasma-Mass Spectrometer (HR-MC-ICP-MS; Neptune). The raw data were corrected according to the exponential mass fractionation law to ^88^Sr/^86^Sr = 8.375209. Blank values were less than 10 pg Sr during the clean lab procedure, including digestion, Sr separation and measurement.

### Ancient DNA laboratory procedures

In total we processed 47 petrous bones and 5 teeth from 52 individuals from Leubingen associated with the EBA in dedicated clean room facilities of the Max Planck Institute for Evolutionary Anthropology in Leipzig, Germany. Petrous bones were sampled with a minimally invasive method^55^ and for teeth the crown was separated from the root and the inner pulp chamber was drilled out^56^. DNA was extracted from all samples following a modified protocol after Dabney et al*.*^57,58^. DNA double-stranded libraries were built for all samples using a partial uracil-DNA-glycosylase (UDG-half) treatment^59^. All libraries were double-indexed with a unique pair of indices^60^.

All indexed libraries were screened via shotgun sequencing of 5 million reads on an Illumina HiSeq 4000 or NextSeq500 sequencing platform using a single end (1 × 75-base pair (bp) reads) kit, followed by using EAGER v. 1.92.56^61^ to assess the human DNA content and DNA damage profiles (initial quality criteria). All libraries reached the threshold of > 2% endogenous DNA and were enriched for ~ 1.24 million single-nucleotide polymorphisms (SNPs) and sequenced for 20 million reads in one round of targeted in-solution capture (“1240 k SNP capture”^38^). Enriched libraries were sequenced on HiSeq4000 and NextSeq500 Illumina platforms using a single-read (SR 75) kit. This resulted in a mean coverage of 0.9x (Supplementary Table A). Mitochondrial capture (“MT capture”^62,63^) was performed for individuals with less than 3000 reads that had mapped to the mitochondrial genome, resulting in a mean coverage of between 3 × and 307x (Supplementary Table A). For all male individuals we also performed an in-house capture assay for the Y-chromosome (“YMCA”^64^) which targets ~ 10.445 kB on the non-combining region of the Y chromosome. In total, 46 out of 52 individuals assigned to the early Bronze Age yielded sufficient genomic data for downstream analyses.

### Sequence data processing

After demultiplexing, EAGER was used to process raw aDNA sequence data. Raw reads were trimmed for Illumina adapter sequences using AdapterRemoval 2.3.0^65^. Subsequently, reads were mapped to the human reference genome hs37d5 using BWA v. 0.7.12^66^ and duplicates were removed using DeDup v. 0.12.1^61^. To analyse characteristic DNA damage in the form of G to A and C to T substitutions, mapDamage v. 2.0.9^67^ was used. The effect of postmortem DNA damage on genotyping was minimised by removing 2 bp from the 3′ and 5′ ends of reads from double-stranded UDG-half-treated libraries using the trimbam function included in bamUtils v. 1.0.13^68^. The resulting filtered bam files were genotyped with pileupCaller v.1.4.0.2^69^ by randomly calling one allele per position considering the human genome as a pseudo-haploid genome (–randomHaploid).

### Ancient DNA authentication

All libraries yielded damage patterns characteristic for aDNA, which includes short DNA fragments with an average length of 61 bp and post mortem deamination at the end of the molecules (5–20% for partial UDG treatment). We merged Shotgun, 1240 k and MT capture data for each individual, and mapped this to the revised Cambridge Reference Sequence (rCRS) for the complete human mitochondrial genome (NC 012920.1), and estimated contamination for both sexes on the mitochondrium using ContamMix^70^ (Supplementary Table A), ranging from 0.15 to 0.54%. The nuclear contamination for males was estimated using ANGSD^71^ and ranged from − 0.5 to 1.5%. Individual 2244 yielded a contamination estimate of 23%. Nevertheless, the genetic sex determination for this individual, as well as his position in the PCA are not affected by this estimate. We predicted the genetic sex by calculating the coverage on the X, Y and the autosomal chromosomes, where the X and Y coverage is normalised by the autosomal coverage, and the relative length of each sex chromosome^72^.

### DNA reference datasets

The new genotype data were restricted to two sets of reference panels, the Affymetrix Axiom Genome-wide Human Origins1 array (HO; 593,124 autosomal SNPs^73,74^) and the 1240 k panel (1.233,013 autosomal SNPs including all of the HO SNPs^38^). The number of SNPs covered at least once for each of these reference panels is given in Supplementary Tabe A.

### Genetic relatedness analysis

We estimated genetic relatedness between individuals using BREADR^42^. Individuals 2029-1 and former 2029-21 were identified as identical and therefore merged for downstream analysis. We identified 41 first-degree relationships and 24 second-degree relationships (Supplementary Table D). From groups of first-degree relatives, the individual with the highest number of SNPs on the 1240 k target region was used for further analyses.

### Assignment of uniparentally-inherited haplogroups

Trimmed Shotgun, 1240 k and MT capture reads were aligned to the revised Cambridge Reference Sequence (rCRS) for the complete human mitochondrial genome (NC 012920.1) and a consensus sequence for each individual was retrieved using Geneious v. 2019.2.3^75^. HaploGrep2^76^ was used to assign each consensus sequence to a specific mitochondrial haplogroup (Supplementary Table A). Y-chromosome haplogroups for all male individuals were assigned using the manual assignment method of Y-haplogroup calling as described in ^64^ (Supplementary Table A).

### Population genetic analysis

For genome-wide analyses the new data from this study was merged with published ancient and modern data from the Allen Ancient DNA Resource (AADR) v44.3 (https://reich.hms.harvard.edu/allen-ancient-dna-resource-aadr-downloadable-genotypes-present-day-and-ancient-dna-data). Sites on the HO panel (~ 600 k SNPs) were used for Principal Component Analysis (PCA) using the program ‘smartpca’ v. 16,000 (EIGENSOFT^77^). Principal components were computed for 1252 present-day western Eurasians from 76 different populations on which ancient individuals were projected, using the options ‘lsqproject: YES’ and ‘shrinkmode: YES’ (Supplementary Table F, Supplementary Fig. S5a). Individuals with fewer than 30,000 SNPs covered on the HO-dataset were excluded from the PCA. All other analyses were performed on the above merged data set on the 1240 k SNP panel (~ 1.24 M SNPs). We used the ADMIXTOOLS^74^ package to calculate *f*_4_-statistics. *F*_4_-statistics were calculated using ‘qpDstat’ and the ‘f4mode: YES’ function. Standard errors (SE) were computed with the default block jackknife approach and three SE are reported and plotted (Supplementary Table G, Supplementary Fig. S6).

#### Genetic admixture modeling

Ancestry modeling and ancestry proportion estimation on the 1240 k SNP dataset was performed using qpAdm in ADMIXTOOLS^62,74^. The following groups were used as a basic set of outgroups for distal modeling: Mbuti.DG, Turkey_Epipaleolithic, Iran_GanjDareh_N, Russia_MA1_HG.SG, Russia_Kostenki14, Italy_North_Villabruna_HG. For proximal modeling with relevant Bell Beaker and Corded Ware culture groups we used the OGs Mbuti.DG, Germany_EN_LBK, Lithuania_EMN_Narva, CHG, Ukraine_N, Latvia_HG and for proximal modeling as relevant contemporaneous EBA groups we used Mbuti.DG, Germany_EN_LBK, Lithuania_EMN_Narva, CHG, Ukraine_N, Latvia_HG, Germany_BellBeaker_CG, Bohemia_CW_Early as outgroups (Supplementary Table H, I, J, Supplementary Fig. S5b,c).

### Imputation

Samples were imputed using GLIMPSE with the default parameters^78,79^. Bam files were trimmed by 2 bp from the left and right to remove ancient DNA damage. We then determined genotype likelihoods from trimmed bam files using bcftools (Li, 2011) with the 1000G panel (The 1000 Genomes Project consortium, 2015) as a reference. We used GLIMPSE_impute on genomic chunks of 2,000,000 base pairs with the buffer size of 200,000 base pairs to perform imputation. We then ligated the chunks using GLIMPSE_ligate, and determined the most likely haplotypes using GLIMPSE_sample.

### IBD sharing

IBD sharing analysis was done using ancIBD^43,44^ on individuals with more than 400 k SNPs and GP > 0.99 after imputation with GLIMPSE^78,79^. We used HapBLOCK^43^ to perform the IBD sharing estimation. Imputed samples were merged, then the vcf_to_1240K_hdf command was used to convert the vcf files to the hdf5 format. The hapBLOCK_chroms command was used to perform the IBD sharing analysis for each chromosome at a time using the default parameters. Following this, only shared blocks of more than 220 SNPs per centimorgan, and shared blocks of more than 5 centimorgans were kept for data quality purposes, and used for plotting.

### Supplementary Information

Supplementary Information 1.

Supplementary Information 2.

## Data Availability

The DNA sequences reported in this paper have been deposited in the European Nucleotide Archive under the accession number PRJEB68333.

## Code availability

All of the analyses performed in this study are based on publicly available software programs. Specific version information and non-default arguments are described in the Methods.
