# Supplementary material for: Kinship practices at the early bronze age site of Leubingen in Central Germany
Source: Sci Rep. 2024 Feb 16;14:3871. doi: 10.1038/s41598-024-54462-6 (PMC10873355; doi:10.1038/s41598-024-54462-6)
Supplement: Supplementary file 1 — Supplementary Information 1. [file 41598_2024_54462_MOESM1_ESM.pdf]

Supplementary Information

Kinship practices at the early Bronze Age site Leubingen from central Germany

Sandra Penske[1*x], Mario Küßner[2x], Adam B. Rohrlach[1,4], Corina Knipper[3], Jan Novacek[2], Ainash Childebayeva[1], Johannes Krause[1], Wolfgang Haak[1*x]

This PDF file includes

1. Archaeological context of the studied individuals
    1.1. Leubingen I
    1.2. Leubingen II
2. Statistical analysis for grave goods
3. Statistical analyses of kinship
4. Population genetic analyses
5. Supplementary Table S1
6. Supplementary Figures S1 – S7
7. References

1. Archaeological context for the studied individuals

Only individuals that have DNA preservation are described in this catalogue. The archaeological excavations 09/100 and 10/111 were directed by M. Küßner. Further archaeologists involved in the field investigations were K.-P. Wechler (09/100, 10/111), I. Balfanz (09/100) and G. Brose (10/111), who prepared the documentation of features on the sites. First anthropological investigations were carried out by B. Heußner (09/100) and T. Hunold (10/111) after Ferembach 1980[1] and Rösing 2007[2]. All pictures shown in the catalogue were taken by © Thuringian State Office for Heritage Management and Archeology, Weimar, Germany.

1.1. Leubingen I

Feature 826

09/100-633, LEU021

Grave:

Flat grave, ca. 82m south-south-east of the main burial field.

Rectangular grave, ca 1.70 x 1.40m in size, preserved up to 0.26m into the ground.

Human remains:

Mature, anthropologically and genetically male individual, 45-50 years old, body height 1.71m ± 4cm. Oriented south - north, crouched on right side with the head towards south, facing east. The left hand is angled towards north where the lower jaw is (away from the body). The right hand touched the right ilium where the sacrum is.

Grave goods:

None. Shards in the filling.

Preservation:

Good preservation.

Dating:

Únětice culture.

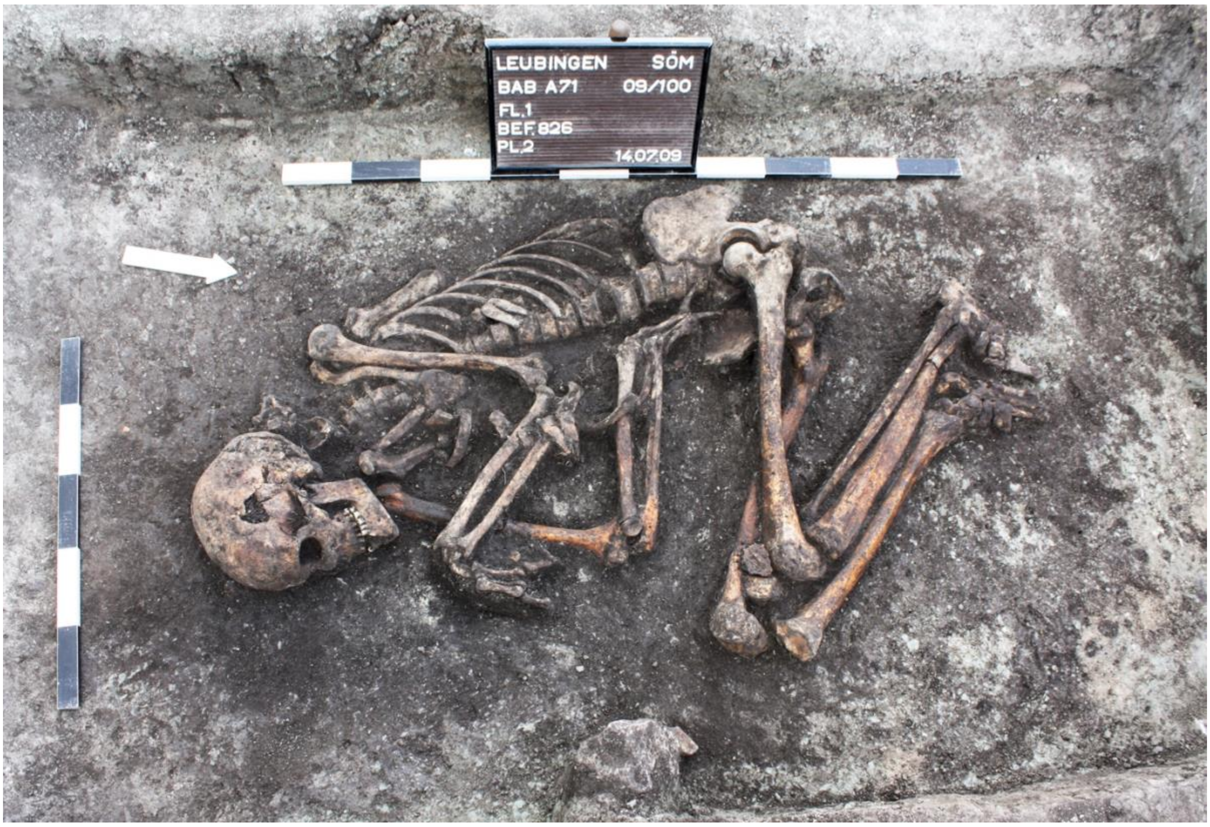

Feature 826

Feature 929

09/100-666, LEU036

Grave:

Flat grave, 111m east of the main burial ground.

Rectangular grave, 1.30 x 0.70m in size with rounded corners, preserved up to 0.15m into the ground.

Human remains:

Mature, anthropologically and genetically female individual, 50-60 years old, body height 1.66m ± 4cm. Oriented south-south-west - north-north-east, crouched on the right side with the head towards south, facing east. The lower right arm is angled towards the chest on the stomach. the lower left arm points below the spine towards the lower right arm. Both legs laid parallel below the hip, the heel bones point towards the ilium. In front of the face stood a cup with handles, another one was laying on the side near the right elbow joint.

Grave goods:

Two cups, two flintstones.

Preservation:

Mostly brittle bone substance.

Dating:

Únětice culture. MAMS 43530: 3709±21 BP, 2196-2031 cal BC.

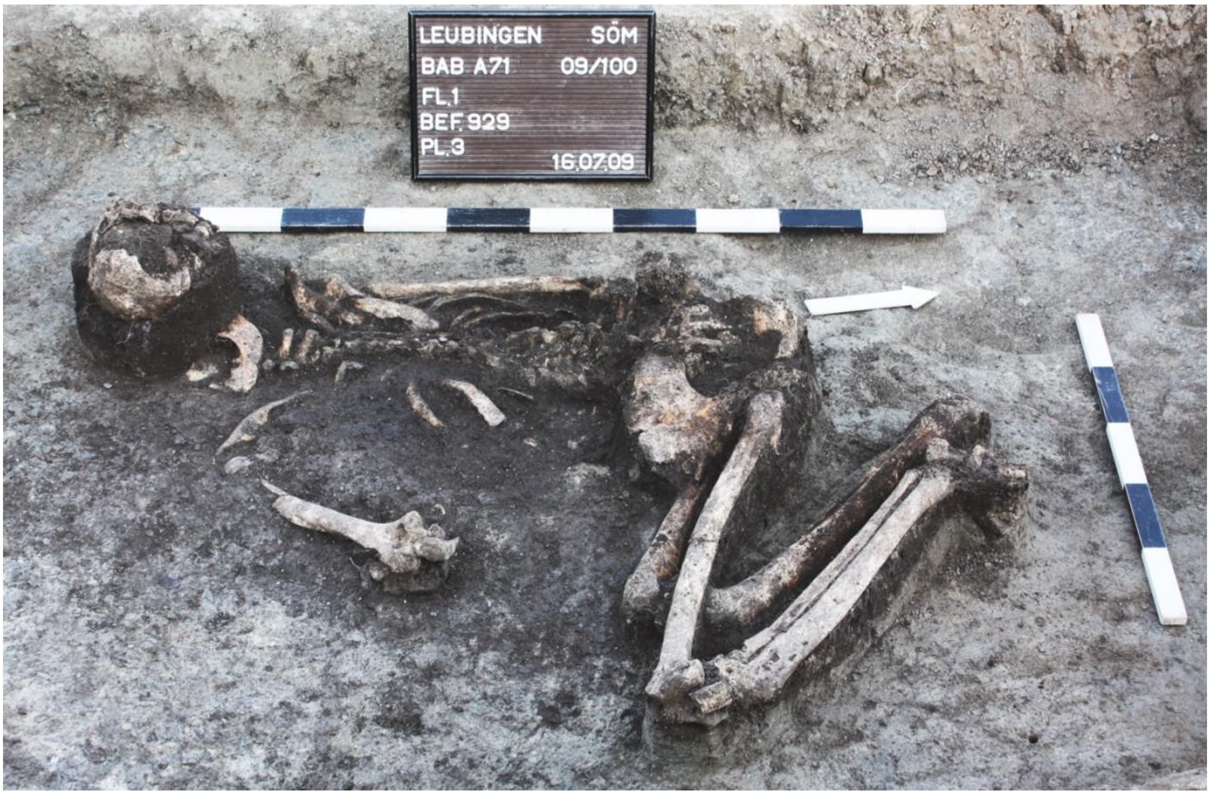

Feature 929

Feature 1380

09/100-1095, LEU038

<u>Grave:</u>

“Settlement burial”, ca. 30m southeast of the main burial ground.

Circular pit with a diameter of 1.25m, still preserved up to 0.2m below Ap horizon with irregular bottom ascending from south to north. Human remains were in the center.

<u>Human remains:</u>

Adult, anthropologically and genetically female individual, Skull.

<u>Preservation:</u>

Poorly preserved.

<u>Dating:</u>

Únětice culture. MAMS 43531: 3710±21 BP, 2197-2031 cal BC.

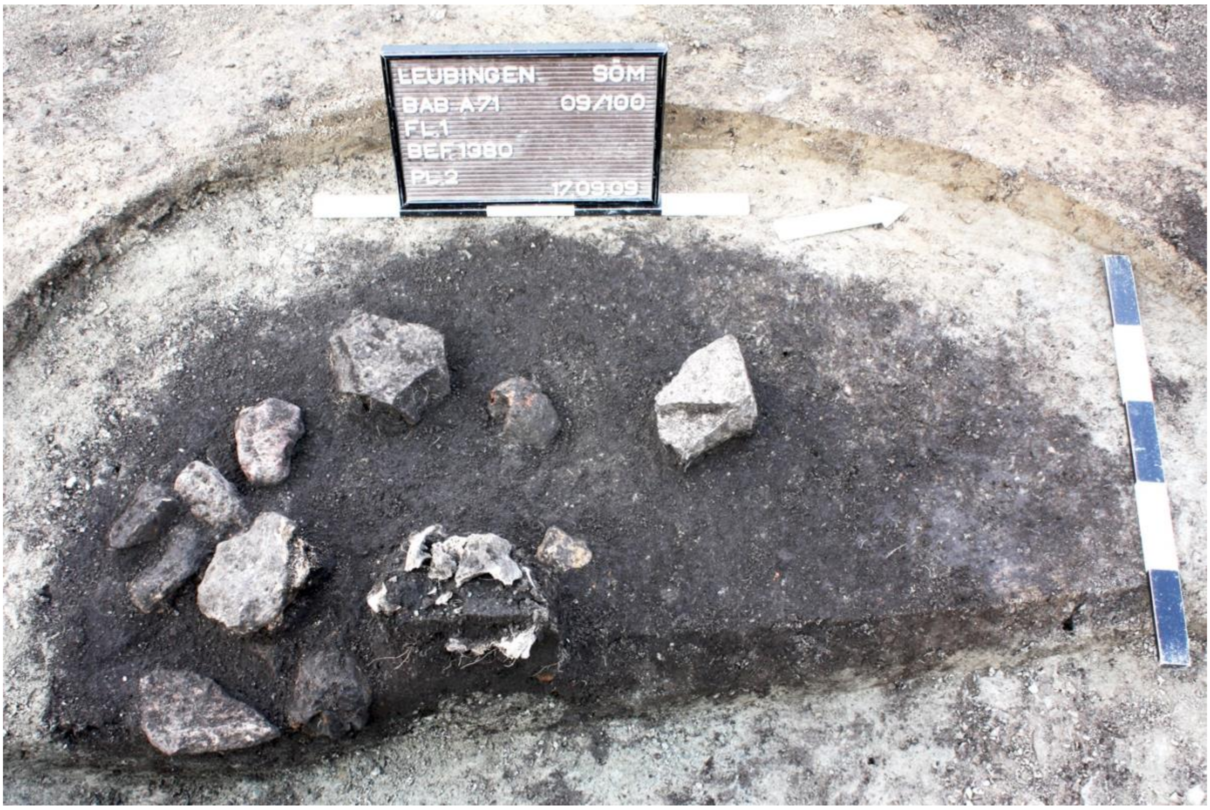

Feature 1380

Feature 1776

09/100-1530, LEU007

<u>Grave:</u>

Flat grave, ca. 67 m north-north-east of the main burial ground.

Rectangular burial pit, ca 1.35m x 0.75m in size, with slightly rounded corners, preserved up to 0.60m in the ground.

<u>Human remains:</u>

Early adult individual, anthropologically possibly female, genetically male, 18-19 years old, body height 1.62m±4cm.

Extreme crouched position, positioned on the right side, oriented S-N with head towards south and facing east. The right arm is bent in a v-form between the legs and the chest. The fingertips are orientated towards the lower jaw. The lower left arm was bent in a 90° angle in front of the lower body, the fingertip rested on the drawn-up knees.

<u>Grave goods:</u>

None.

<u>Preservation:</u>

Fully preserved skeleton.

<u>Dating:</u>

Únětice culture, MAMS 43538: 3693±21 BP, 1919-1751 cal BC.

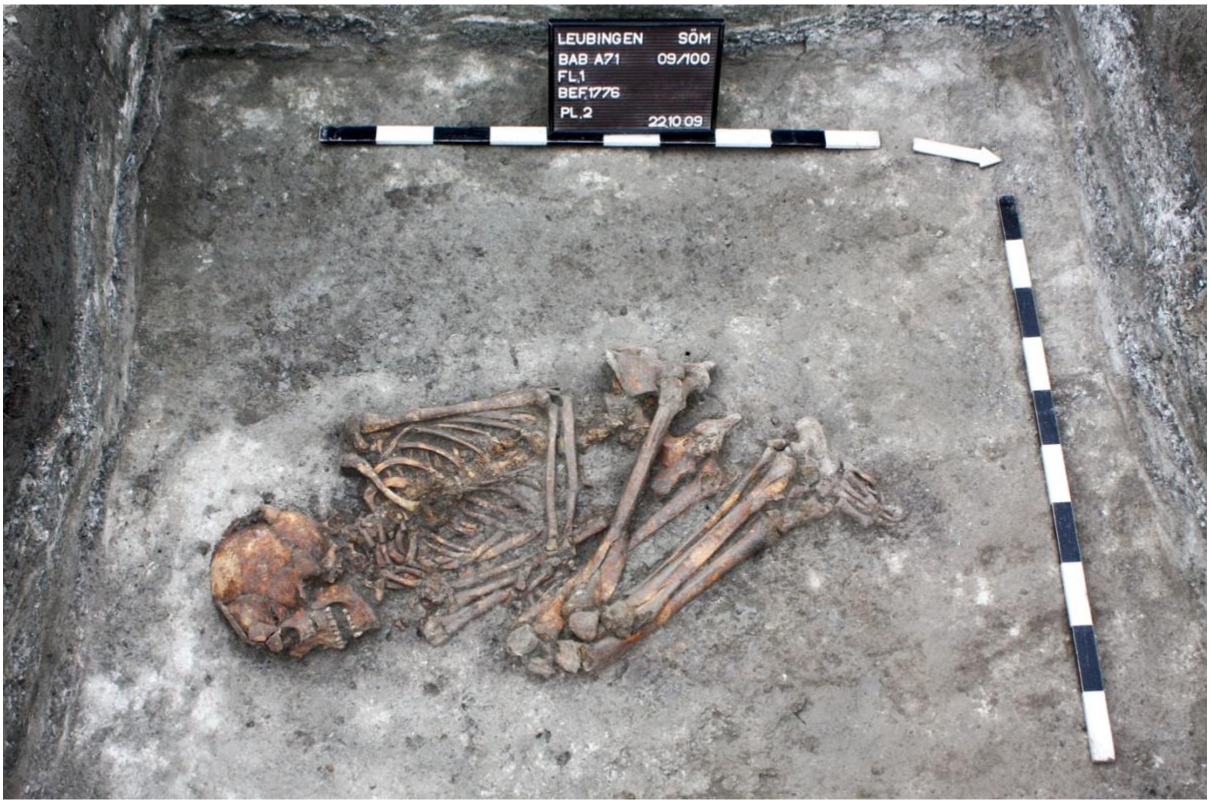

Feature 1776

Feature 1794

09/100-1545, LEU063

Grave:

Flat grave, ca. 107m north-east of the main burial ground, 1.55m north-east of feature 1791. Rectangular grave, 1.60 x 1.20m in size with rounded corners, preserved up to 0.28m into the ground.

Human remains:

Mature individual, anthropologically possibly male, genetically female individual. Oriented south-south-east - north-north-west, crouched on the right side with the head towards south, facing east. Upper legs in a strong angle. The right arm laid at a 90° angle underneath the chest, the fingertips point towards the femur/hip. Between the knees was a silex blade. The left arm was removed by the digger.

Grave goods:

Flake (?) (flintstone).

Preservation:

Good bone preservation.

Dating:

Únětice culture.

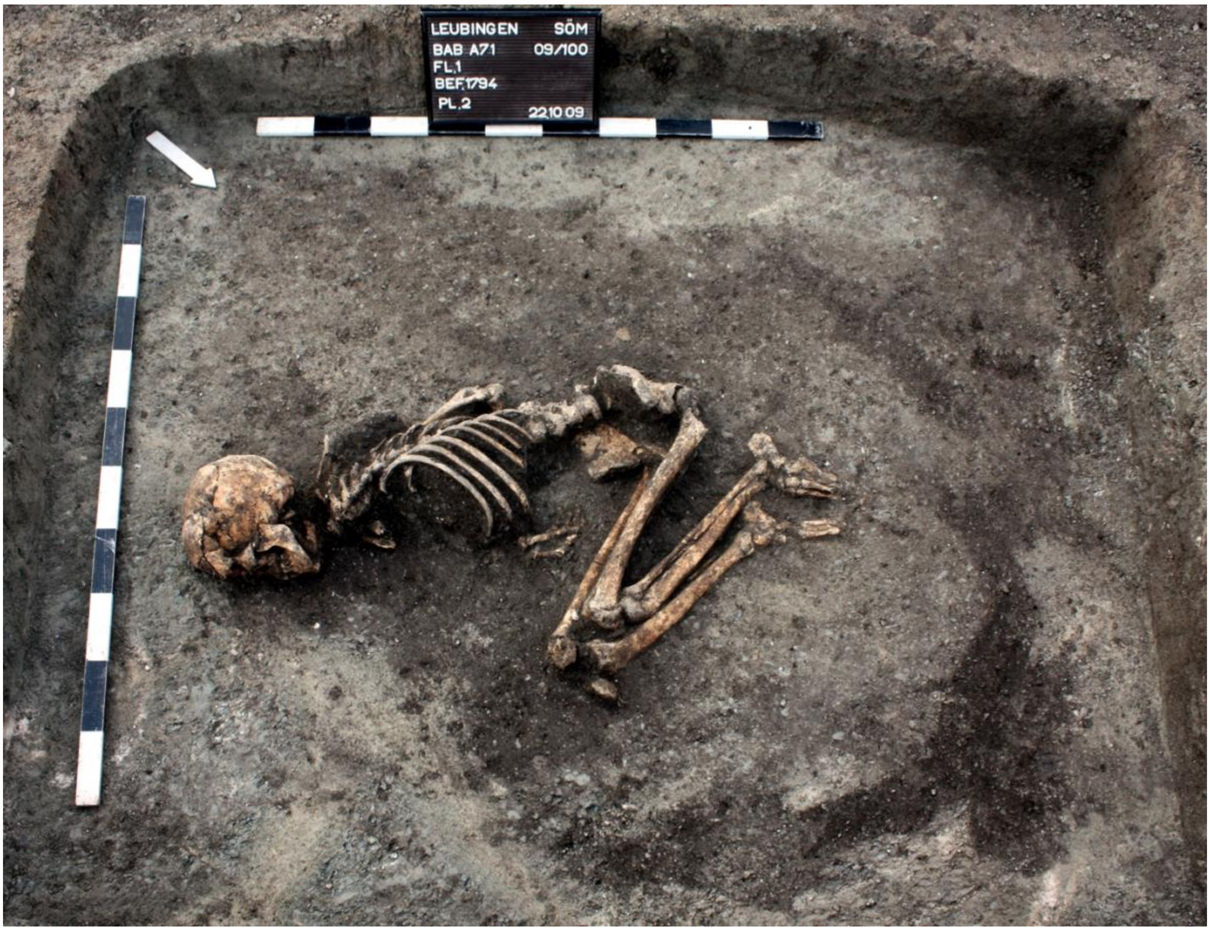

Feature 1794

Feature 1849

09/100-1630, LEU023

<u>Grave:</u>

Flat grave, 129m east of the main burial ground underneath feature 1795.

Originally oval, 0.90 x 0.60m in size.

<u>Human remains:</u>

Early adult individual, anthropologically and genetically female, 18-20 years old. Oriented north -south, crouched on the right side with the head towards north, facing west. Legs were extremely drawn towards the upper body, the left shoulder drawn forwards. Slight prone position.

<u>Grave goods:</u>

None. The fillings of booth features could not be separated and did not contain any findings.

<u>Preservation:</u>

Disturbed grave.

<u>Dating:</u>

Únětice culture. MAMS 43535: 3692±21 BP, 2192-1983 cal BC.

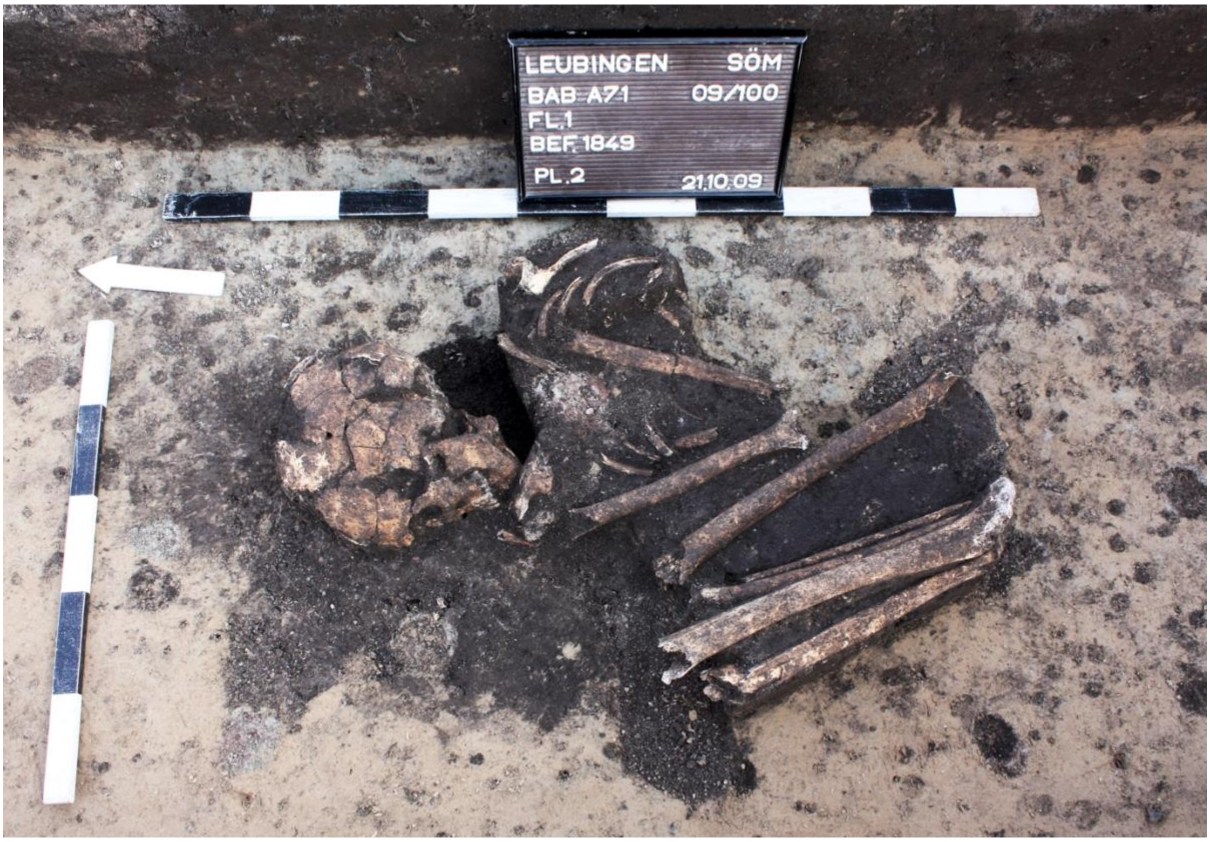

Feature 1849

Feature 2023

09/100-1697, LEU008

<u>Grave:</u>

Flat grave, ca. 3.00m north of feature 2224.

Rectangular burial pit, 1.20m x 0.80m in size with rounded corners, preserved up to 0.09m in the ground.

<u>Human remains:</u>

Mature individual, anthropologically and genetically male, >50 years old, body height 1.71m±4 cm. Oriented S-N, crouched on the right side with head to the south and facing east. The lower right arm is parallel along the upper arm at the chest. The left is bent in a 45° angle between the right arm and the upper right leg. The bones of the left hand are missing. The left leg is less drawn up to the upper body and not congruent with the right leg.

<u>Grave goods:</u>

None.

<u>Preservation:</u>

Good bone preservation.

<u>Dating:</u>

Únětice culture.

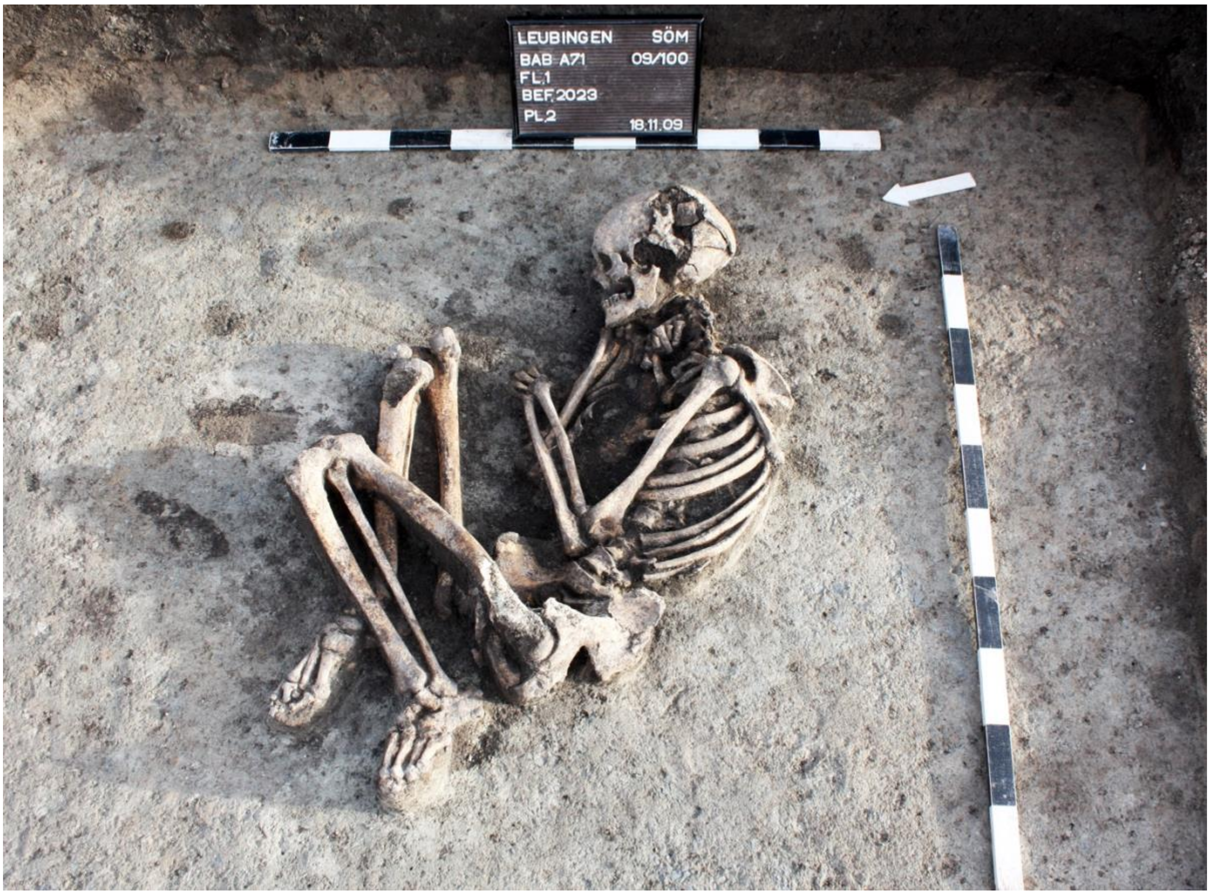

Feature 2023

Feature 2024

09/100-1703; Ind. 1, LEU010, and

09/100-1702; Ind. 2, LEU009

<u>Grave:</u>

Flat grave with stone elements, ca. 1,10m north-north-west of feature 2025.

Rectangular burial, 1.92m x 1.10m in size, preserved up to 0,58m in the ground. Remains of at least four individuals. In the south-east corner a single, reworked stone plate is present (fragment of the former stone boundary). Highly disturbed burial. In addition to krotovinas further ground disturbances are possible due to the distribution of bones (possible multiple and repeated burials or robbery?). Between planum 1 and 2 the remains of two individuals were found (named I2 and I4). Planum 2 contained another heavily disturbed grave in the western burial area (I1, ca. 0.15m under planum 1) and single bones from a fourth, juvenile individual (I3). In the eastern part of the burial area parts of the graves of I1, I3 and I4 could be documented. The bones were widely distributed in the north-eastern area and the original burial situation cannot be inferred. The side of the grave seems ‘cleared’. Noticeable is the central fragment of a reworked stone plate at the northern smaller side of the burial between I1 and I2 and I3/I4 respectively.

Human remains:

Individual 1 (I1): mature individual, anthropologically and genetically male, >50 years old. Oriented N-S, crouched on the right side with head towards south and facing east. Assignable are parts of the skull, the lower jaw, the upper arms, remains of the thorax, spine, lower arms and bones of the feet. Below the back of the head stood a small pot with a swallowtail handle.

Individual 2 (I2): Adult individual, anthropologically and genetically male, 25-27 years old

Individual 3 (I3): Infans II, undetermined sex, 8-12 years old. No DNA.

Individual 4 (I4): undetermined age and sex. Teeth only. No DNA.

Grave goods:

Small vessel (beaker), additionally ceramic shards distributed in the burial

Preservation:

Brittle bones, heavily disturbed

Dating:

Únětice culture, MAMS 34787: 3684/3633±22 BP, 2188-1978 cal BC (ind. 2024-2)

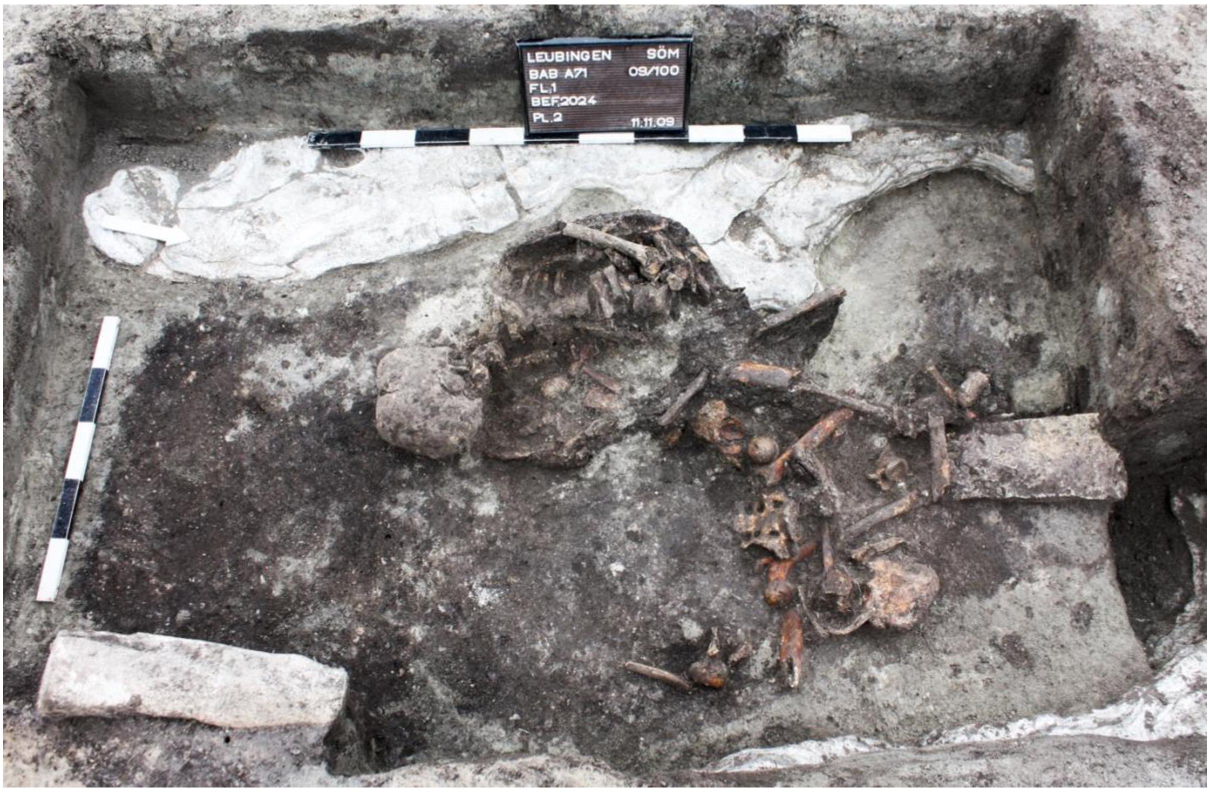

Feature 2024

Feature 2025

09/100-1708, LEU011

Grave:

Flat grave, 1.10m east of feature 2024 and 0.90m west of feature 2233 respectively.

Rectangular grave pit, 1.62 x 1.09m in size with rounded corners, preserved up to 0.4m into the ground.

Human remains:

Adult individual, anthropologically and genetically male, 26-30 years old, body height 1.75m±4cm. Oriented S-N, crouched on the right side with head to the south and facing east. The lower right arm is at a 45° angle, the hand points towards the left shoulder. The lower left arm is in a 90° angle to the upper arm and laid on the lower thoracic spine. On the level of the cranium laid a pot with handlebars on its side facing towards the skull with its opening (vessel 1). An upright standing cup with handles (vessel 2) was found 0.2m away from the upper left arm and 0.12m north of the pot respectively.

Grave goods:

Bowl with handle bars, cup

Preservation:

Good bone preservation.

Dating:

Únětice culture, MAMS 34789: 3673±26 BP, 2140-1959 cal BC.

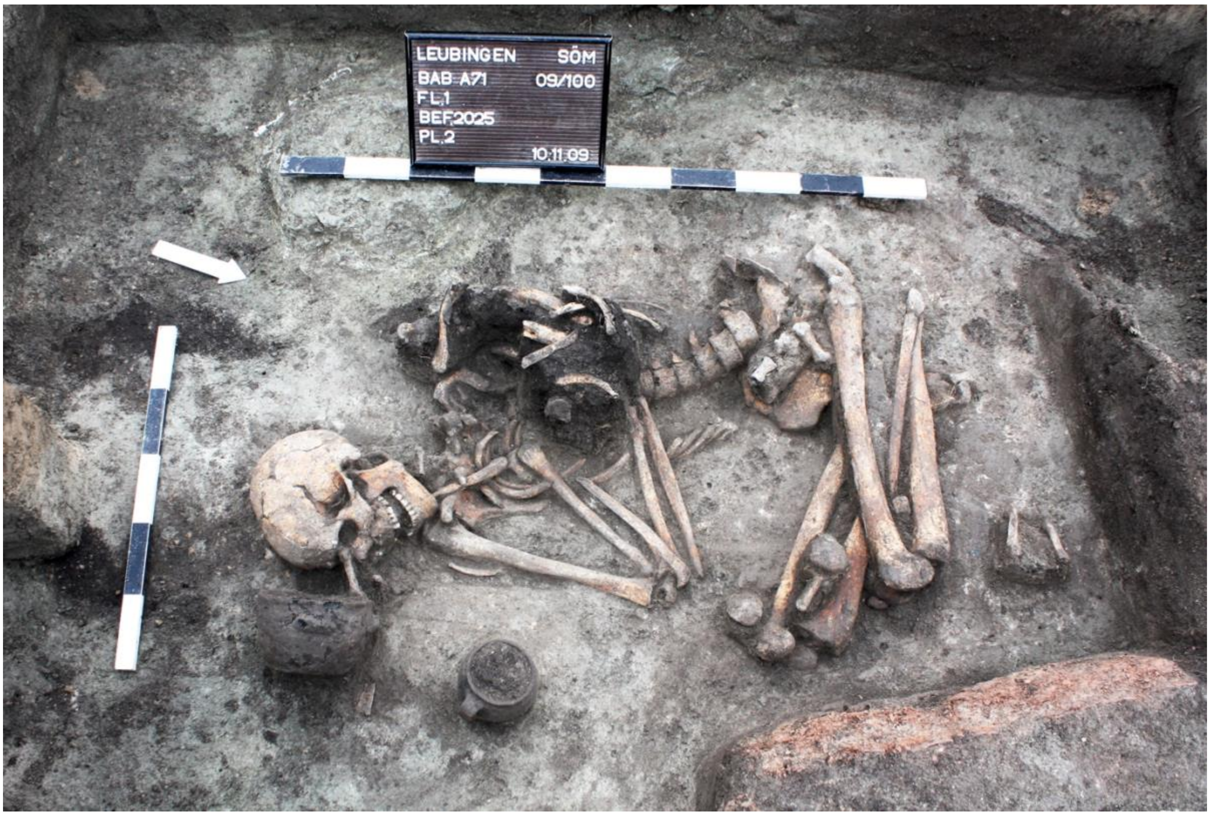

Feature 2025

Feature 2026

09/100-1716, LEU012

<u>Grave:</u>

Flat grave with stone elements, central position.

Longitudinal-oval burial pit, 2.15m x 0.75m in size, blurry boundaries, heavily disturbed by plowing. Preserved up to 0.26m into the ground. On the western side in planum 2 five medium sized stone plate fragments from the former stone boundary were found. Along the eastern side several disarticulated bone fragments from two different burials were mixed together. East of the central skull was a small bowl (vessel 1) and a broken cup (vessel 2).

<u>Human remains:</u>

Individual 1: infans II, sex anthropologically undetermined, genetically male, 10-11 years old. Remains of the skull laying on the side in the outermost south-eastern corner, facing north.

Individual 2: juvenile, likely female, 16-17 years old. Skull in the center of the grave, laying on the right side facing east (original position uncertain).

<u>Grave goods:</u>

Bowl, cup.

<u>Preservation:</u>

Poor bone preservation, heavily disturbed.

<u>Dating:</u>

Únětice culture

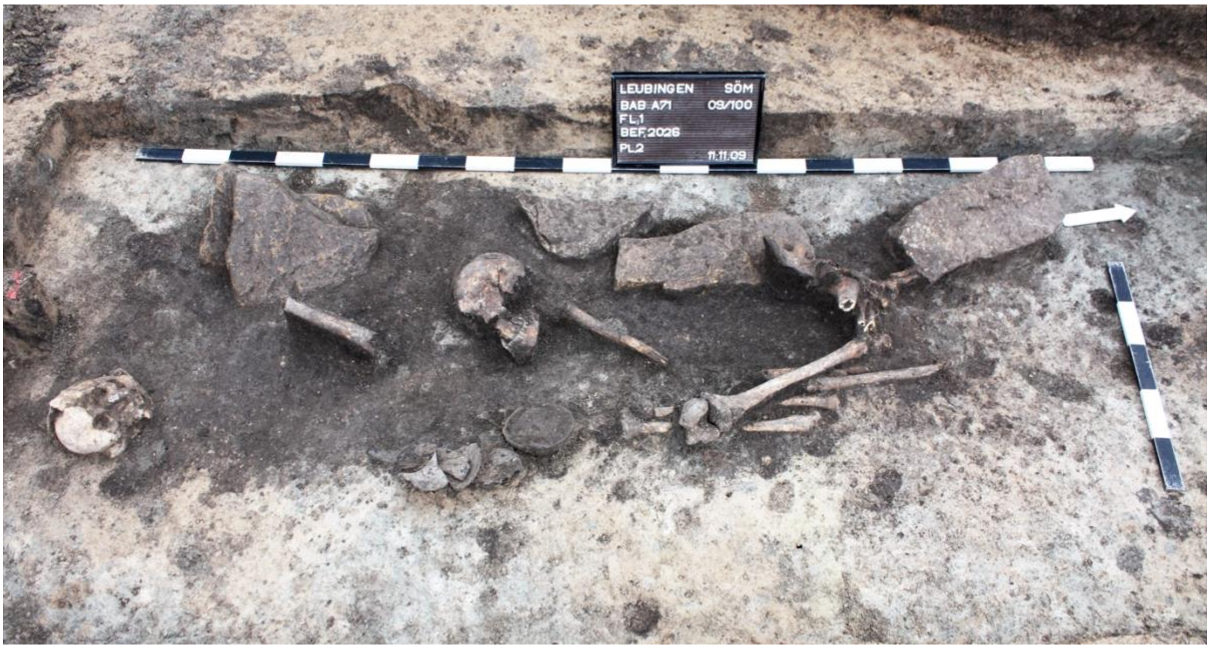

Feature 2026

Feature 2027

09/100-1785, Ind. 1, LEU029 and

09/100-1786, Ind. 2, LEU025

<u>Grave:</u>

Double burial (“Etagengrab”), ca. 1.35m east of feature 2026.
Rectangular grave, 1.50 x 0.86m in size with rounded corners, preserved up to 0.32m into the ground. Remains of two individuals, buried on top of each other.
<u>Human remains:</u>
Individual 1: Mature individual, anthropologically and genetically female, 45-50 years old, body height 1.58m ± 4cm. Oriented south - north, crouched on the right side, head towards south, facing east. The lower left arm pointed north at a 90° angle, parallel to the hip. The upper right arm laid next to the chest, the lower right arm and hand pointed towards the hip joints. The upper left leg laid at a 90° towards the ilium, on top of the right leg. Two uptight standing, heavy fragmented ceramic goods (cups with handles) were located south-south-east behind the head. I1 was laid to rest on top of the remains of a second person (I2). Two cups as grave goods. Good bone preservation.
Individual 2: Adult individual, anthropologically possibly female, genetically male, 20-30 years old. Oriented south -north, crouched on the right side with the head towards south, facing east. Skull of I2 laid underneath the right shoulder girdle of the second burial. Parts of the lower jaw and long bones of a lower arm and the legs are preserved. No grave goods. Scattered remains of utility ware and animal bones. Compared to I1 considerably worse bone preservation.
<u>Grave goods:</u>
Two beakers (I1).
<u>Dating:</u>
Únětice culture.

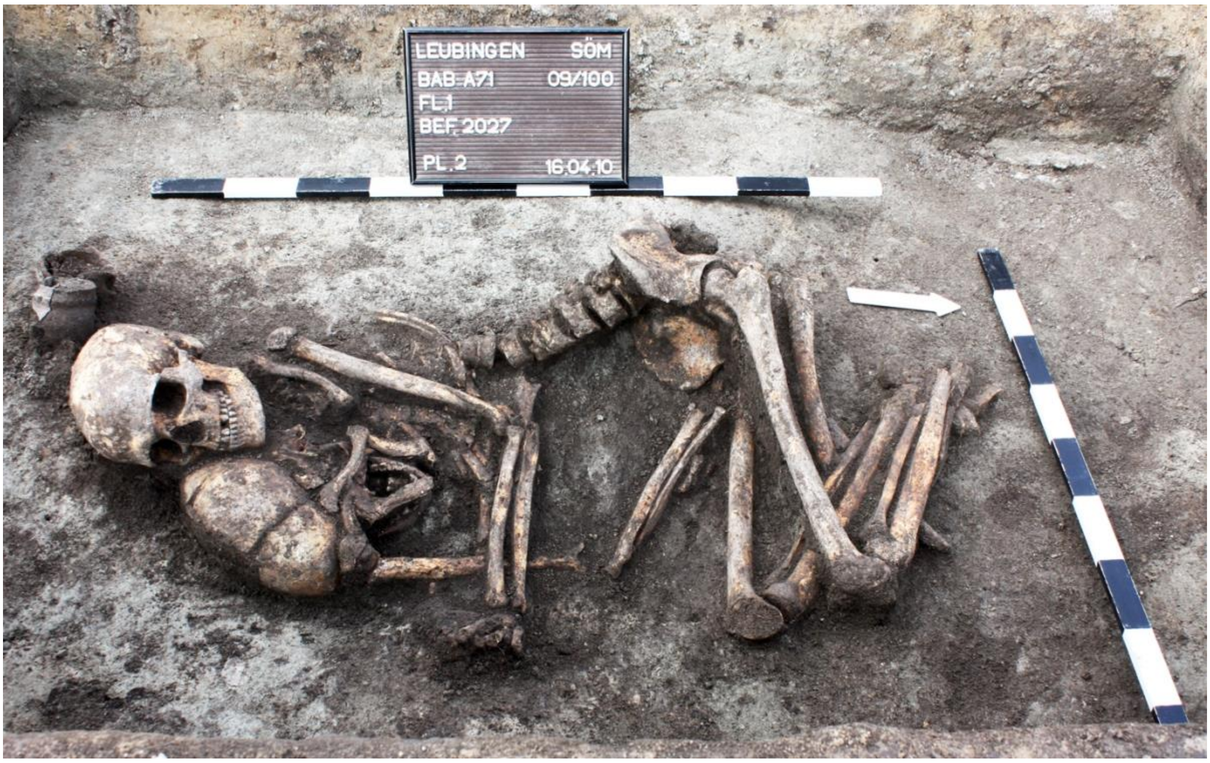

Feature 2027

Feature 2029

09/100-1787, Ind. 1, LEU051,

09/100-1788, Ind. 1, LEU026 and

09/100-1789, Ind. 2, LEU052

Grave:

Double burial (one individual on top of the other), ca. 0.70m south-west of feature 2032. Longitudinal-oval grave, 1.16 x 0.78m in size, preserved up to 0.12m into the ground. Remains of two burials on top of each other.

Human remains:

Individual 1: Infans II, anthropological sex undetermined, genetically male individual, 9-10 years old. Oriented south-south-east - north-north-west, crouched on right side, head towards south, facing east. South-east of the frontal bone an upright standing cup with a broken eye handle (vessel 1) was located. below the right parietal bone was the scapula of an animal. The left was stretched out, on the chest, pointing towards the lower join of the upper right leg. Below the lower jaw and the upper right arm was an upright standing small bowl with six handles (vessel 2). The lower left arm points towards the lumbar spine. The upper right leg (at a 70° angle to the hip) and the upper left leg (at a 90° angle to the hip) laid close together with their respective lower legs. Below individual 1 were sparse remains from an earlier burial. A cup, a bowl and a scapula as grave goods. The area around the spine is heavily disturbed due to krotovina.

Individual 2: Infans I, sex anthropologically not determined, genetically female, 5-6 years old. Oriented south-south-east - north-north-west, crouched on the right side with the head towards south-south-east, facing east. Remains of the drawn legs (upper left leg above the lower leg and underneath the hip of I1), the arms and and rip fragments below the thorax are preserved *in situ*. At the back of the head, towards north, was an upright standing cup with handles. One cup as a grave good. Heavily disturbed grave.

Grave goods:

One cup, one bow, one scapula of sheep or goat (I1).

One cup (I2).

Dating:

Únětice culture.

NOTE:

One sample taken from individual 2 turned out to be genetically the same as individual 1. 09/100-1787, Ind. 1 was in fact a sample taken from individual 1.

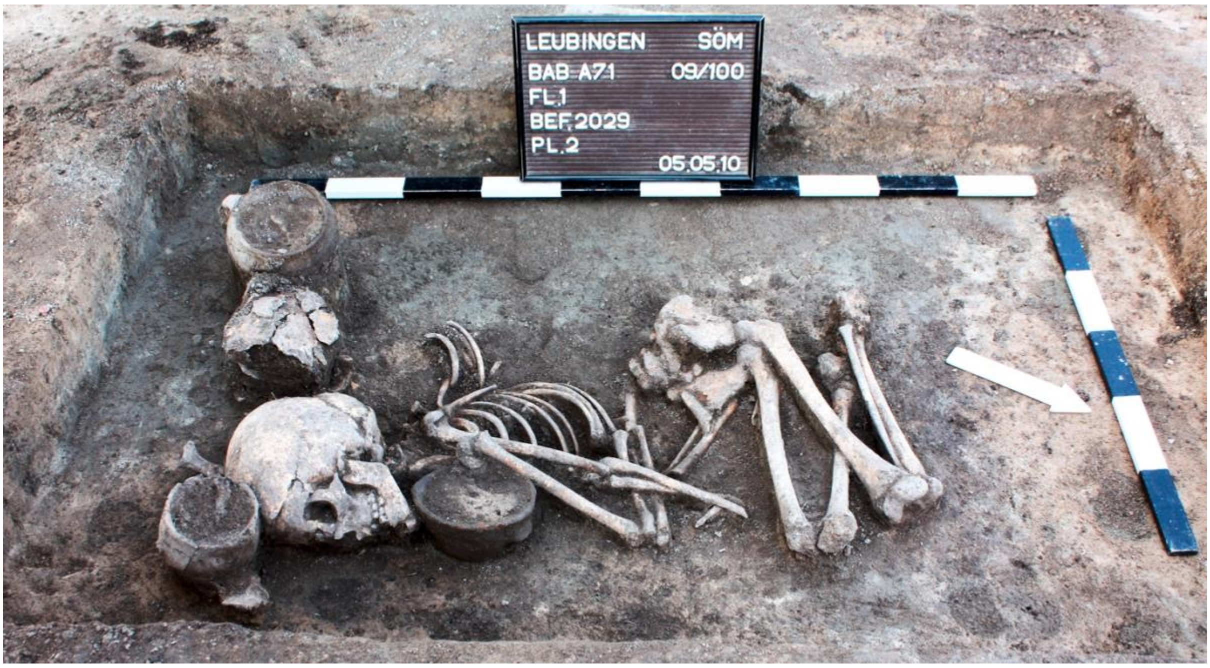

Feature 2029

Feature 2032

09/100-1792; Ind. 1, LEU013,

09/100-1794; Ind. 2, LEU014 and

09/100-1791; Ind. 3, LEU053,

<u>Grave:</u>

Bunk burial, in the north smooth transition to the feature 2031.

Rectangular grave pit, 2.10m x 1.04m in size, preserved up to 0.32m in the ground. Remains of three burials on top of each other. Planum 2 contains remains of one grave (I1). About 0.30m below is the second burial (I2). Behind or rather below I2 (planum 5) remains of a third individual (I3) were found.

<u>Human remains:</u>

Individual 1 (I1): infans II, anthropologically sex undetermined, genetically female, 11-13 years old. Oriented south-south-east - north-north-west, crouched on right side, head towards south-south-east, facing east. Only slightly crouched legs; feet not preserved. The upper right arm is parallel to the thorax, the lower arm points in a 60° angle to the hip. Remains of two incomplete, broken vessels in the area of the chest (bowl, vessel 1) and east of the right arm (cup with handles, vessel 2) respectively. Cup and bowl as grave goods. Heavily disturbed.

Individual 2 (I2): Mature individual, anthropologically and genetically female. Body height 1.59m±4cm. Oriented south-south-west - north-north-east, crouched on the right side, head towards south-south-west, facing east. The upper left arm is placed on the thorax, the lower arm points in an 80°angle towards east, to the right knee. The upper left leg is parallel to the

lower left arm, the lower leg is in an extreme crouched position and is positioned with its lower joints in the middle of the upper right leg of a third person. The upper right leg is couched in an even more extreme position and the lower leg is parallel to it. Southeast of the lower jaw was an upright standing intact cup (vessel 3) with two pierced handles. Between the cup and the lower jaw was a cervical vertebra. Noticeable is the position of three large boulders behind the skull, on the upper left arm and on the iliac bone. One cup as a grave good. Brittle bone structure. Heavily disturbed in the area of the thorax, the cervical spine and the upper right arm.

Individual 3 (I3): Mature individual, anthropologically and genetically male, 45-50 years old. Oriented south-south-west - north-north-east, crouched on the right side. Remains of the cranium, the long bones of the upper and lower extremities, as well as parts of the hip and feet. Cranium fragments of I3 located above or south of the cranium of the second burial. The upper left arm laid behind the left upper arm of I2, the lower arm is bent in a similar angle and laid underneath the lower left arm of I2. The legs of the lowermost burial are less drawn to the body compared to I2 and are less strongly developed. No grave goods. In the lowest filling are two worn out ceramic shards. Highly disturbed burial.

Grave goods:

One cup, one bowl (I1), one beaker (I2), none (I3)

Dating:

Únětice culture, MAMS 34790: 3750±22 BP, 2278-2041 cal BC (ind. 2032-1); MAMS 34791: 3730±17 BP, 2201-2038 cal BC (ind. 2032-2); MAMS 34792: 3640±28 BP, 2134-1924 cal BC (ind. 2032-3).

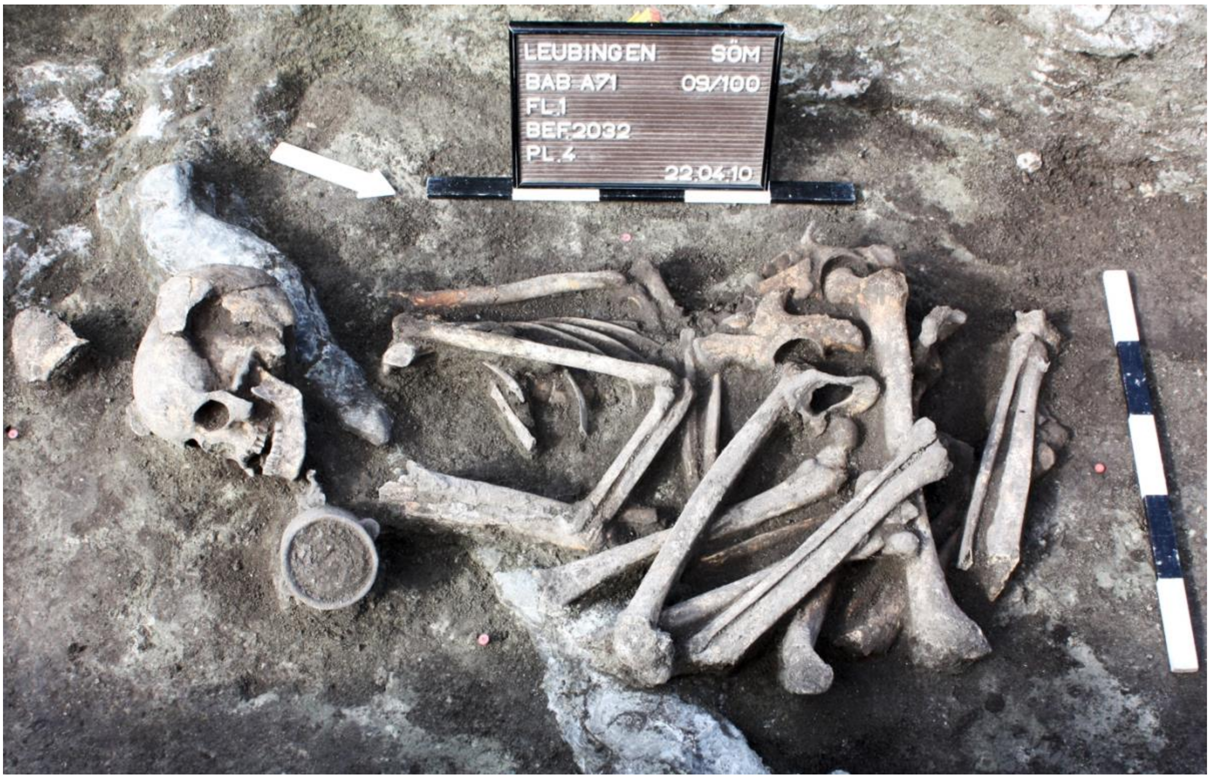

Feature 2032-2/-3

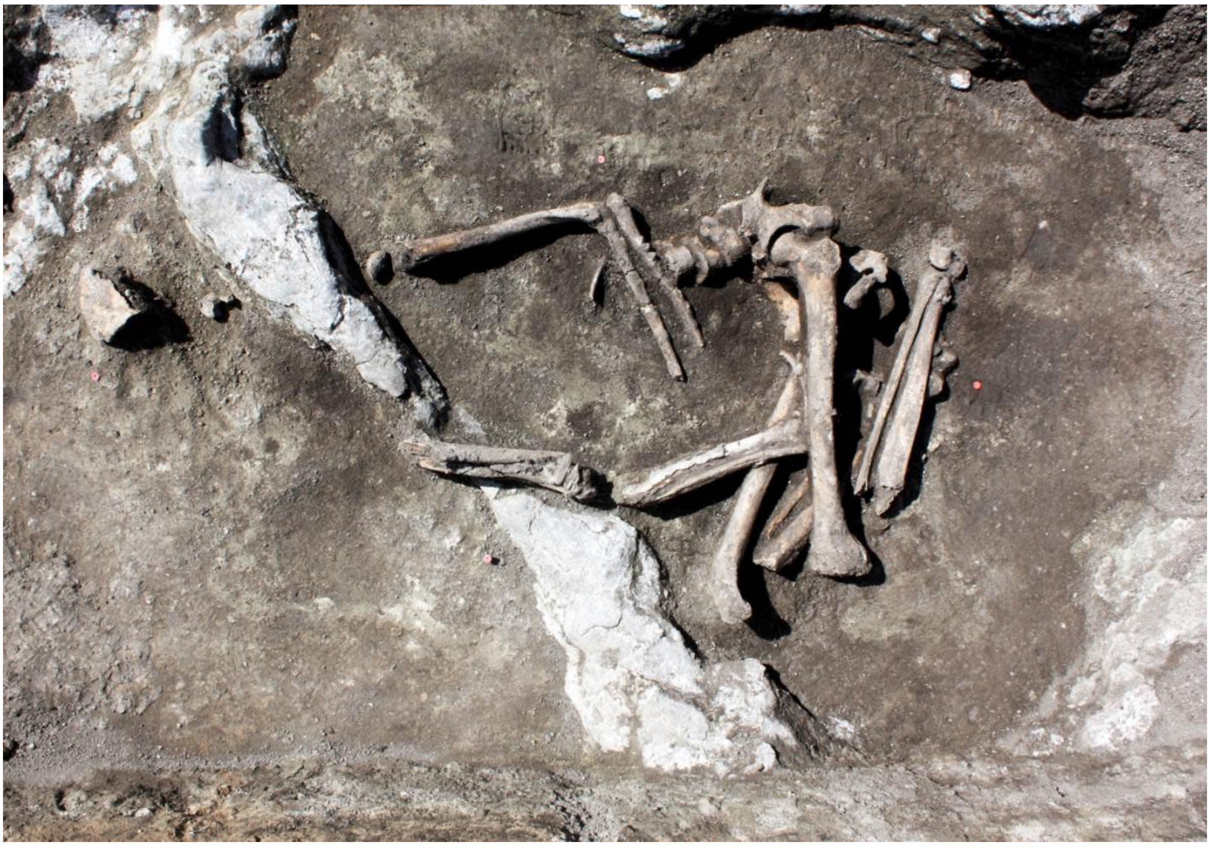

Feature 2032-3

Feature 2033

09/100-1728, LEU050

Grave:

Flat grave, directly south-west to feature 2035.

Irregular and rectangular grave, 1.76 x 1.02m in size with rounded corners, preserved up to 0.18m into the ground. Remains of two burials within the eastern part of the grave.

Human remains:

Remains of two skulls, placed within each other. 'Skull 1' (I1) laid on the right side, facing east. Directly next to it to the north 'skull 2' (I2) was laying on its calotte. It showed clear green discolouration on the left temple (first sign for bronze artefacts within the early Bronze Age burial ground). Directly west of the skull were three ceramic goods (twoo cups with handles, one pot with handles). The overall situation hints towards different burial phases or secondary burial interferences and/or grave robbery.

Individual 1: Early adult individual, anthropologically and genetically female, 18-20 years old. The left hip with femur and tibia, as well as three thoracic vertebrae were preserved in the anatomical context. Directly underneath towards south-east were the remains of another burial.

Individual 2: Juvenile (adult?!), likely male individual. Remains of two femora with one tibia and fibula respectively.

Grave goods:

Two cups, one pot, greenish coloration of the left temporal bone as indication for former copper object (I2).

Preservation:

Poorly preserved bone substance.

Dating:

Únětice culture. MAMS 34793: 3725±23 BP, 2201-2035 cal BC.

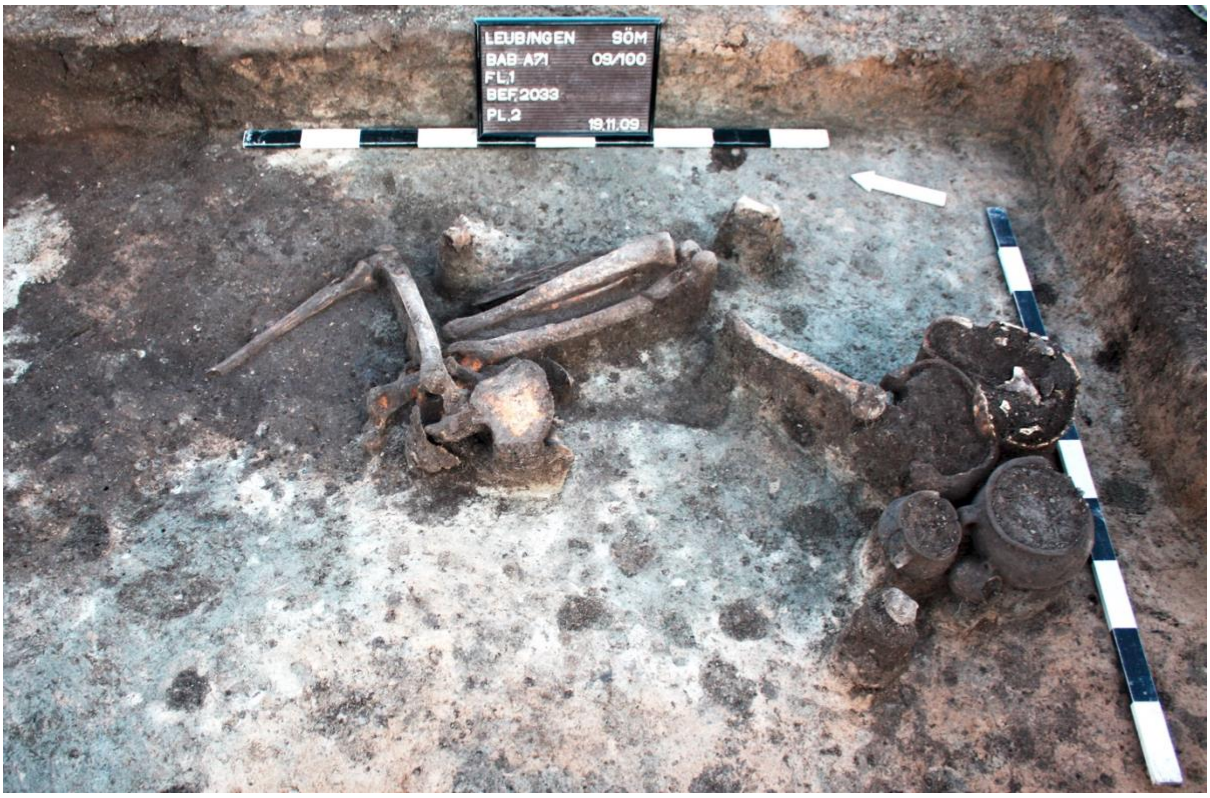

Feature 2033

Feature 2038

09/100-1797, Ind. 1, LEU055 and

09/100-1798, Ind. 2, LEU024

<u>Grave:</u>

Flat grave, ca. 0.70m west of the child burial (feature 2039).

Rectangular grave, 1.23 x 0.82m in size, with rounded corners. In the north with a half-circle indent. Preserved up to 0.28m into the ground. Remains of two individuals.

<u>Human remains:</u>

Individual 1: Infans I, anthropological sex undetermined, genetically male, 5-7 years old. Oriented south - north, crouched on the right side with the head towards south, facing east. In front of the head deposition of a large cup with handles. The lower right arm is heavily bent and points towards the cup. The lower left arm originally pointed towards the cup as well. Disturbed in the area of the spine and the legs. Below the feet, in the area of the indent was a second infant cranium (I2) without the lower jaw.

Individual 2: Infans I, anthropological sex undetermined, genetically male, 6-7 years old. Skull laid on the right side, facing east.

<u>Grave goods:</u>

Cup (I1), greenish coloration of the left temporal bone as indication for former copper object (I2).

Preservation:

Heavily disturbed.

Dating:

Únětice culture.

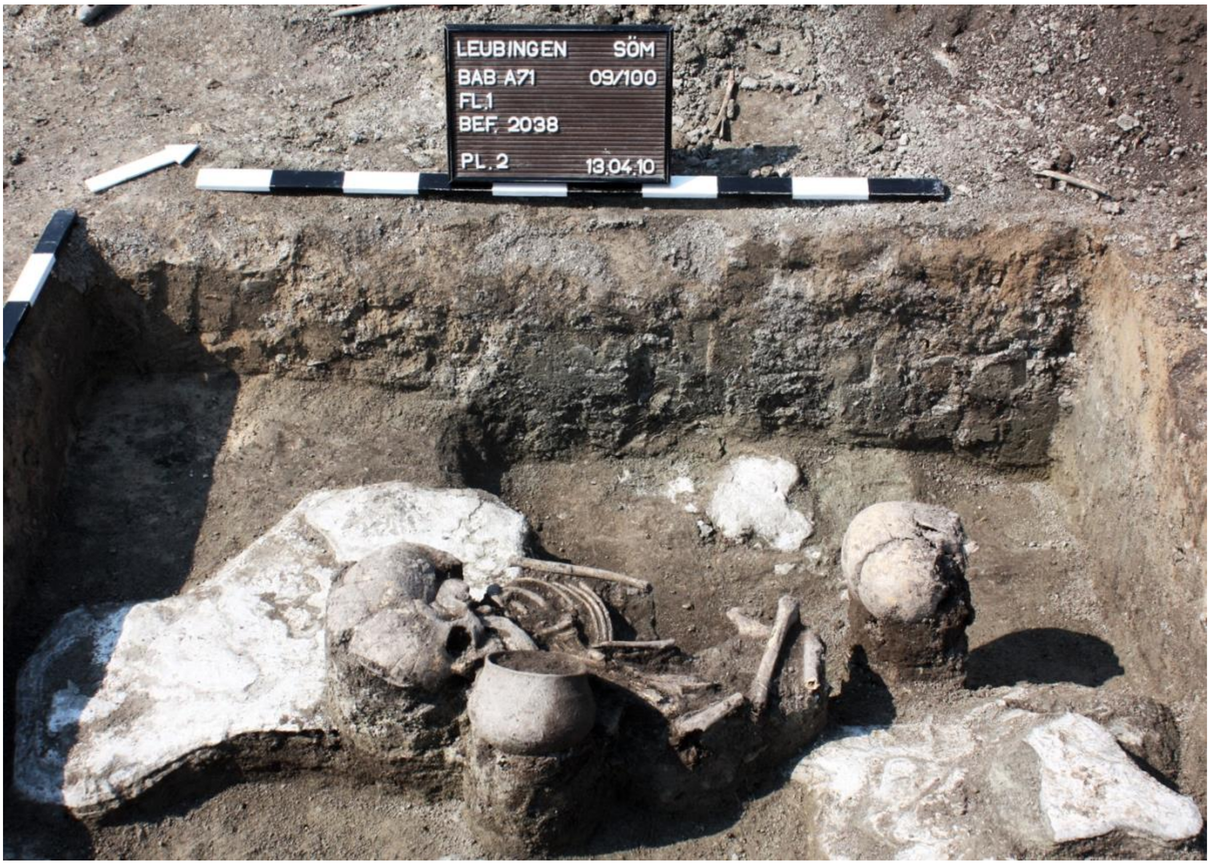

Feature 2038

Feature 2039

09/100-1800, LEU030

Grave:

Flat grave with stone elements, ca. 0.70m east of feature 2038, disturbed by a pit (feature 2040) in the north.

Rectangular grave with rounded corners, 1.15 x 0.53m in size, preserved up to 0.13m into the ground. In the north-east corner remainders of a stone boundary (two small rectangular stone plates).

Human remains:

Early adult individual, anthropologically and genetically female, 20-25 years old. Oriented south - north, crouched on the right side with the head towards south, facing east. Only remains of the skull, the rips and the long bones preserved. Four small ceramic vessels (small to smallest cups with handles) were originally standing upright in front of the head and the

chest, the handles pointing towards the face. A copper ring was placed at the left ear (green coloring on the left parietal bone also).

<u>Grave goods:</u>

Four cups, one copper wire ring.

<u>Preservation:</u>

Poor bone preservation.

<u>Dating:</u>

Únětice culture.

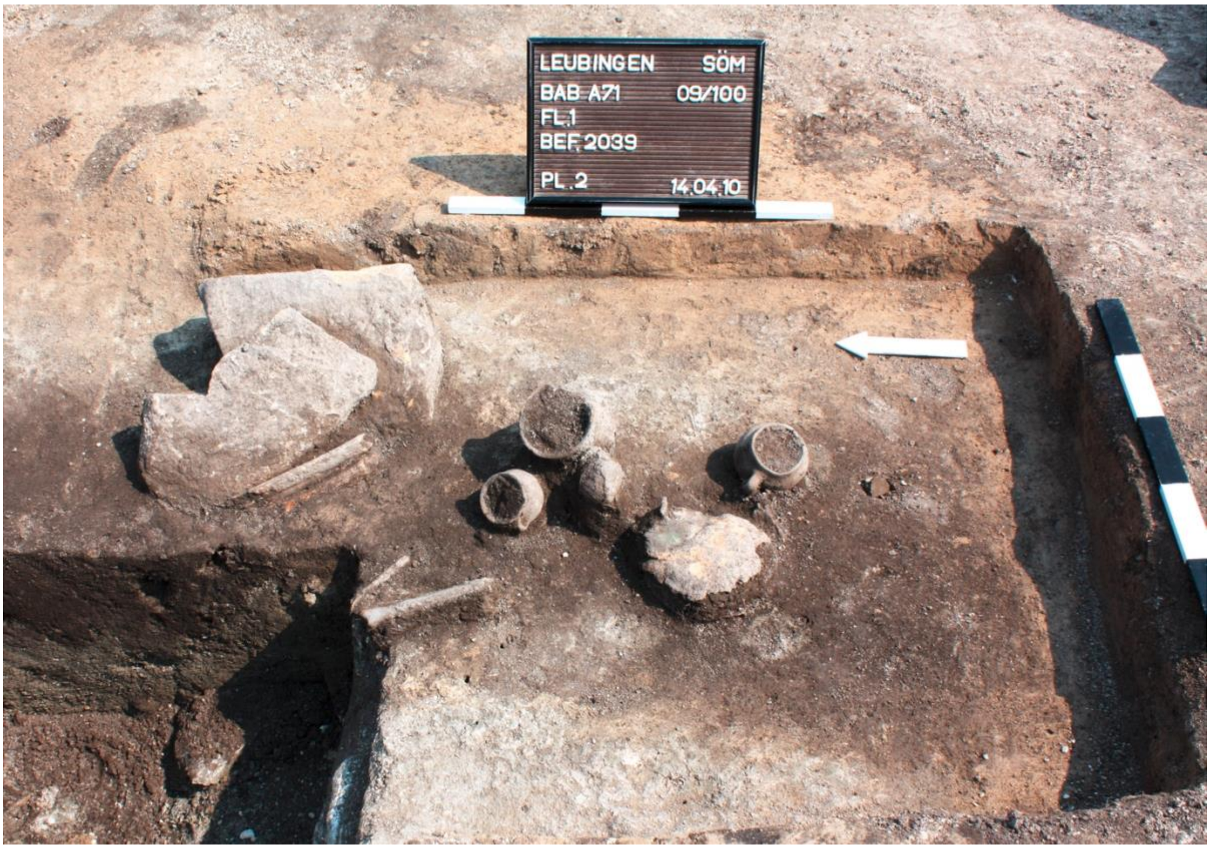

Feature 2039

Feature 2044

09/100-1733, Ind. 2, LEU035

<u>Grave:</u>

Secondary burial in black soil, north-west and above feature 2045.

Irregular, 1.20 x 0.67m large grave. Remains of two inhumations. The upper layer consists of a single long bone and skull remains without any anatomical context.

<u>Human remains:</u>

The remains of two individuals were probably laid to rest at the same time; the articulated bone situation could not be determined. West of the bones were the remains of an upright standing large, handleless cup. In direct connection was the slightly lower inhumation of feature 2045

(stone cist). Both features refer back to each other (2044 as a secondary burial to feature 2045?). Mixed bone remains in planum 2.

Individual 1: Adult, likely male, ca. 38 years old.

Individual 2: Mature, probably male, over 50 years old.

<u>Grave goods:</u>

Bottom part of a spherical vessel.

<u>Preservation:</u>

Crumbly bone substance.

<u>Dating:</u>

Únětice culture. MAMS 34794: 3687±23 BP, 2191-1979 cal BC.

<u>NOTE:</u>

Individual 1 from feature 2044 is archaeologically and anthropologically likely the same as individual 2 in feature 2045.

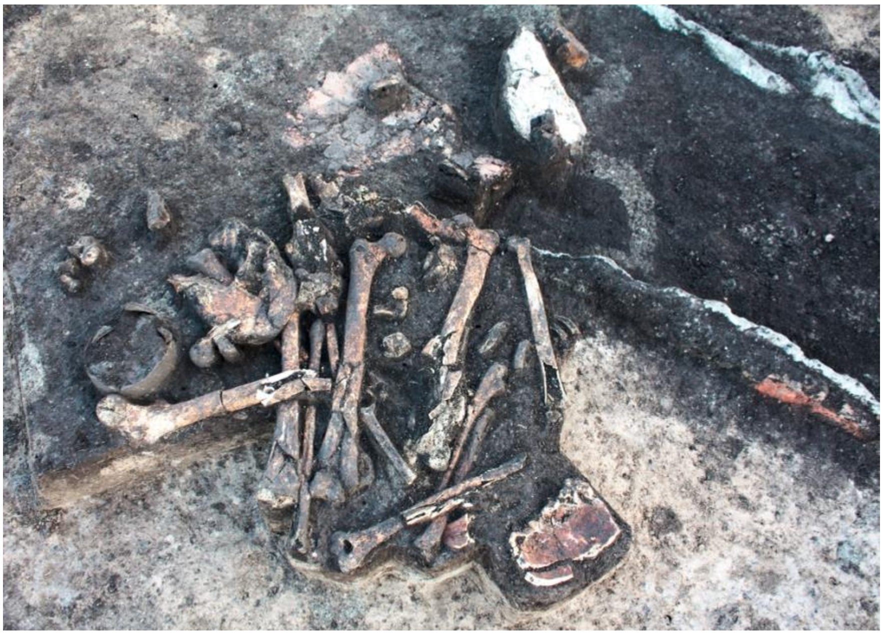

Feature 2044

Feature 2045

09/100-1743; Ind. 1, LEU064 and

09/100-1737; Ind. 2, LEU065

<u>Grave:</u>

Stone cist, directly east of feature 2044, 0.70m west of feature 2169.

Rectangular grave with vertical walls, 1.80 x 1.04m in size, preserved up to 0.40m into the ground. Striking 1.28 x 0.84m large surround with large, pointy rock plates.

<u>Human remains:</u>

Mature individual, anthropologically possibly female, genetically male, ca. 50 years old. Oriented south - north, crouched on the right side with the head towards south, facing east. Extremely drawn and angled right knee, up until the lower ribs. The left leg was lower, the upper leg was positioned at a 90° angle to the hip above the right leg. The left arm had a similar angle as the right leg. The left arm laid on the chest, the hand pointing towards a ceramic good. The lower right arm was parallel to the upper arm along the rib cage. The carpalia of the right hand laid on the clavicle. Parts of the thorax, the spine, the sacrum, the left shoulder girdle and the sternum were disturbed. At the height of the shoulder girdle, between the lower right arm and the eastern stone surround were two upright standing vessels (cup with handles (1) and a small dish (2)). Between the right ilium and the upper right leg was a perforated mussel shell. Behind the head was a fossil stone but it was likely lost during the washing process. Isolated bones from a second individual (I2), aged around 38 years, anthropologically possibly male, genetically female, were found in the grave.

Grave goods:

Two bowls (one uncertain in assignment), double perforated shell disc.

Preservation:

Good bone preservation, disturbed in parts.

Dating:

Únětice culture. MAMS 34795: 3678±24 BP, 2141-1973 cal BP.

NOTE:

Individual 2 from feature 2045 is likely the same as individual 1 from feature 2044.

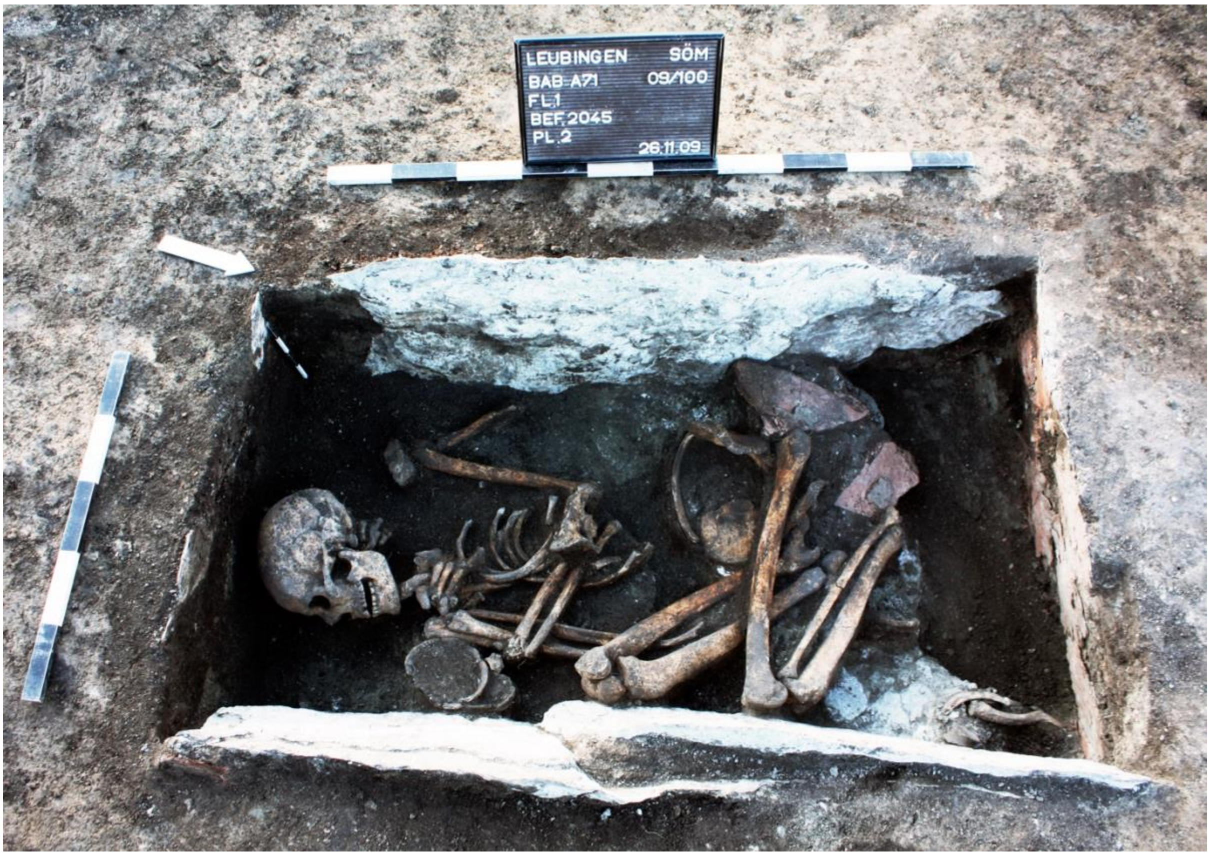

Feature 2045

Feature 2047

09/100-1748, LEU032

Grave:

Flat grave in black soil, ca. 0.8m north-north-east of feature 2044.

Ovale grave, 1.18 x 0.87m in size, preserved up to 0.15m into the ground.

Human remains:

Infans II, anthropological sex undetermined, genetically female individual, 9-10 years old. Oriented north-west - south-east, crouched on the right side with the head towards south, facing east. Legs extremely drawn to the chest. The lower arms are drawn in front of the chest and the fingers of both hands reach towards the neck. The elbow joint laid on the lower end of the femur directly behind the right knee. The head is moved backwards towards the neck. The position of the arms and the skull give the impression of a violent death (maybe death by suffocating?). The skeleton is in anatomically correct order with the exclusion of the cervical vertebra.

Grave goods:

None. Remains of three adult teeth and of a cattle bone in the filling.

Preservation:

Exceptionally well preserved.

Dating:
Únětice culture.

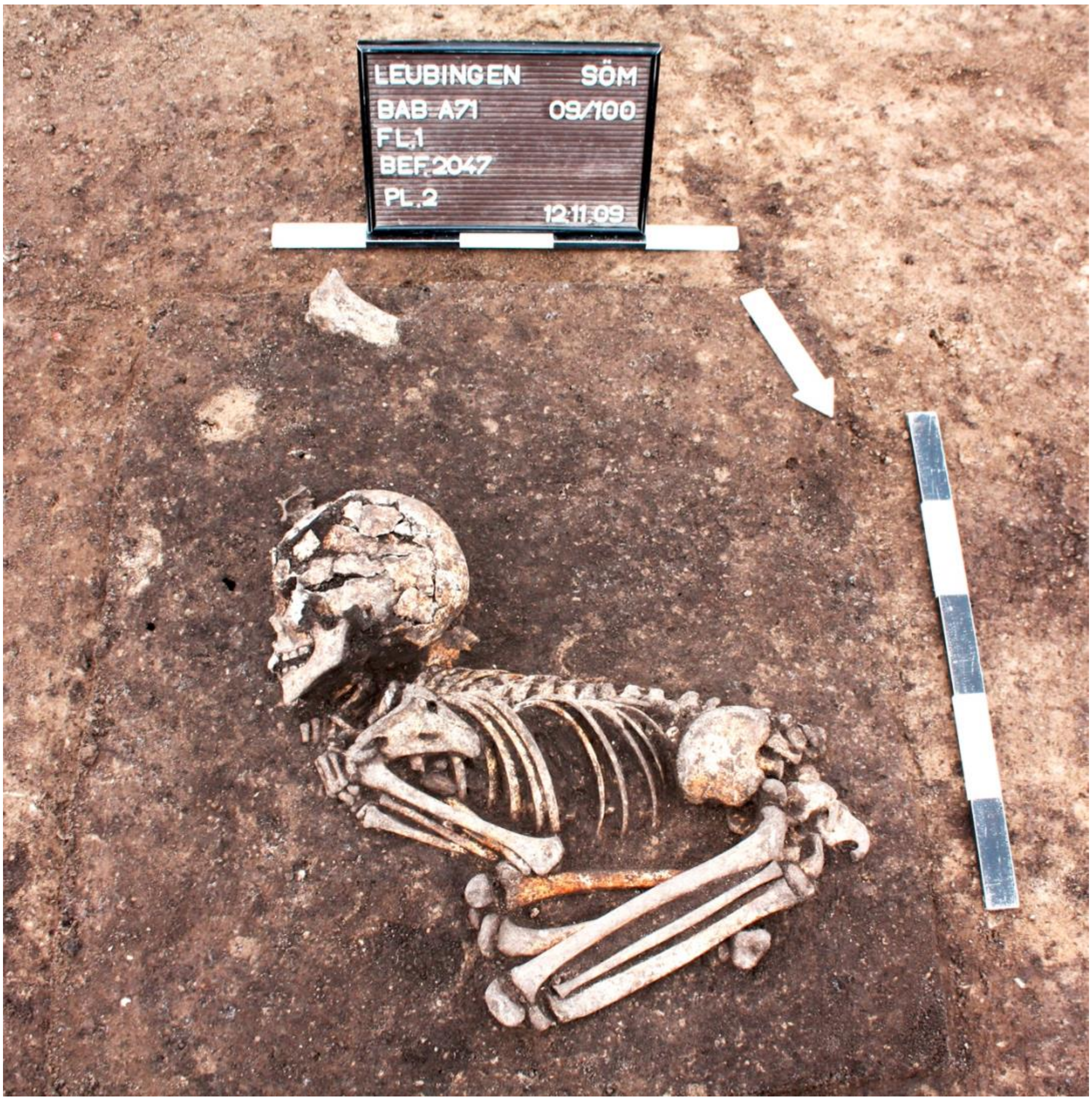

Feature 2047

Feature 2088

09/100-1773, LEU027

Grave:

Flat grave, ca. 26m south-west of the main burial ground.

Rectangular grave, 0.65 x 0.45m in size, in the pits of features 2020/2021, preserved up to 0.10m into the ground.

Human remains:

Adult individual, anthropologically possibly female, genetically male, 18-20 years old. Oriented south - north, originally couched on the right side. Only the skull is fully preserved. Remains of isolated ribs and vertebrae fragments.

Grave goods:

None.

Preservation:

Poorly preserved.

Dating:

Únětice culture. MAMS 43536: 3554±21 BP, 2008-1776 cal BC.

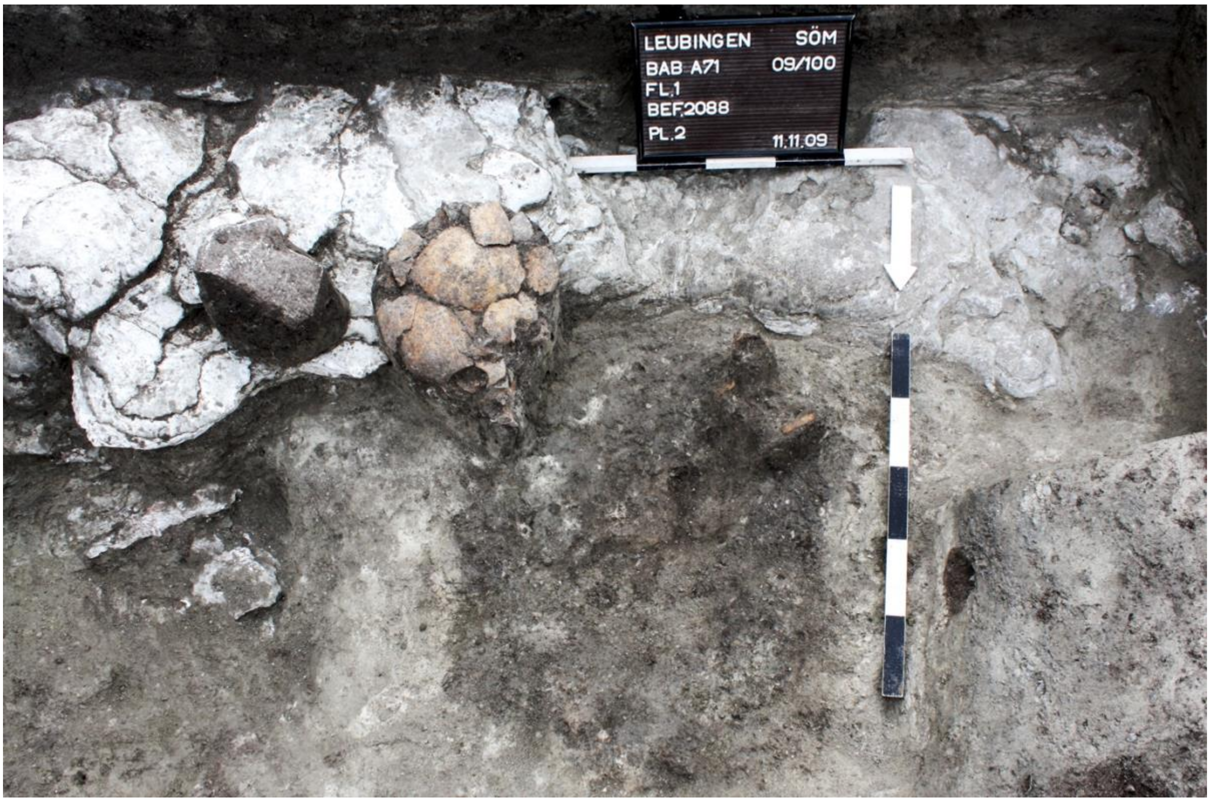

Feature 2088

Feature 2169

09/100-1802, LEU056

Grave:

Flat grave, ca. 0.80m east of feature 2045.

Oval grave, 1.30 x 1.20m in size, preserved up to 0.32m into the ground.

Human remains:

Adult individual, anthropologically and genetically male, 25-30 years old, body height 1.72m ± 4cm. Oriented south - north, crouched on the right ride with the head towards south, facing east. Slight supine position, the lower left arm points to the upper left arm at a 90° angle, parallel to the pelvic girdle towards east. The upper right arm laid along the chest; the lower right arm and the hand were stretched towards the left hip joint. The upper left leg laid above the right leg at a 90° angle towards the ilium. West of the left shoulder joint was a large, upright standing cup with handles with the handle facing north-east. Preservation of a bony needle

east of the knee, a bony awl below the upper right tibia joint and a triangular flint arrow head between the ribs of the left thorax.

Grave goods:

Cup, bone pin, bone awl, triangular arrowhead (flint stone), further ceramic shards and animal bones in the filling.

Preservation:

In the area around the shoulders and spine heavily disturbed by krotovina.

Dating:

Únětice culture.

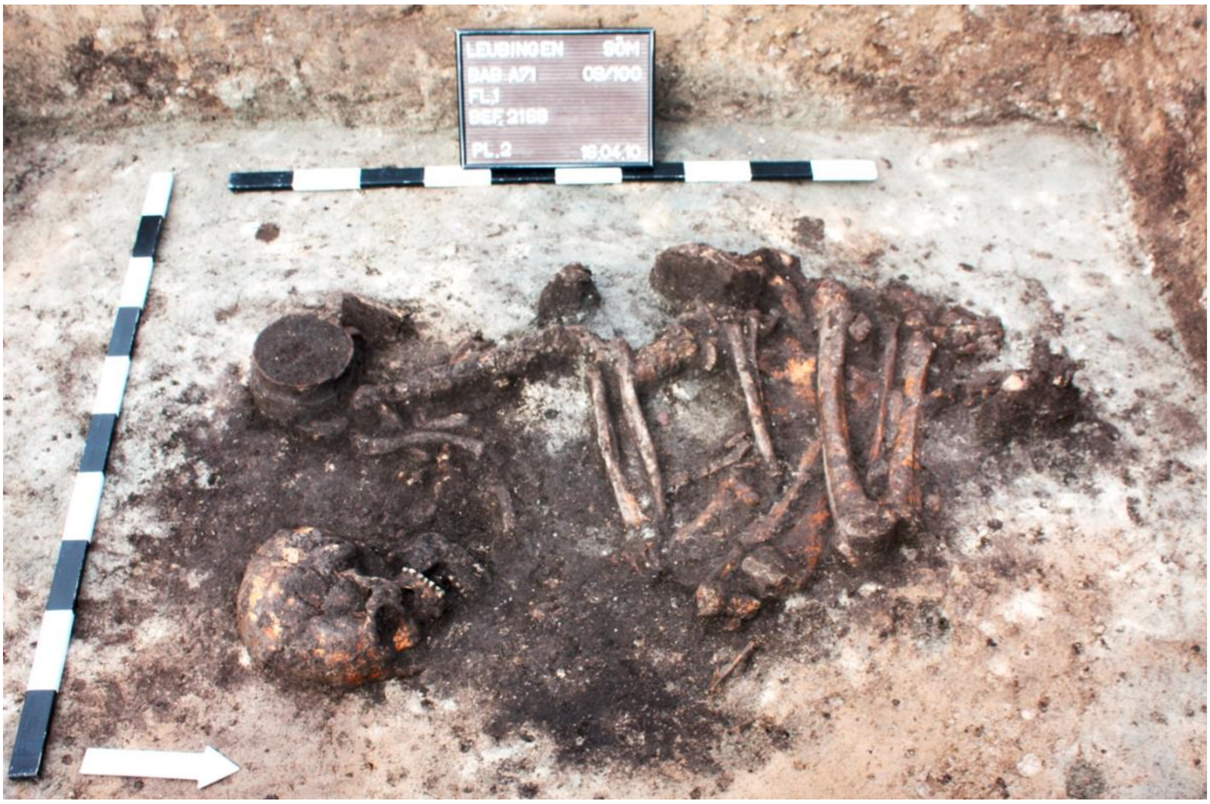

Feature 2169

Feature 2219

09/100-1807, LEU015

Grave:

Flat grave, around 0.70m west of feature 2565.

Irregular oval, ca. 1.08 x 1.04m large grave (in planum 2 oval, 0.75 x 0.65m), preserved up to 0.25m into the ground.

Human remains:

Infans II, anthropological sex undetermined, genetically male, 10-12 years old. Oriented southeast - northwest, crouched on right side with head towards southeast facing northeast. Upper arm in south - north orientation, lower arm drawn towards body in v-shape (located above the upper right arm), the left hand was originally located in the area in front of the lower

jaw. The right upper arm laid parallel to the left upper arm. The left lower arm faces west towards the lumbar spine in a 90° angle, parallel to the left upper leg. Both legs were at a 90° angle towards the hip, the lower legs parallel to the upper legs.

Grave goods:

No grave goods.

Preservation:

Brittle to good bone substance. Disturbed by krotovinas.

Dating:

Únětice culture, MAMS 43537: 3686±21 BP, 2141-1980 cal BC.

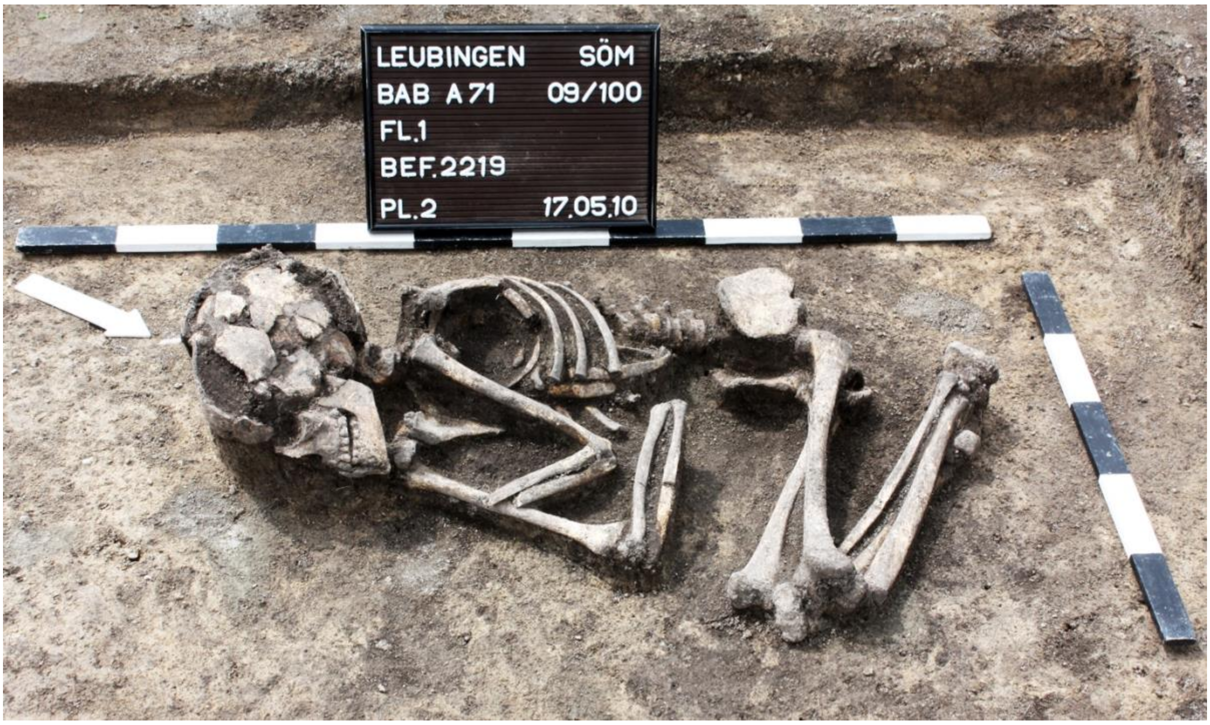

Feature 2219

Feature 2220

09/100-1808, LEU016

Grave:

Flat grave.

Elongated-oval, 1.16 x 0.72m large grave, preserved up to 0.32m into the ground.

Human remains:

Adult individual, anthropologically and genetically female, 26-30 years old. Body height 1.58m ± 4cm. Oriented south-south-east - north-north-west, crouched on right side, head towards south-south-east, facing east. The left shoulder area and the upper arm are disturbed. The left lower arm was parallel to the upper arm, the hand was touching the lower jaw. The upper right arm laid parallel between the thorax and lower arm; the right hand was underneath the

upper/lower jaw. Both legs were drawn extremely towards the right arm/the thorax. The elbow joint touched the left knee. The tips of the feet face north. The spine was heavily disturbed. The sacrum and the lumbar vertebrae are turned upside.

Grave goods:

None. One shard (early Bronze Age) was found in the fillings.

Preservation:

Bones relatively well preserved. Heavily disturbed grave.

Dating:

Únětice culture. MAMS 43538: 3693±21 BP, 2191-1984 cal BP.

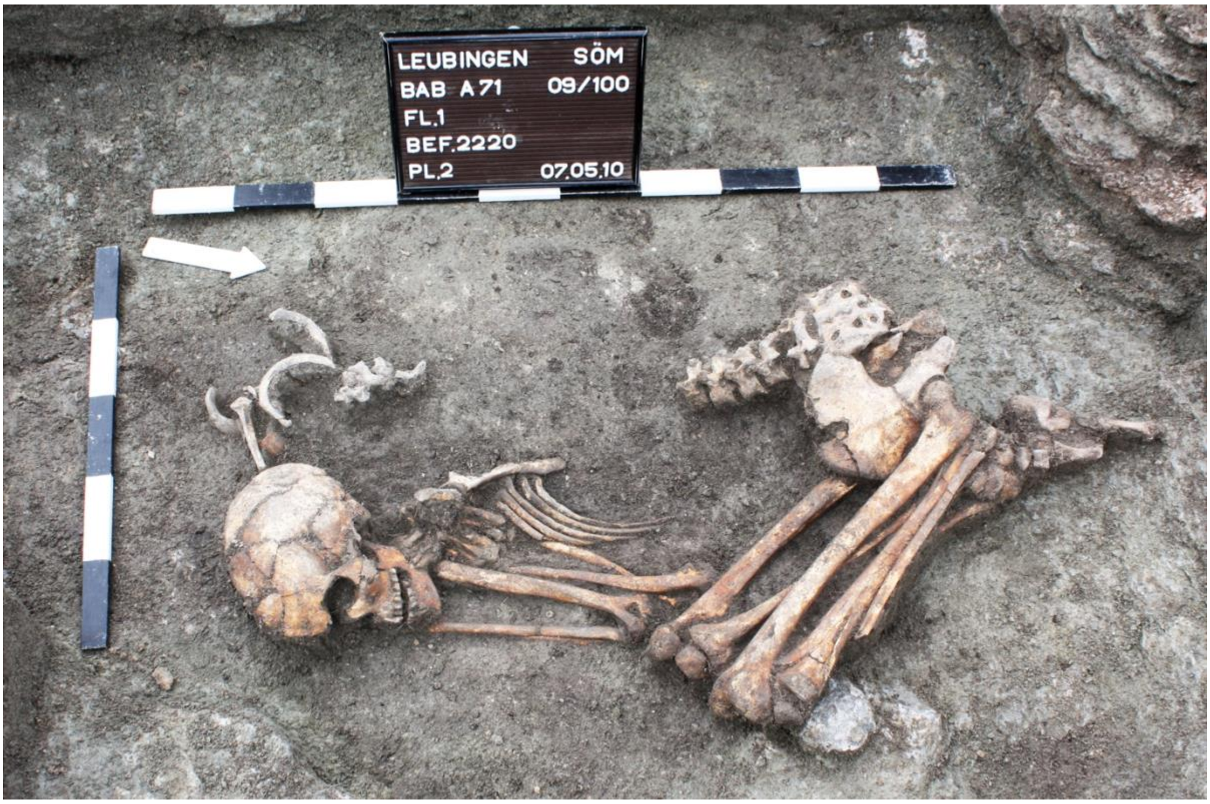

Feature 2220

Feature 2221

09/100-1809, LEU046

Grave:

Flat grave, on the south-eastern edge of the main burial ground, ca. 0.90m south of feature 2220.

Oval grave, 0.84 x 0.60m in size, preserved up to 0.38m into the ground.

Human remains:

Infans I, anthropological sex undetermined, genetically female, 4-5 years old. Oriented south-south-east - north-north-west, crouched on the right side with the head towards south-south-

east, facing east. Remains of the skull, rib fragments, right humerus, hip bones, tarsalia and parts of the legs that were drawn extremely towards the chest.

<u>Grave goods:</u>

None.

<u>Preservation:</u>

Brittle bone substance.

<u>Dating:</u>

Únětice culture.

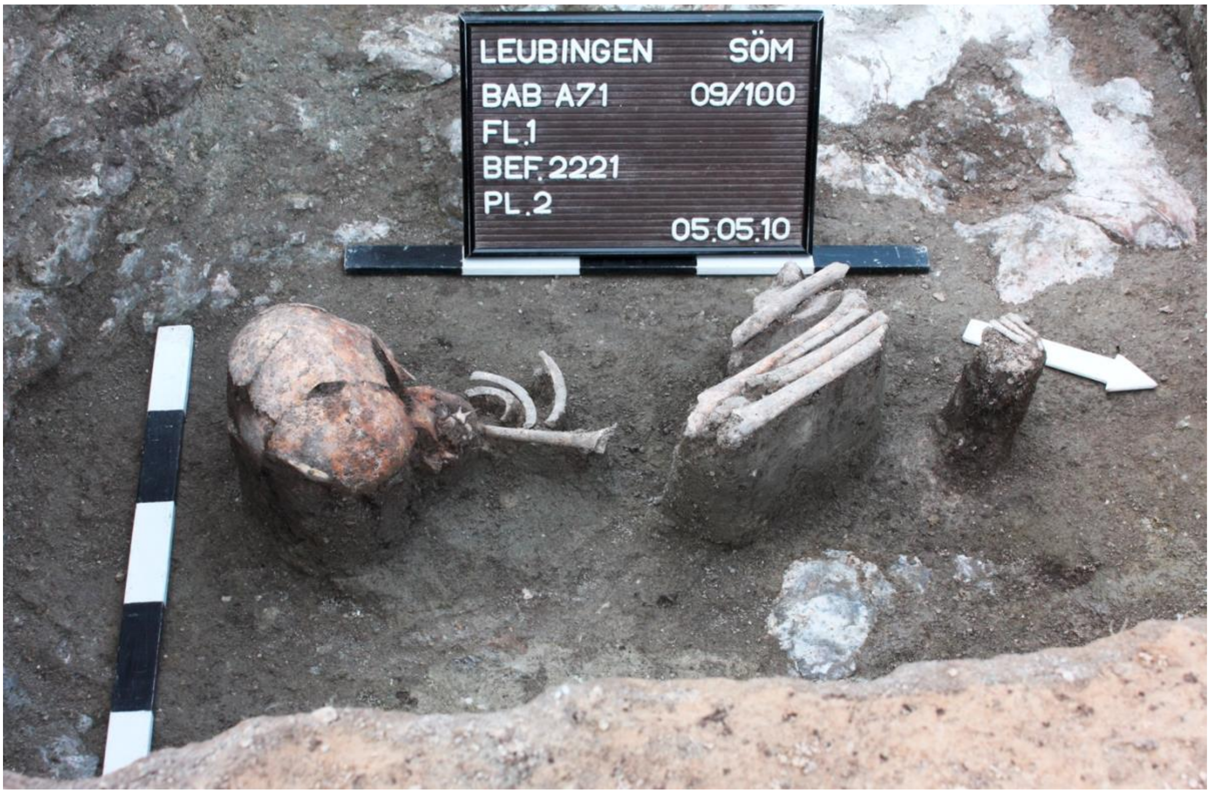

Feature 2221

Feature 2222

09/100-1810, LEU017

<u>Grave:</u>

Flat grave, ca 2.00m away from feature 2221.

Irregular, 1.65 x 1.55m large grave, probably disturbed by a pit in the eastern part. Preserved up to 0.52m into the ground.

<u>Human remains:</u>

Adult individual, anthropologically and genetically male, 26-30 years old, body height 1.68m ± 4cm. Oriented south-south-east - north-north-west, crouched on right side, previously cranium towards south-south-east facing east. The head of the left humerus was destroyed by krotovina, the whole upper arm is moved towards east. The lower left arm was originally

located at a 60° angle in front of the thorax with the hand in front of the face. The upper right arm stretched out under the thorax; the lower right arm laid on top of the right femur towards the pubic bone. Both legs are drawn extremely towards the thorax, the body seems laced. Heavy animal activity in the area of the spine and shoulder girdle.

Grave goods:

None. Settlement ceramics in the disturbed grave area.

Preservation:

Brittle bone substance, heavily disturbed grave.

Dating:

Únětice culture.

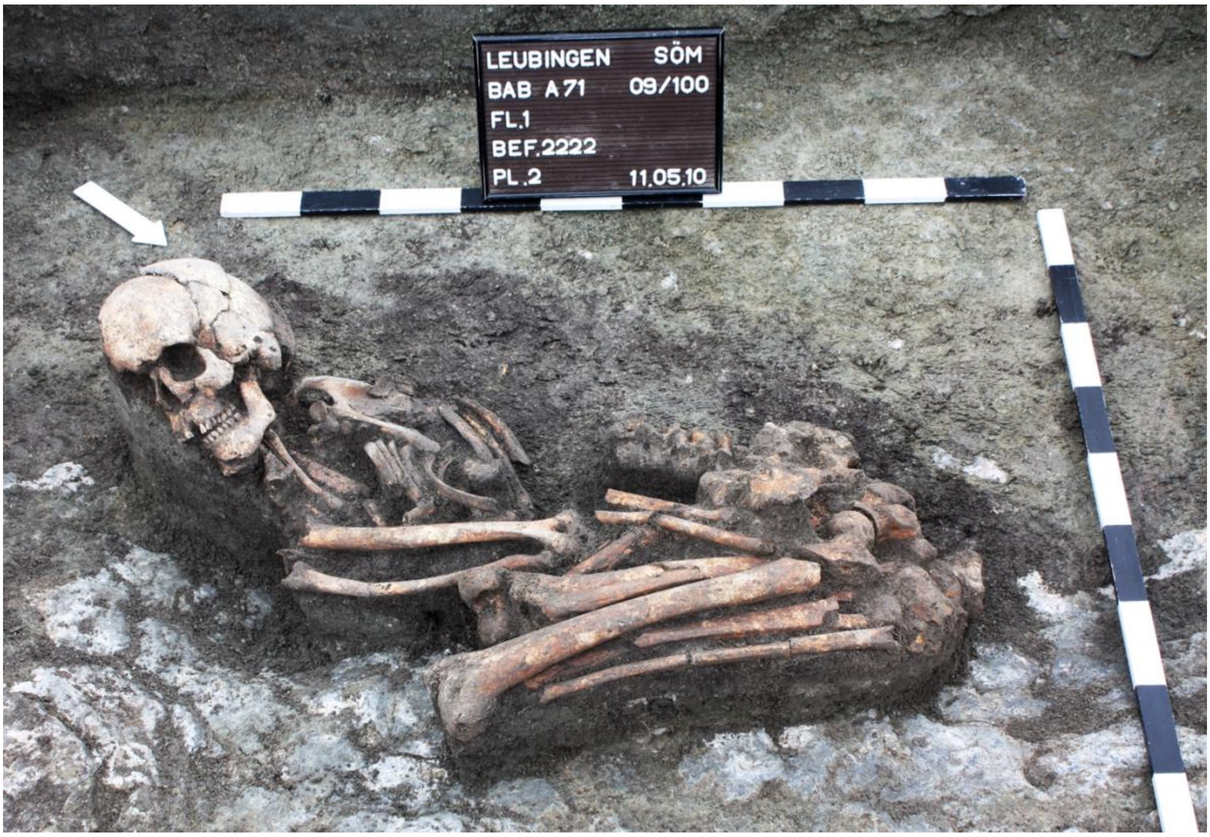

Feature 2222

Feature 2223

09/100-1811, LEU018

Grave:

Flat grave, 0.90m east of feature 2025.

Longitudinal-oval, 1.86 x 1.24m large grave, preserved up to 0.38m into the ground.

Human remains:

Adult individual, anthropologically and genetically female, 30-35 years old, body height 1.50m ± 4cm. Oriented south - north, crouched on the right side with the head towards south, facing

south-east. Crossed arms; the upper right arm west - east oriented, the lower right points north. Radius and Ulna laid between the angled upper and lower arm above the thorax/along the left half of the thorax. The lower left arm points towards an upward facing cup with two perforated T-shaped handles below the rim. The left upper and lower arm laid parallel on the chest. Parts of the hand touched the upper right arm. Both legs are drawn extremely towards the crossed arms/the chest. The right elbow joint touched the right knee. Both heel bones laid underneath the hip and touched the sacrum. The skeleton seems laced.

<u>Grave goods:</u>

Beaker, Evidence of meat offering (scapula of a sheep/goat).

<u>Preservation:</u>

Good preservation.

<u>Dating:</u>

Únětice culture.

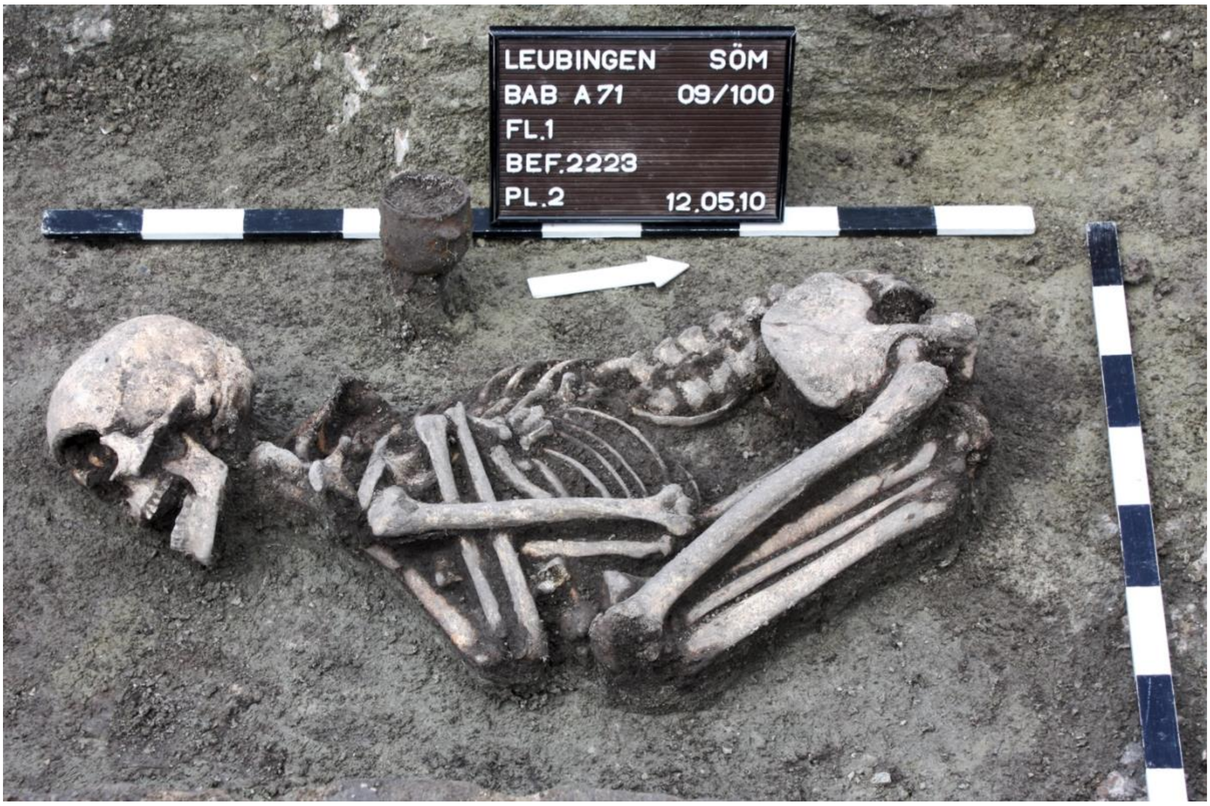

Feature 2223

Feature 2224

09/100-1812, LEU057

<u>Grave:</u>

Flat grave, on the southern border of the main burial ground, ca. 2.50m south-east of feature 2022 and 1.80m south of feature 2023.

Longitudinal-oval grave, 1.50 x 0.75m in size, preserved up to 0.23m into the ground.

Human remains:

Mature individual, anthropologically and genetically female, around 44 years old, body height 1.54m ± 4cm. Oriented south-south-east - north-north-west, crouched on right side with the head towards south-south-eas, facing east. In the area of the spine, thorax and the clavicles heavily disturbed by krotivina. Preserved were parts of the long bones, the cervical vertebrae, the rib bones and the sternum. The legs were extremely drawn towards the hip. In front of the frontal bone was an upright standing shattered cup (vessel 1). North of it, in front of the open mouth, was an upright standing cup with handles. Remains of a bronze ring were next to the left ear. North-north-east of the cup with handles were remains of a meat offering (scapula of sheep or goat).

Grave goods:

Two cups, simple wire ring (copper), scapula (sheep or goat).

Preservation:

Brittle bone substance, heavily disturbed grave.

Dating:

Únětice culture. MAMS 43539: 3679±21 BP, 2141-1976 cal BC.

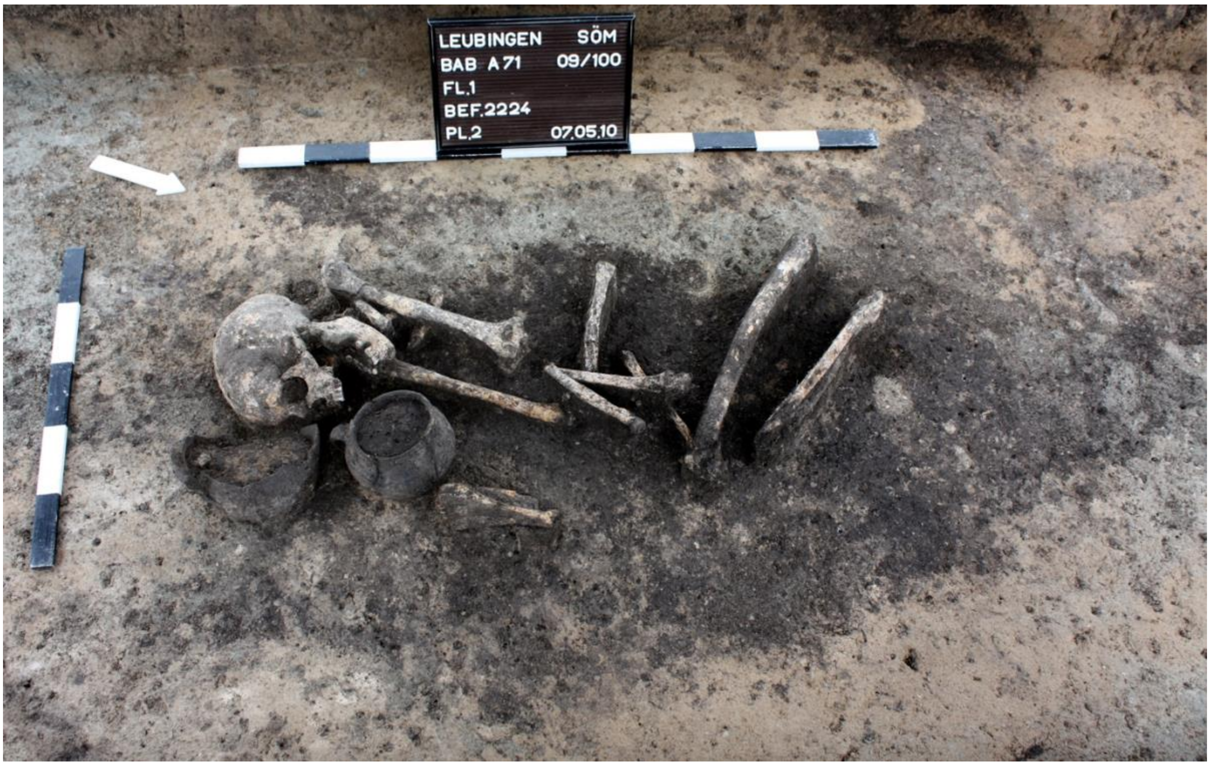

Feature 2224

Feature 2237

09/100-1813, LEU040

Grave:

Flat grave, ca. 1.60m north-north-east of feature 2240.

Irregular oval, 1.00 x 0.63m in size, preserved up to 0.27m into the ground.

Human remains:

Infans I, anthropological sex undetermined, genetically male individual, 2-3 years old. Oriented south-south-east - north-north-west, crouched on the right side with the head towards south-south-east, facing east. Remains of the skull with the lower jaw, rib fragments, parts of the scapula, finger bones and leg bones. Below the lower jaw were shards of a small cup.

Grave goods:

Beaker (or flagon).

Preservation:

Very brittle bone substance. Heavily disturbed grave.

Dating:

Únětice culture.

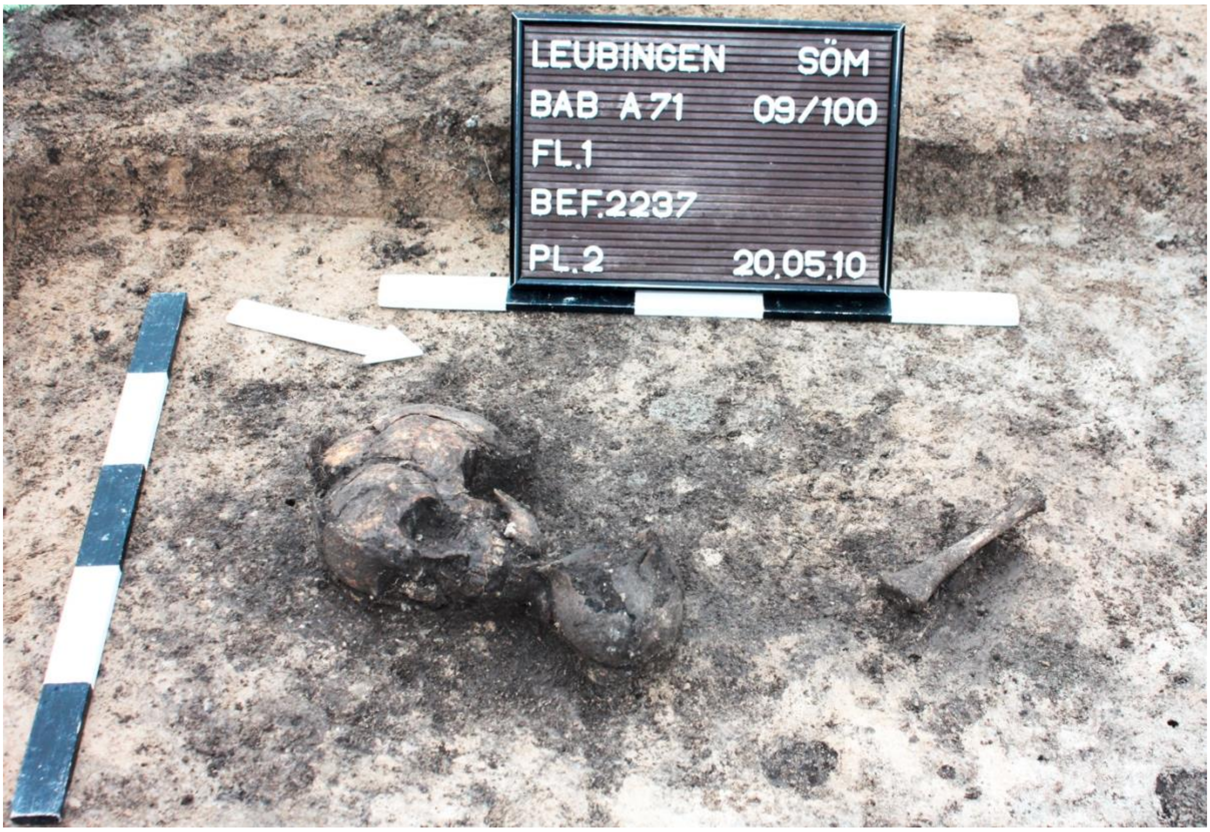

Feature 2237

Feature 2242

09/100-1815, LEU058

Grave:

Flat grave, ca. 1.60m west of feature 2240.

Irregular-oval grave, 2.02 x 1.24m in size, preserved up to 0.50m into the ground.

Human remains:

Adult individual, anthropologically and genetically female, 30-40 years old. Oriented south-south-east - north-north-west, crouched on the right side with the head towards south-south-east, facing east. The upper left arm was moved from its original position towards the lower left arm, underneath the lower right arm. The upper end of the lower left arm and the left hand laid underneath/between the right elbow and the chest. The lower right arm points towards the left shoulder joint, parallel to the upper left arm. Therefore, the arms were originally crossed. Single finger bones of the right laid at/on top the left shoulder. The upper left leg laid at a 90° angle to the ilium on top of the right leg. The upper right leg was drawn closer to the chest; the end of the right calf and the tarsalia laid underneath the left ankle. The heel bones point towards the pelvic girdle. A large, handleless, bulbous cup with a narrow diameter of the opening (vessel 1) was standing upright south of the head. East of this was another upright standing large pot (vessel 2) with a broad, inferior handle. Directly in front of the face was a cup in an inclined position with two perforated T-shaped inferior handles. East of the cervical spine was a flat, round bone disc with cross shaped scoring lines around the perforation. On top of the left temple was a relatively large, double winded spiral ring made out of bronze. Remains of a second ring laid near the right temple.

Grave goods:

One beaker, one flagon, one pot, two spiral wire rings (copper), one perforated bone disc.

Preservation:

Well preserved.

Dating:

Únětice culture. MAMS 34796: 3717±22 BP, 2199-2033 cal BC.

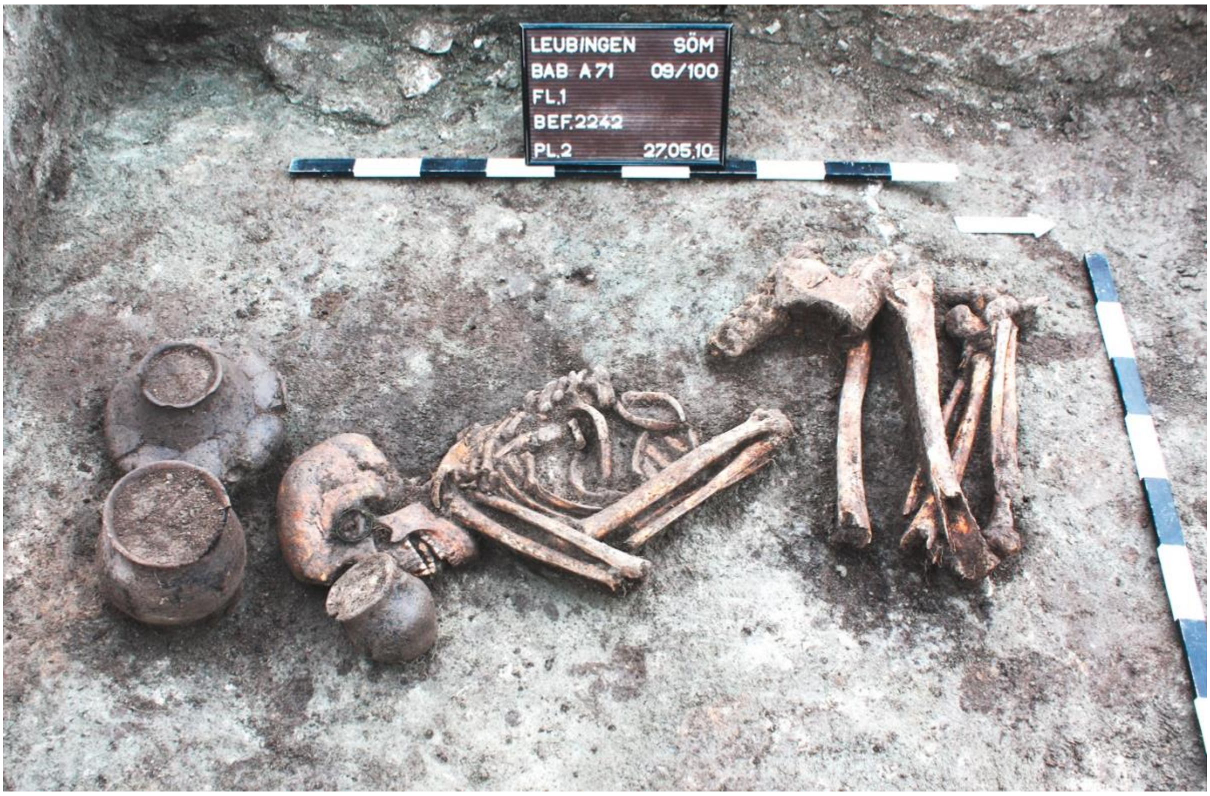

Feature 2242

Feature 2243

09/100-1816, LEU031

<u>Grave:</u>

Flat grave, 0.85m north of feature 2244.

Irregular, oval grave, 1.75 x 1.50m in size, preserved up until 0.48m into the ground.

<u>Human remains:</u>

Adult individual, anthropologically and genetically male individual, 35-39 years old, body height 1.71m ± 4cm. Oriented south - north, crouched on the right side with the towards south, facing upwards/north. The upper body and the hip laid horizontally in a supine position, the legs on the right side. The upper left arm was parallel to the thorax, the lower left arm and the right hand are pointing east at a 90° angle. The right arm was stretched along the right body side, the hand was bent towards the upper right leg at the height of the right knee. The left femur laid at a 90° angle to the ilium behind the right leg, the left fibula and the left clavula were removed from the anatomical bond. The left food is missing.

<u>Grave goods:</u>

None.

<u>Preservation:</u>

Bones well preserved.

<u>Dating:</u>

Únětice culture. MAMS 43529: 3664±21 BP, 2136-1956 cal BC.

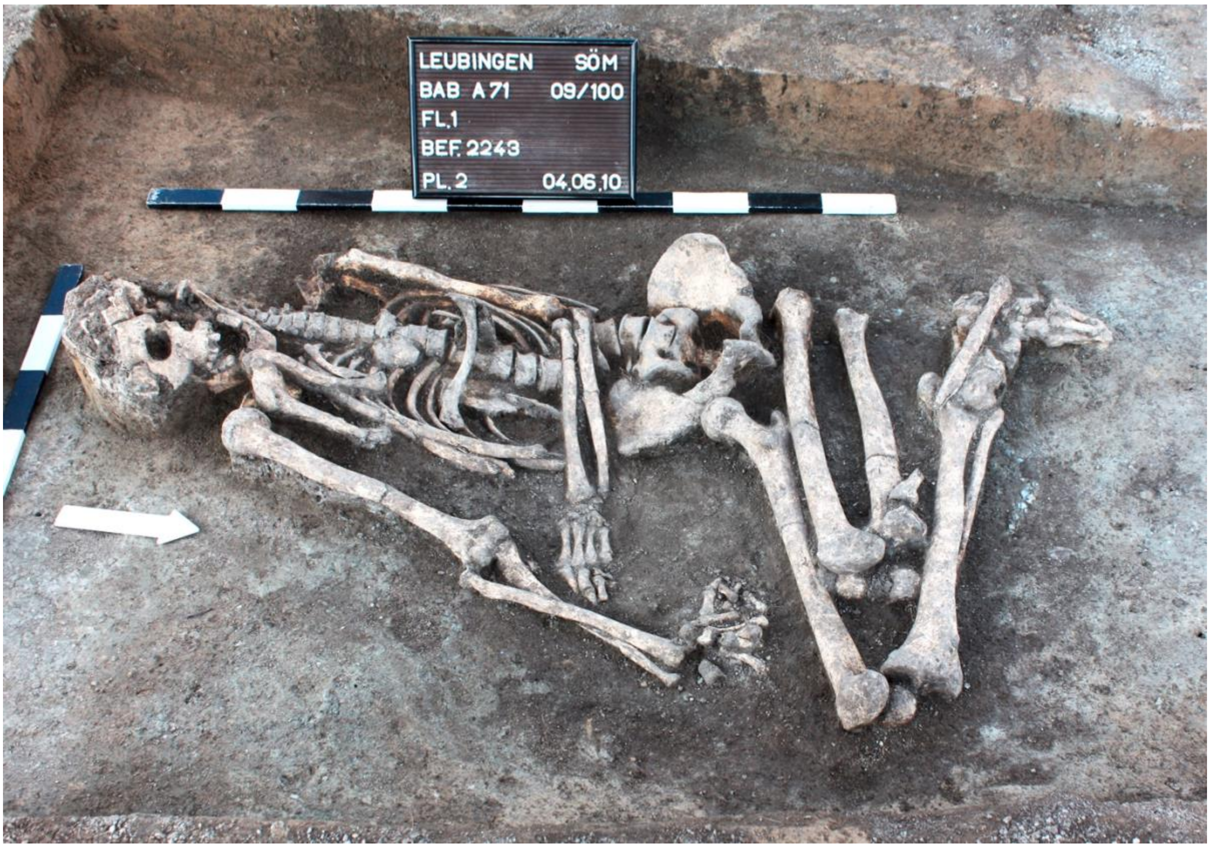

Feature 2243

Feature 2244

09/100-1817, LEU033

Grave:

Flat grave, 0.85m south of feature 2243.

Longitudinal-oval grave, 1.10 x 0.74m in size, preserved up to 0.10m into the ground.

Human remains:

Early adult individual, anthropologically and genetically male, 17-18 years old. Oriented south - north, crouched on the right side with the head towards south, facing north-east. The upper right arm laid underneath the chest; the lower right arm was parallel along the rib cage. Remains of the metacarpal bones were located at the upper right arm and in the area of the skull. Both femurs were drawn extremely towards the chest. The whole left side of the body, the spine and the pelvic girdle were heavily disturbed.

Grave goods:

None.

Preservation:

Poorly preserved bone substance.

Dating:

Únětice culture.

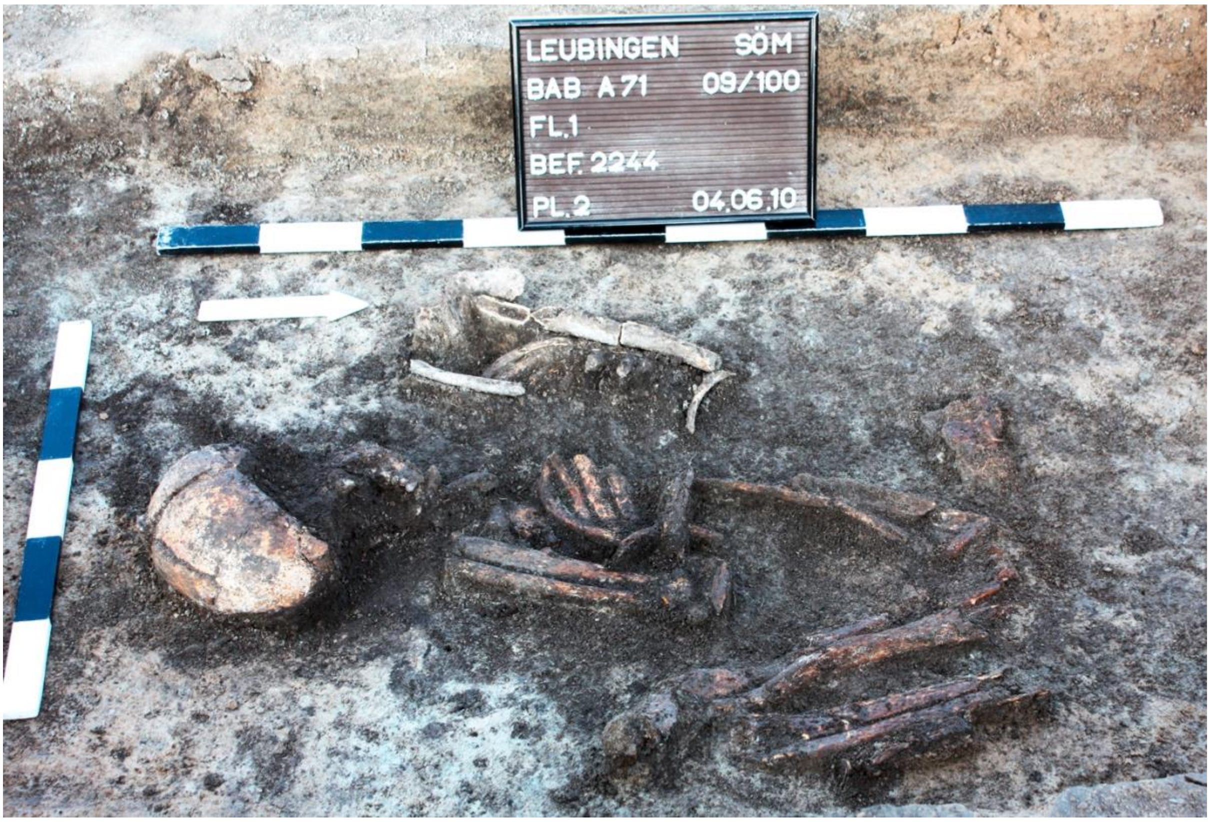

Feature 2244

Feature 2247

09/100-1818, LEU034

<u>Grave:</u>

Flat grave, 0.80m north of feature 2026.

Irregular and rectangular grave, 2.40 x 1m in size, preserved up to 0.34m into the ground.

<u>Human remains:</u>

Adult individual, anthropologically and genetically male, 27-30 years old, body height 1.72m ± 4cm. Oriented south - north, crouched on the right side with the head towards south, facing east. Supine position, the lower right arm points south towards the upper right arm at a 90° angle (almost parallel to the pelvic girdle). The upper left arm laid on the chest, the lower left arm points east-south-east at a right angle. The left femur laid at an 80° angle to the ilium above the right leg. The right femur is drawn closer to the chest. Parts of the left food are displaced; metacarpal bones are missing. In front of the face and the joint of the right humerus was a fragmented shard of a large storage vessel with the opening to the south (vessel 1). South of the opening, directly above the Skull was an upright standing cup with handles (vessel 1).

<u>Grave goods:</u>

Cup, fragmented storage vessel, filling with ceramic shard and animal bones.

Preservation:

Brittle bone substance.

Dating:

Únětice culture.

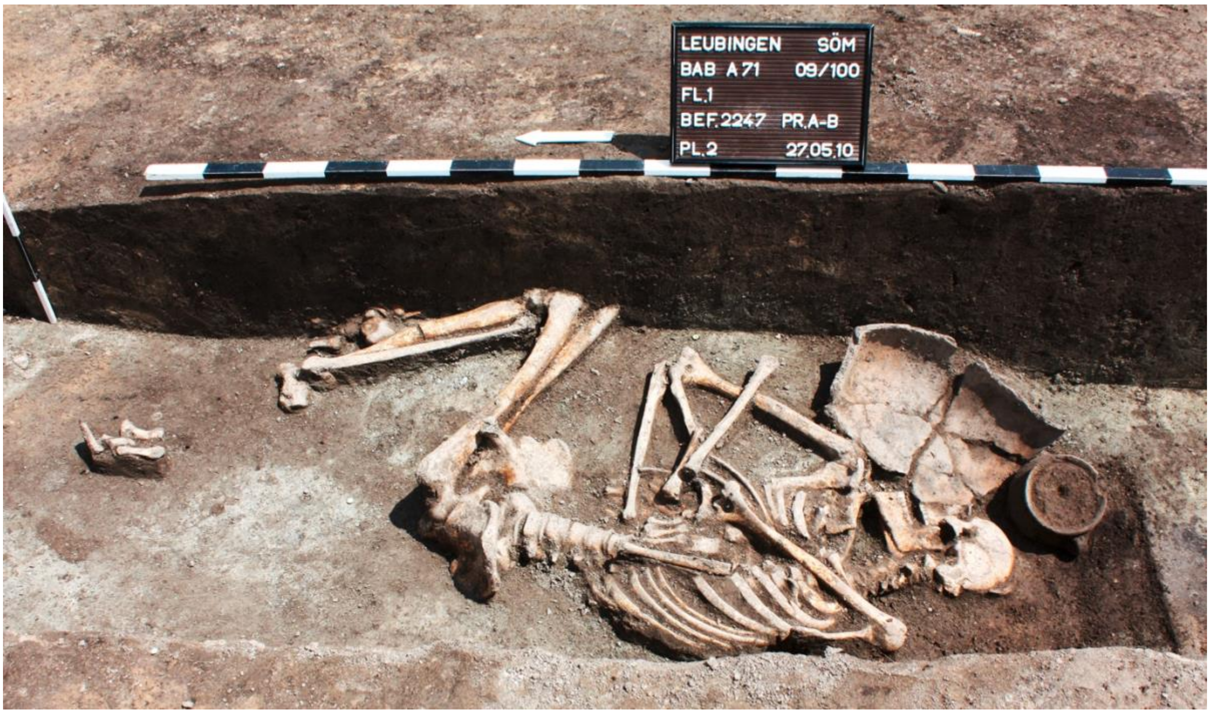

Feature 2247

Feature 2565

09/100-1822, LEU041

Grave:

Flat grave, on the eastern edge of the main burial field, ca 0.70m east of feature 2219. Longitudinal-oval, 1.35 x 0.70m in size, preserved up to 0.50m into the ground.

Human remains:

Juvenile individual, anthropologically possibly female, genetically male, 15-17 years old. Oriented south - north, crouched on the right side with the head towards south, facing east. The lower jaw was removed from the anatomical context and laid slightly higher between the skull and the left scapula. The upper left arm was on the ribs, the lower left arm was underneath the lower right arm pointing towards south-east at a 60° angle. The upper right arm is not visible in planum 2. Radius and Ulna stretched towards the left shoulder. Single finger bones of the right hand laid along the upper left arm and the left clavicle. The upper left leg laid above the right leg at a 90° angle to the ilium. The upper right leg was drawn more towards the chest. The right elbow joint laid underneath the upper right leg.

Grave goods:

Rubbing stone (ground stone).

Preservation:

Good bone preservation, disturbed by bioturbation.

Dating:

Únětice culture. MAMS 43532: 3716±21 BP, 2198-2034 cal BC.

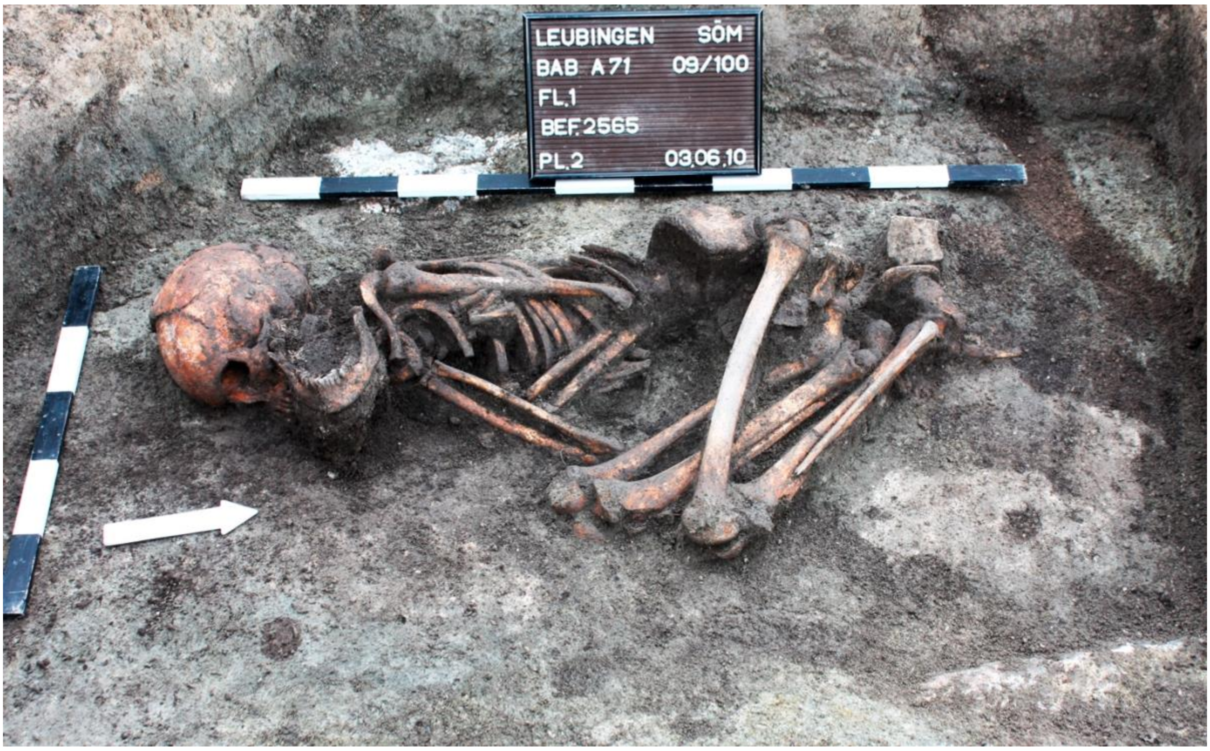

Feature 2565

Feature 5281

09/100-3785, LEU037

Grave:

Pithos burial, ca. 106m east of the main burial ground.

Remains of two coarse ceramic vessels in the black soil (irregular oval, 0.62 x 0.50m) surrounded by at least three rocks (north-north-west - south-south-east). Shards of the side parts; vessel 1 with opening towards south-east, inside overlapping vessel 2 with the opening towards north-west. Both vessel remains contained bones from a child burial.

Human remain:

Neonatus, anthropological sex undetermined, genetically female individual, 0-6 months old.

Grave goods:

Typical early Bronze Age ceramics (remains of two storage vessels as container for the individual), no other grave goods.

Preservation:

Poorly preserved bone substance. Disturbed grave.

Dating:

Únětice culture.

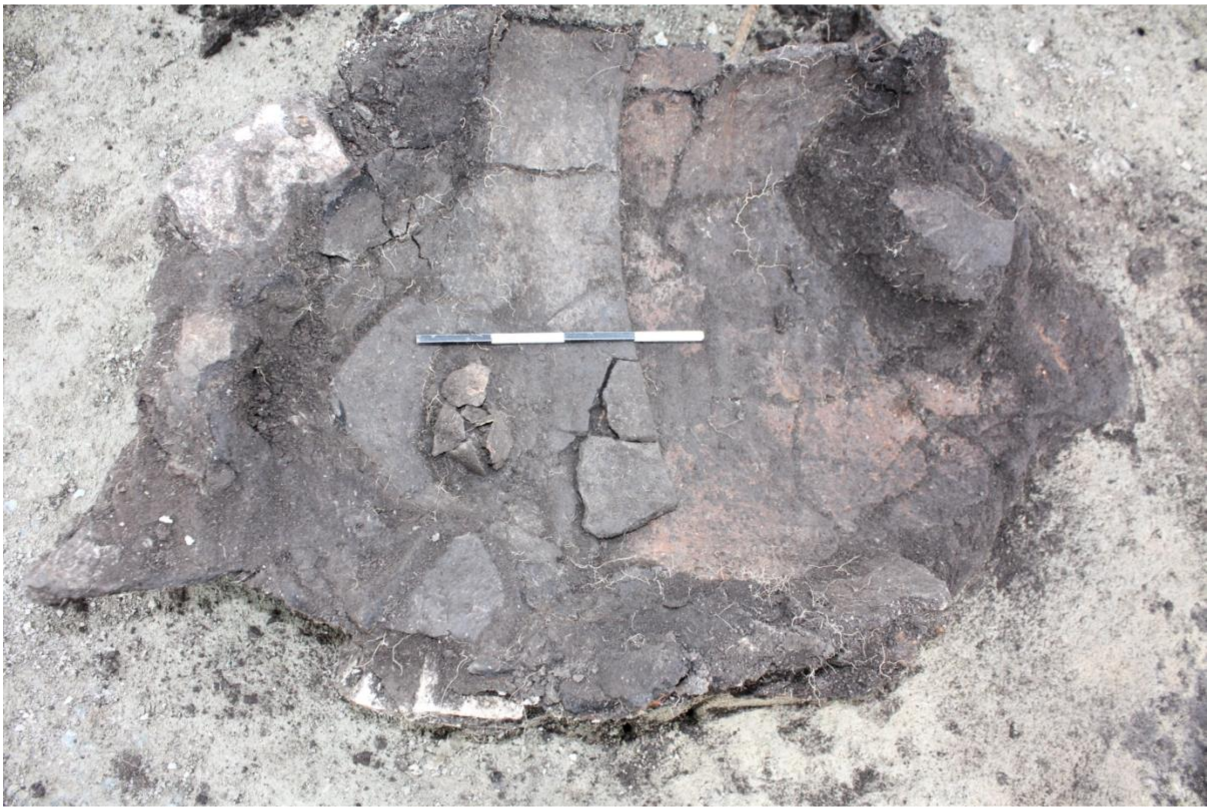

Feature 5281

1.2. Leubingen II

Feature 81

10/111-22, LEU059

Grave:

Disturbed Pithos burial(?) in the settlement.

Almost circular feature with a diameter of about 2m. The depth of the pit was 0.45m with a slightly irregular bottom. Within the pit human bones were found as well as fragments of a storage vessel around the individual. The skull laid close to surrounding ceramic shards, so that it can be assumed it was originally placed within the vessel.

Human remains:

The skeleton was severely disturbed and not in anatomically correct articulation. Based on the human bones found lying to the north of the almost complete skull, it can be assumed that the individual was lying with his head to the south. The human bones are the remains of a 0-7 year-old child (infans I), anthropological sex undetermined, genetically female.

Grave goods:
Storage vessel (fragments), flake (made of flint), shell fragment.
Preservation:
Poorly preserved, disturbed by later EBA(?) pit.
Dating:
Únětice culture. MAMS 34797: 3591±22 BP, 2024-1887 cal BC.

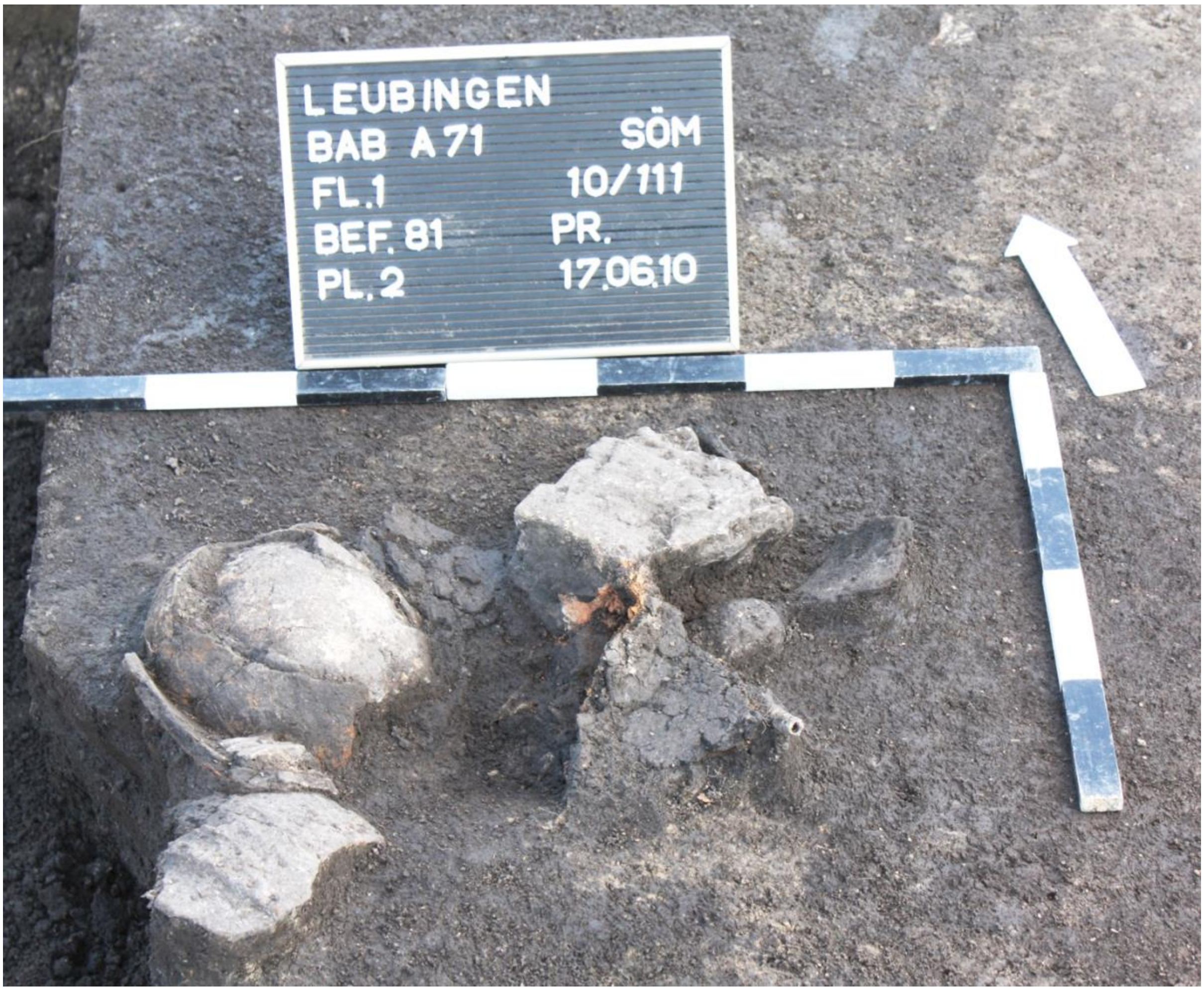

Feature 81

Feature 142

10/111-37, LEU044

Grave:

Completely preserved burial in the form of a regular single grave. The grave is a large oval-shaped pit with diameters of 2.12m (east - west) and 1.65m (north - south) and a maximum depth of 0.30m. Besides human remains, pottery fragments, which probably represent the remains of a vessel, and animal bones in the pit filling were found.

Human remains:

The individual was lying in an east-south-east - west-north-west direction with the head to the south. It was buried in a crouched position on the right side, facing east. The person was about 20 - 40 years old (adult), anthropological sex undetermined, genetically female.

<u>Grave goods:</u>

Fragments of a single(?) vessel, shell fragment.

<u>Preservation:</u>

Good bone preservation.

<u>Dating:</u>

Únětice culture.

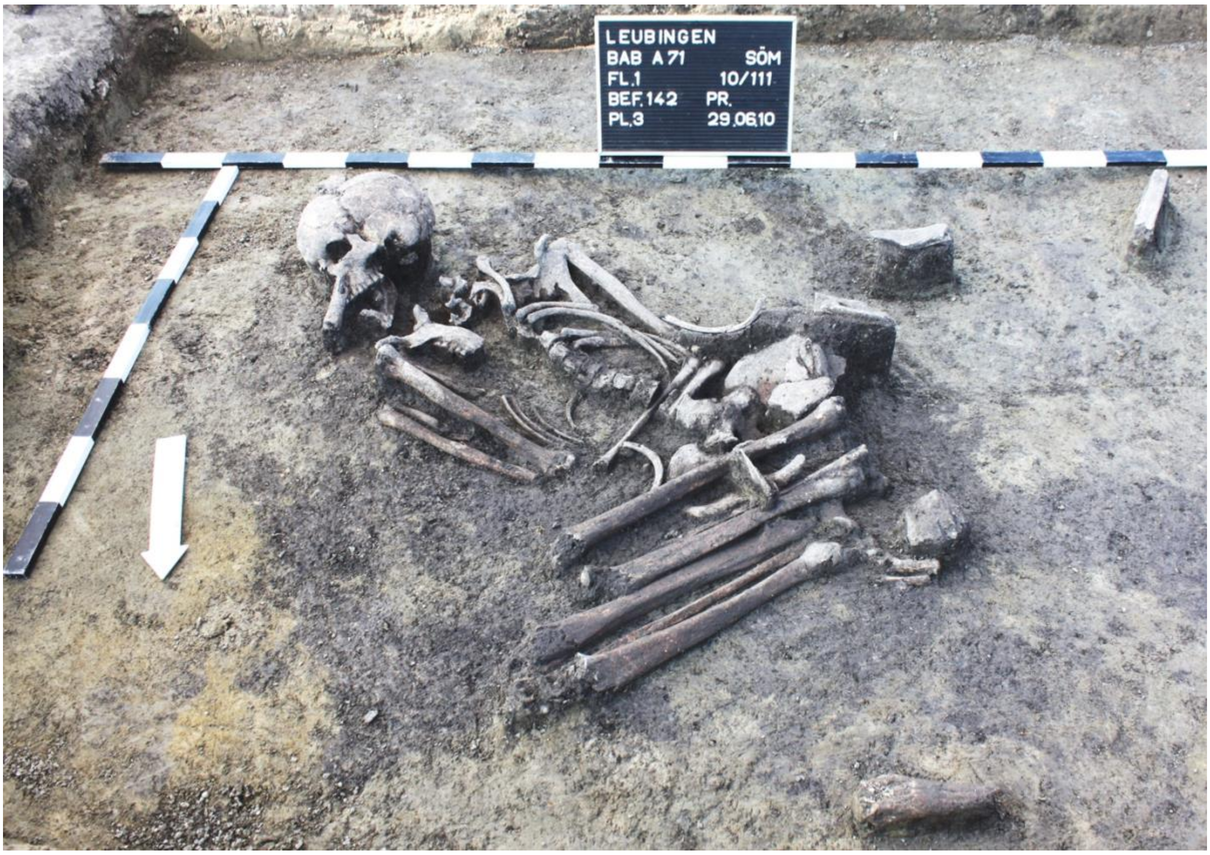

Feature 142

Feature 201

10/111-57, Ind. 1, LEU060 and

10/111-56, Ind. 2, LEU039

<u>Grave:</u>

The oval feature 201 had a north - south extension of about 1.05m and a west - east extension of 0.88m. The maximum depth was 0.42m. In the center of the pit, the skeleton of a 20-40 year old human (adult) (I1) and sparse remains of a second individual were recovered (I2). The anthropological sex of I1 and I2 is not determined, I1 is genetically male and I2 is genetically female.

Human remains:
The adult individual laid in a strongly crouched position on the right side, oriented north - south, with the head in the south, facing east. The burial site was undisturbed, so the adult skeleton was almost completely preserved. In addition, several pottery shards and animal bones were found in the filling of the pit. Two shell fragments are assigned as grave goods.
Grave goods:
Two shell fragments.
Preservation:
Good bone preservation.
Dating:
Únětice culture. MAMS 34798: 3603±22 BP, 2027-1893 cal BC (ind. 201-1).

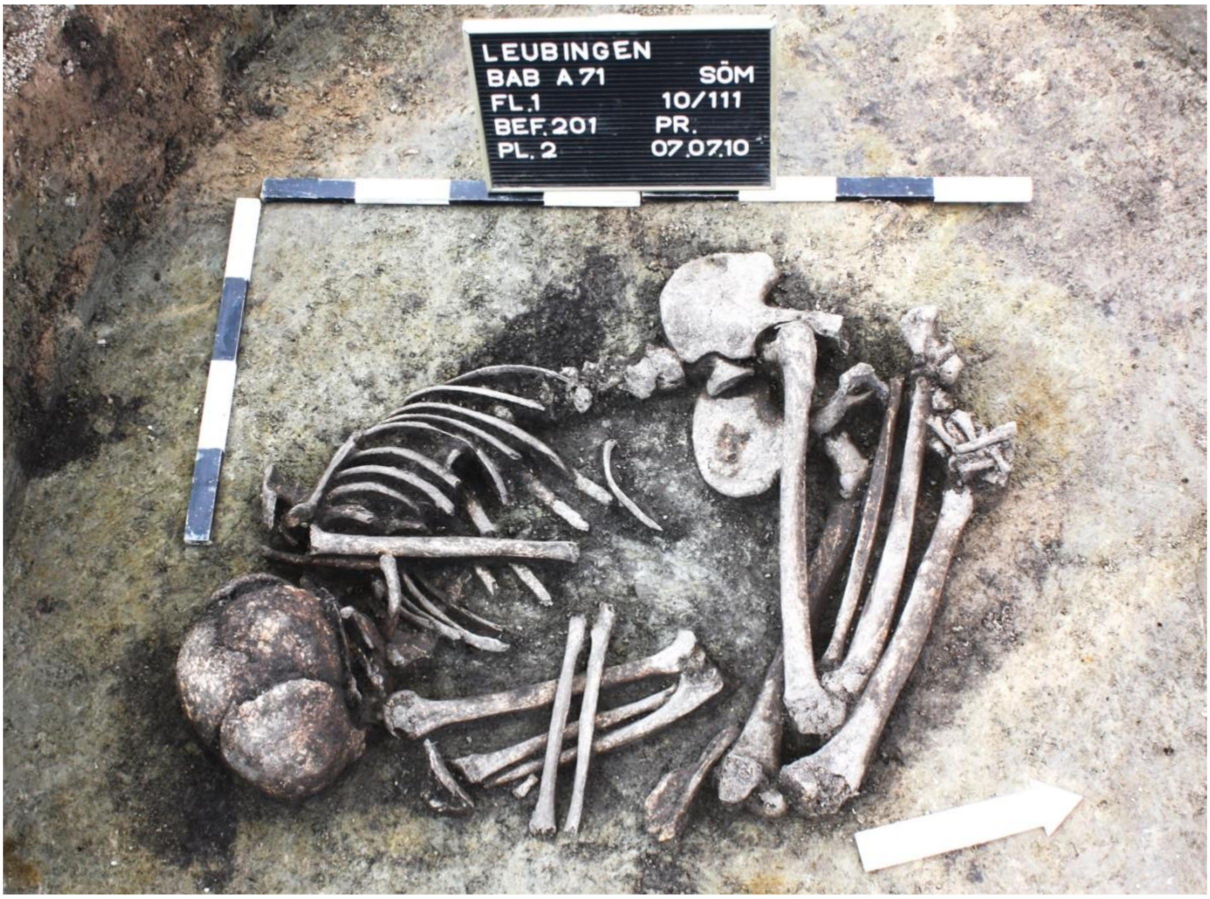

Feature 201

Feature 203
10/111-62, LEU061
Grave:
The feature is irregularly bounded due to disturbances by small mammals. The pit was slightly oval in outline and north-north-west - south-south-east orientated with a length of 0.9m and a width of 0.8m. The maximum depth of the feature below the Ap-horizon was 0.2m. In the

western part of the burial pit the remains of a human individual were found. A small stone axe was found below the chest area of the skeleton. A rubbing stone was placed at the feet and in the filling of the pit contained shards.

Human remains:

The individual (infans I-II) was buried in a crouched position and in a south- north direction, the head towards south, facing probably east. From the skull only parts of the calotte and the jaw were preserved. Besides a few other remains, only parts of the leg and arm bones could be found. The anthropological sex is undetermined, the genetic sex is male.

Grave goods:

axe (ground stone), rubbing stone (ground stone).

Preservation:

Moderate bone preservation.

Dating:

Únětice culture.

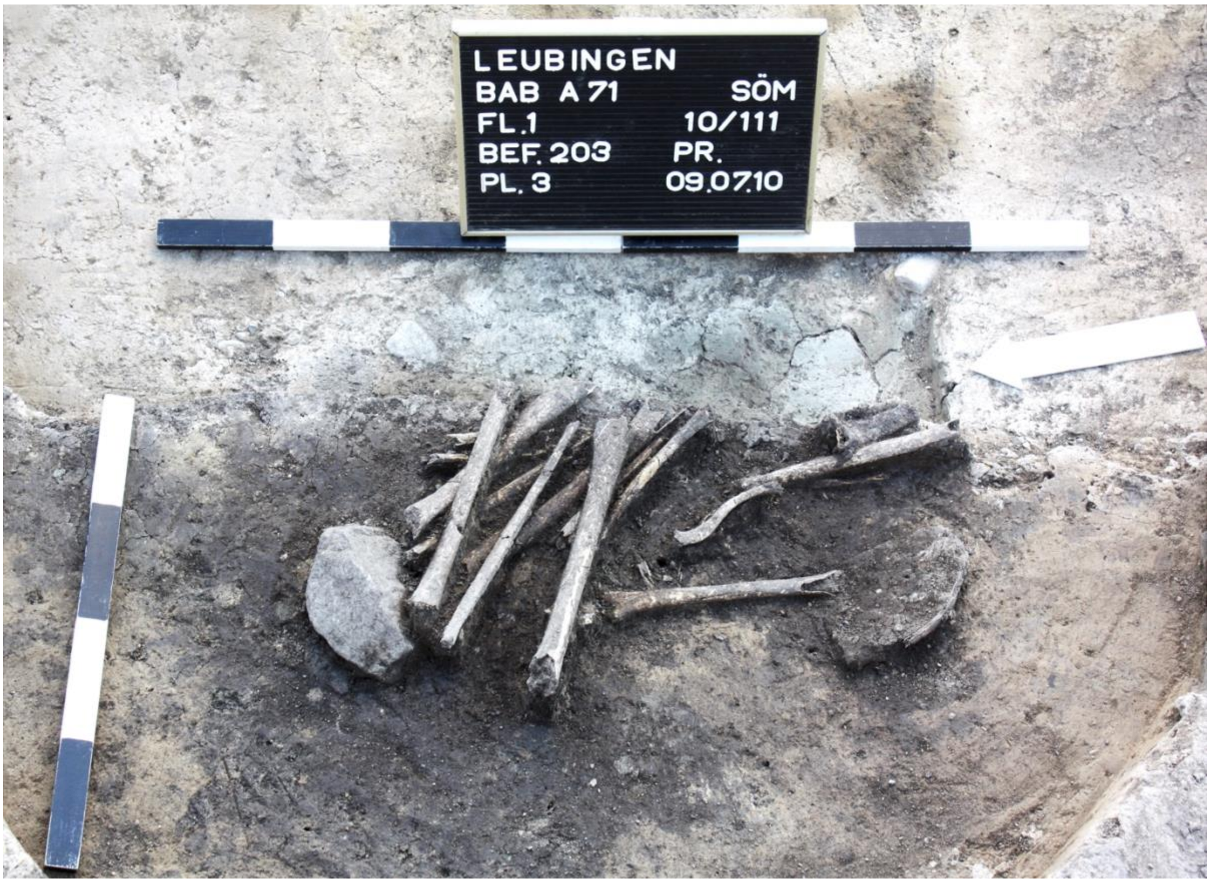

Feature 203

2. Statistical analysis for grave goods

For both of the response variables “Number of grave goods” and “Number of different types of grave goods”, we used Poisson regression models to account for the fact that these are both count variables. We began by considering if a Poisson model fit the outcome variables by looking at the sample mean to sample standard deviation ratio, which in both cases seemed reasonable (0.9324 and 0.9882, respectively). We then fit the regression models with the most complex interaction model possible, and used stepwise AIC to select the best nested model. For “Number of grave goods" we calculated Cragg and Uhler’s pseudo r^2 using the *pscl* package[3]. When comparing the final model to the null model, we calculated a p-value via a likelihood ratio test using the *lmtest* package[4].

We performed Correspondence Analysis (CA) using the *FactoMineR* package[5]. We fit the analysis using the counts of different grave good types, including only individuals with a total count greater than zero. We first included the number of stone tools in the CA; however, this variable completely dominated the first principal dimension, and we soon identified that only one individual (2565/LEU041) was buried with a stone tool, and that this individual had no other grave goods. Hence, we removed this outlier and re-ran the CA (Fig. 4b).

We included supplementary variables in the analysis which did not contribute to the calculation of the principal dimensions, but are instead “projected” onto them to explore relationships between the individuals, grave good types, and our supplementary variables. These variables included age at death, genetic sex, isotopic cluster, and family (pedigree or non-pedigree). We assessed that two principal dimensions sufficiently explained the variability as every principal dimension after dimension two singularly explains less than $\frac{100}{6}\%$ of the overall variability (where six is the number of variables used to calculate the principal dimensions[6]).

3. Statistical analyses of kinship

To analyze the effect of age group and sex on the pairwise mismatch rate (PMR) we performed a two-way analysis of variance (ANOVA) on the ranks[7] of the PMR values calculated using BREADR. We used rank regression as the raw PMR values are trimodal, and violate the assumption of normality. We then performed Tukey's Honest Significant Difference (HSD) test as a post hoc analysis to test the differences between the groups.

Although the effects of age and sex were deemed to be significant, the PMR values are calculated on the same individuals many times (in pairs), and this could violate the assumption of independence. Hence, we used an assumption-free randomisation test to independently assess whether the effect of sex was statistically significant. To do this we randomly permuted the sex and age group for all individuals 100,000 times, and performed the ANOVA and HSD on the permuted data, and recorded the estimated mean rank difference between the XX/XX and XY/XY groups. We calculated an empirical p-value of 9.9×10^{-6}, which corresponds to the maximum possible value indicating that no mean rank difference was as extreme as we observed in the true data (see Supplementary Fig. S5b). The empirical p-value was calculated as

$$p_e = \frac{r+1}{N+1}$$

where r is the number of mean rank differences from the label-permuted data that were further from the mean than the observed value and N is the number of permutations.

The Wilcoxon Rank Sum tests of the mean PMR value per individual were performed as per Rivollat *et al.* 2023[50].

4. Population genetic analyses

To assess the genetic profiles and to explore a possible subtle genetic variation between Leubingen I and II with respect to published EBA individuals from Central Germany, we used Principal Component Analysis (PCA). The individuals from Leubingen I and II fall within the variation of published EBA groups associated with the Únětice culture in Central Germany[8] as well as from Bohemia and the Czech Republic[9] (Supplementary Table F, Supplementary Fig. S6a). Hence, we combined Leubingen I and II as a single genetic group, called LEU_EBA. Interestingly, published EBA individuals from the Lech Valley in Southern Germany, associated with the Straubing cultural group, fall lower on PC2[10]. We formally tested for possible differences in the ancestry in EBA groups from Central and Southern Germany and Bohemia using f_4-statistics of the form f_4(EBA 1, EBA 2; *test*, Mbuti) where '*test*' are different CW and BB associated groups from Central Europe.

LEU_EBA yielded non-significant values (|Z|≤3) only when compared to Bohemia_Unetice_Classical, whereas all other tested groups yielded significant positive values for BB (Lech_EBA) or BB-, and CW-associated groups (Bohemia_Unetice_preClassical, Germany_EBA_Unetice) (Supplementary Fig. S7a, Supplementary Data G). The EBA individuals from the Lech Valley yielded significant positive values (|Z|≥3) for BB-associated groups when compared to LEU_EBA, Bohemia_Untice_preClassical and Bohemia_Unetice_Classical (Supplementary Fig. S7a, b), which is consistent with their lower position on PC2 and also indicated an increase in BB-related ancestry in southern German EBA groups, rather than an increase in CW-related ancestry in the population of Leubingen (Extended Data Fig. S6a). By contrast, published Bohemian and Central German EBA individuals associated with the Únětice culture show evidence of increased genetic influence from CW groups, indicated by significantly negative values (|Z|≥3) for Bohemia Únětice and significantly positive values (|Z|≥3) for Germany_Unetice_EBA when compared to Germany_Lech_EBA (Supplementary Fig. S7b, c).

The difference in genetic ancestry between Central Germany/Bohemia EBA and Southern Germany EBA could be confirmed using distal qpAdm modeling[11,12] where, for LEU_EBA, Germany_EBA_Unetice, Bohemia_Unetice_preClassical and Bohemia_Unetice_Classical, where estimated similar ancestry proportions of Turkey_N, CHG, WHG and EHG ancestry (~30% + ~26% + ~14% + ~30%) whereas Germany_Lech_EBA differed by carrying a higher amount of Turkey_N (~46%), but a lower amount of CHG (~20%) and EHG (~20%) ancestry (Supplementary Fig. S6b, Supplementary Table H).

Based on these results and the subtle differences between the EBA groups highlighted by f_4-statistics, we modeled LEU_EBA and relevant contemporaneous EBA groups as local and non-local BB- and CW-associated groups. The modeling of LEU_EBA results in well-fit models with Central German BBs (Germany_BellBeaker_CG) (~79%) and either the local Central German CW substrate (~19%, p=0.05) or the non-local Bohemia_CW_Early (~21%, p=0.37) or Bohemia_CW_Late (~23%, p=0.18) (Supplementary Fig. S6c, Supplementary Table I). Bohemia_Unetice_Classical could be modeled the same as LEU_EBA as Germany_BellBeaker_CG and Bohemia_CW_Early (~80% + ~20%, p=0.7). The preceding Bohemia_Unetice_preClassical could be modeled as Bohemia_BB_Late (~52%), Bohemia_CW_Early (~36%) and Latvia_BA (~12%), consistent with Papac *et al.* 2021[10]. The published Germany_EBA_Unetice could be modeled as 100% Germany_BellBeaker_CG (Supplementary Fig. S6c, Supplementary Table I). These differences in genetic ancestry between the EBA groups are further supported by the fact that we could not model LEU_EBA as one of the contemporaneous EBA groups ($p \leq 0.05$) (Supplementary Table J).

	Total	# of							
		ceramics	metal	silex	stone tools	Meat offering	Bony artifacts	shells	others
**Subadult**	1.39	0.56	0	0	0.11	0.11	0.11	0	0.11
**Adult**	1.69	0.46	0.15	0.12	0	0.12	0.08	0.04	0.12
**XX**	2.07	0.6	0.27	0.13	0	0.13	0.07	0	0
**XY**	1.275	0.4	0	0.05	0.05	0.1	0.1	0.05	0.2
**Pedigree A**	2.6	0.8	0.2	0	0	0.2	0.2	0	0
**Pedigree B**	1.79	0.71	0	0.14	0	0	0.14	0.14	0.29
**Pedigree C**	1.67	0.56	0.11	0	0	0.22	0	0	0.22
**Pedigree D**	0	0	0	0	0	0.	0	0	0
**Non-pedigree**	1.33	0.25	0.17	0.17	0.08	0.08	0.08	0	0

Supplementary Table S1 | Number of grave goods per category. Number of different grave goods of each category per variable. Given is the mean of each grave good type per variable. This table corresponds to the results from the CA where we find no correlation between grave goods and demographic variable (see main text and Supplementary Information Part 2).

6. Supplementary Figures S1 – S7

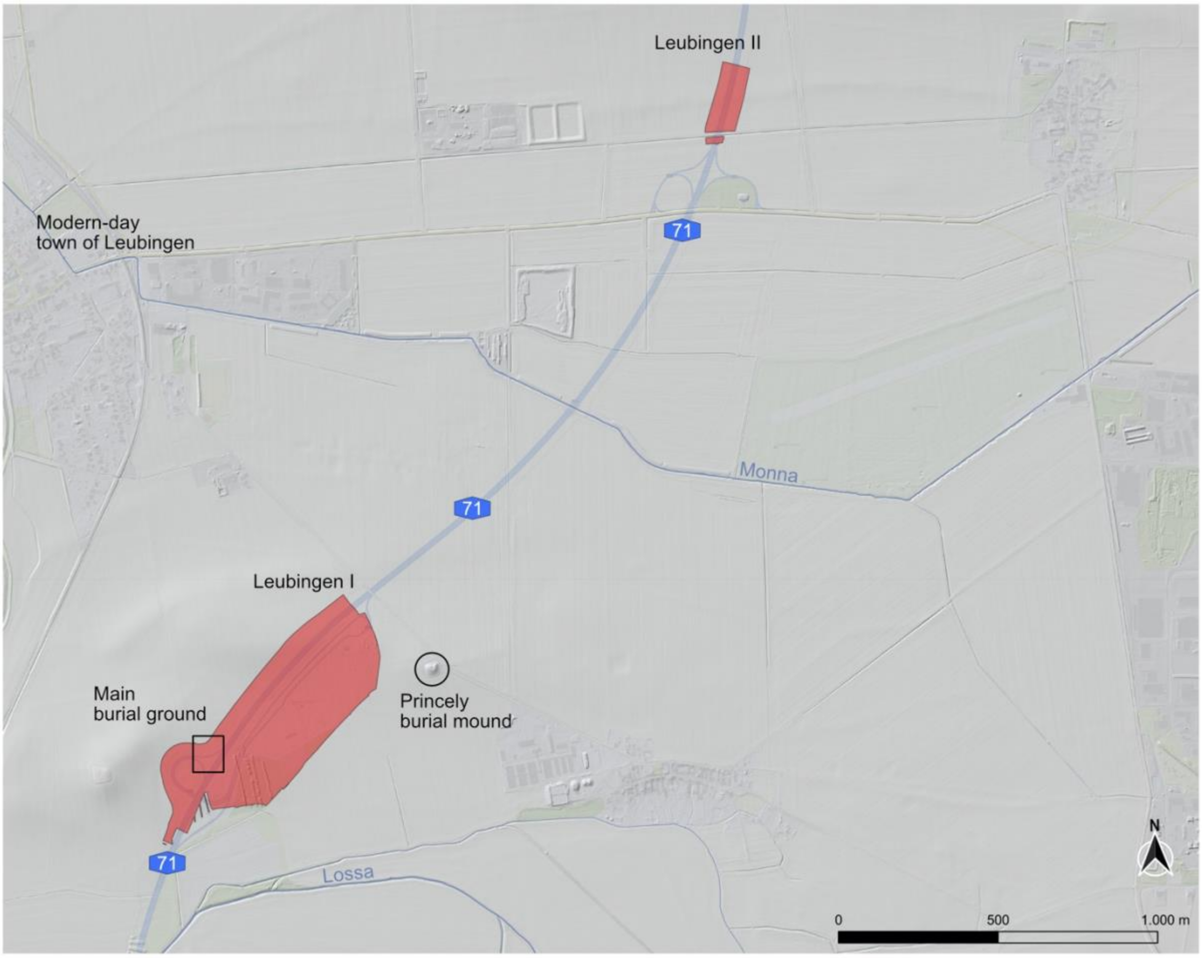

Supplementary Fig. S1 | Overview over the location of the burial ground of Leubingen. Marked in red are the excavation sites of Leubingen I and II. The black square indicated the main burial ground as shown in Fig. 2b in the main article. The black circle marks the princely burial mound. Map was created using the free and Open Source QGIS 3.22 and the GDI Thuringia (Geodateninfrastruktur, Landesamt für Bodenmanagement und Geoinformation).

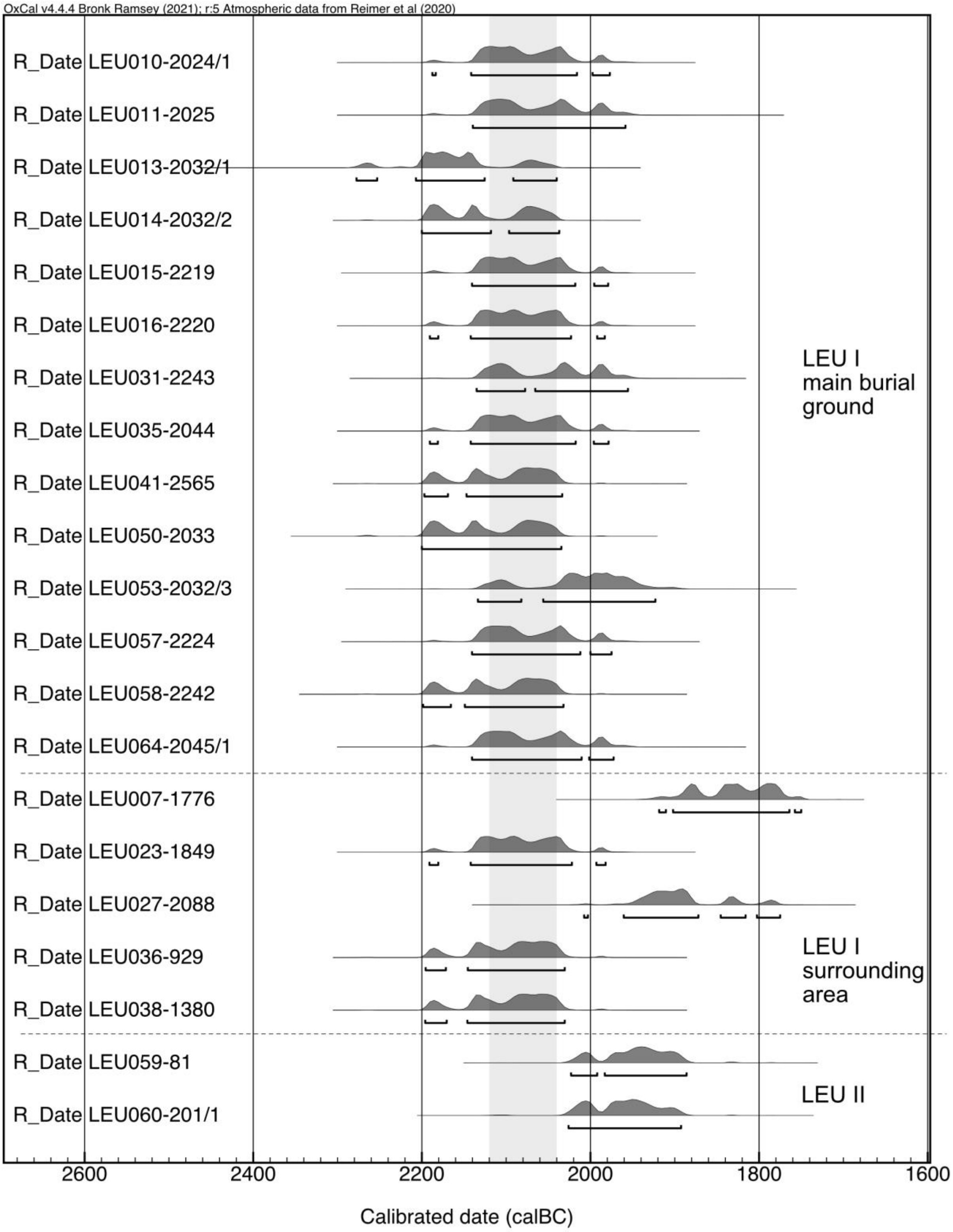

Supplementary Fig. S2 | Calibration results (with OxCal v4.4, shown is the 2σ range) of all ^{14}C-dates dates for Early Bronze Age individuals from Leubingen I and II. Individuals LEU007, LEU023, LEU027, LEU036, and LEU038 are from graves outside the main burial ground of Leubingen I. The gray marks the AK 1b phase (Schwarz 2021, 2022[40]) of the Únětice Culture.

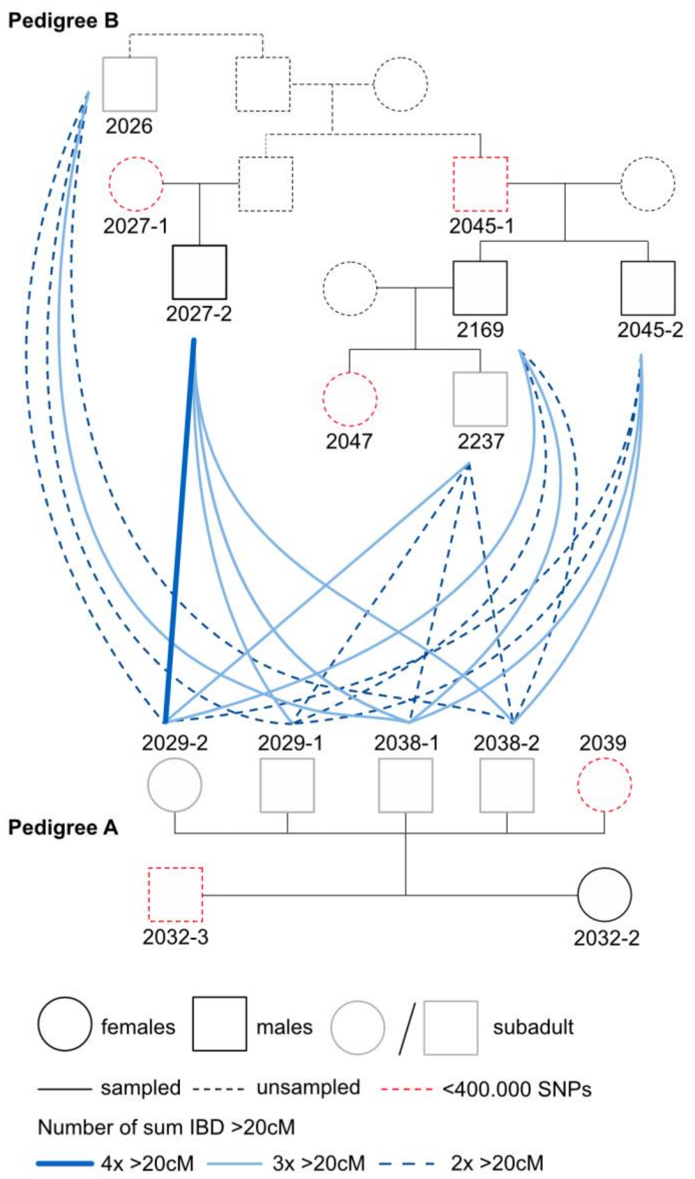

Supplementary Fig. S3 | Distant genetic relationships between pedigree A and B based on IBD sharing. All present individuals with more than 400.000 SNPs of pedigree B share two or more IBD tracks >20cM with all present children of pedigree A.

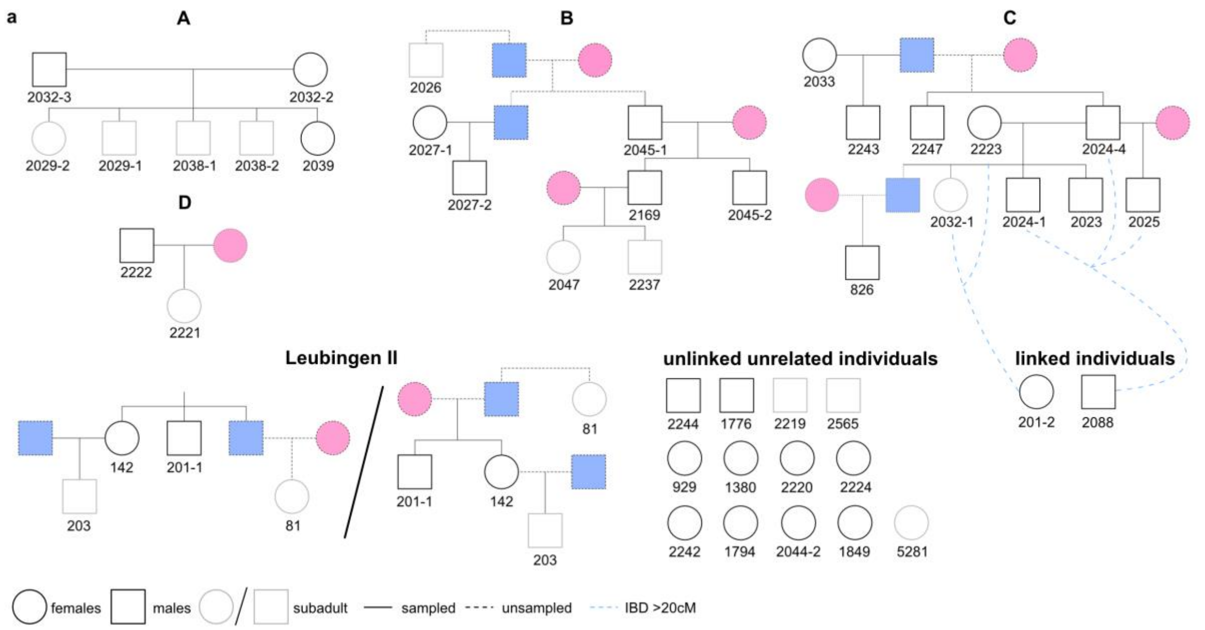

Supplementary Fig. S4 | Missing individuals from Leubingen I and II based on the reconstructed pedigrees. All pedigrees, except pedigree A, show evidence for missing individuals. For pedigree A, B and the pedigree from Leubingen II we find both male and female individuals to be not present at the burial ground but necessary for reconstructing the pedigree. When male individuals are missing from the pedigree they are linked to other individuals from the pedigree, i. e. siblings or parents whereas the missing female individuals are not linked to the pedigree except via their children. An exception is the male individual from the first generation from pedigree A as well as the male individual in the first/second generation in Leubingen II. Blue squares mark missing male individuals and pink circles mark missing female individuals.

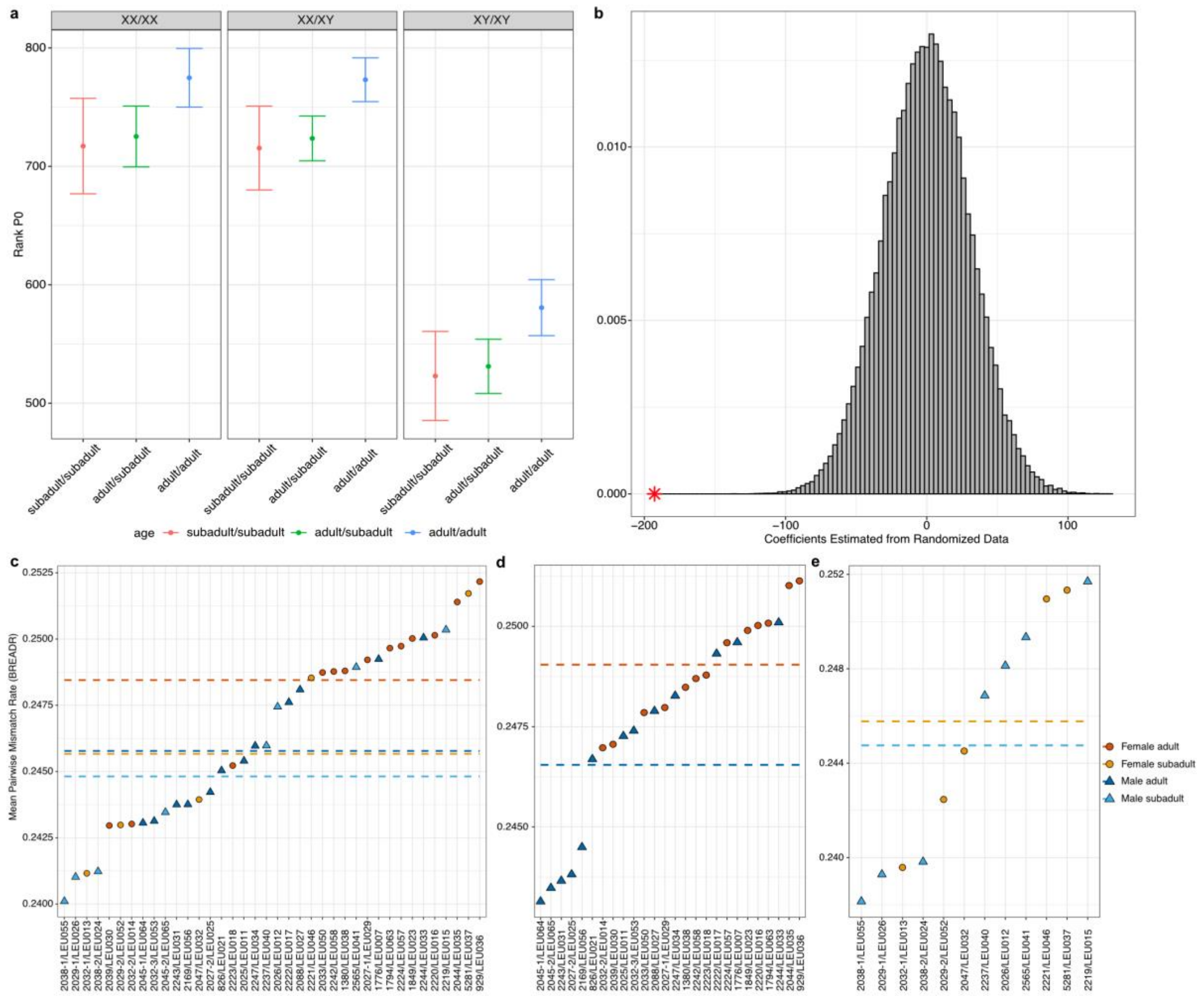

Supplementary Fig. S5. Relatedness of individuals from the burial ground. a, Rank differences between genetic sex and age pairings, estimated using a linear regression of the ranks of the PMR values, indicating that XY/XY have a significantly lower average PMR than XX/XX and XY/XX **b,** A histogram of the mean rank differences between XX/XX and XY/XY PMR values for 100,000 label-permuted data sets. The red star indicates the true observed value. **c,** Mean pairwise mismatch rate for each individual from the burial ground. There is no significant differentiation between the relatedness of males to other individuals than of females (p=0.06727). **d,** Mean pairwise mismatch rate for adult individuals (older than 16 years old). The difference between female and male relatedness is significant (p=0.01404), with females being less related to other individuals than males. **e,** Mean pairwise mismatch rate for subadult individuals (younger than 16 years old). There is no significant difference between the relatedness of subadult females and males (p=0.7551). Shown are the feature numbers and the lab IDs.

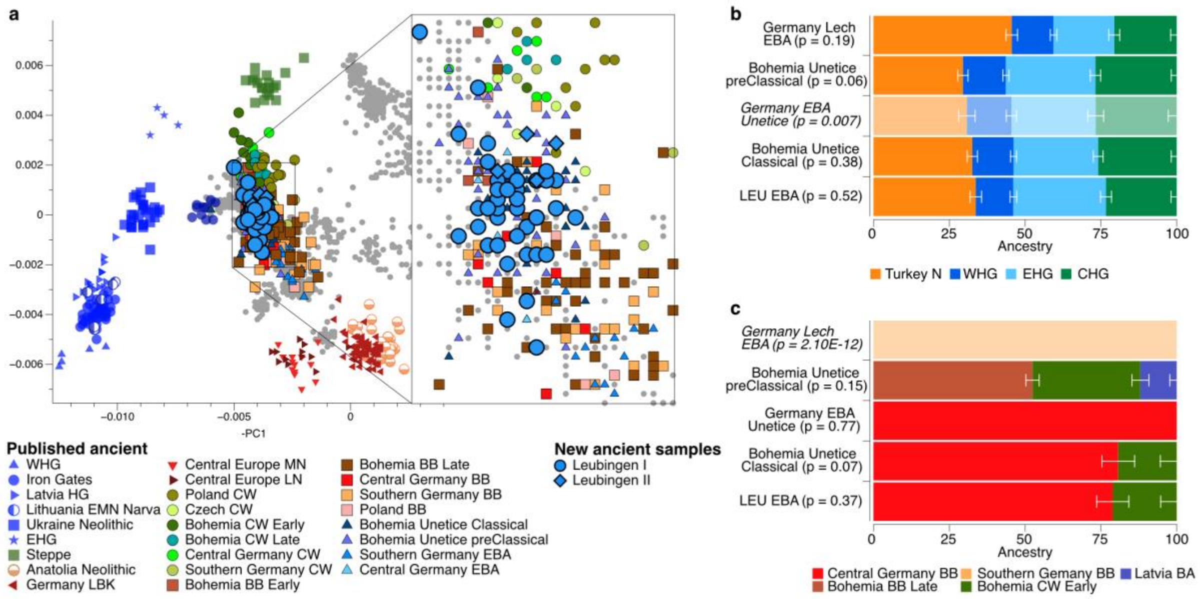

Supplementary Fig. S6 | Principal Component Analysis (PCA) and qpAdm ancestry modeling. a, Newly reported individuals from Leubingen I and II (coloured symbols with thick black outline) and relevant published groups (coloured symbols, no outline/thin black outline) projected onto the West Eurasian genetic variation of 1,324 individuals from 83 populations. **b**, distal qpAdm modeling and **c**, proximal qpAdm modeling for LEU_EBA and relevant contemporaneous groups.

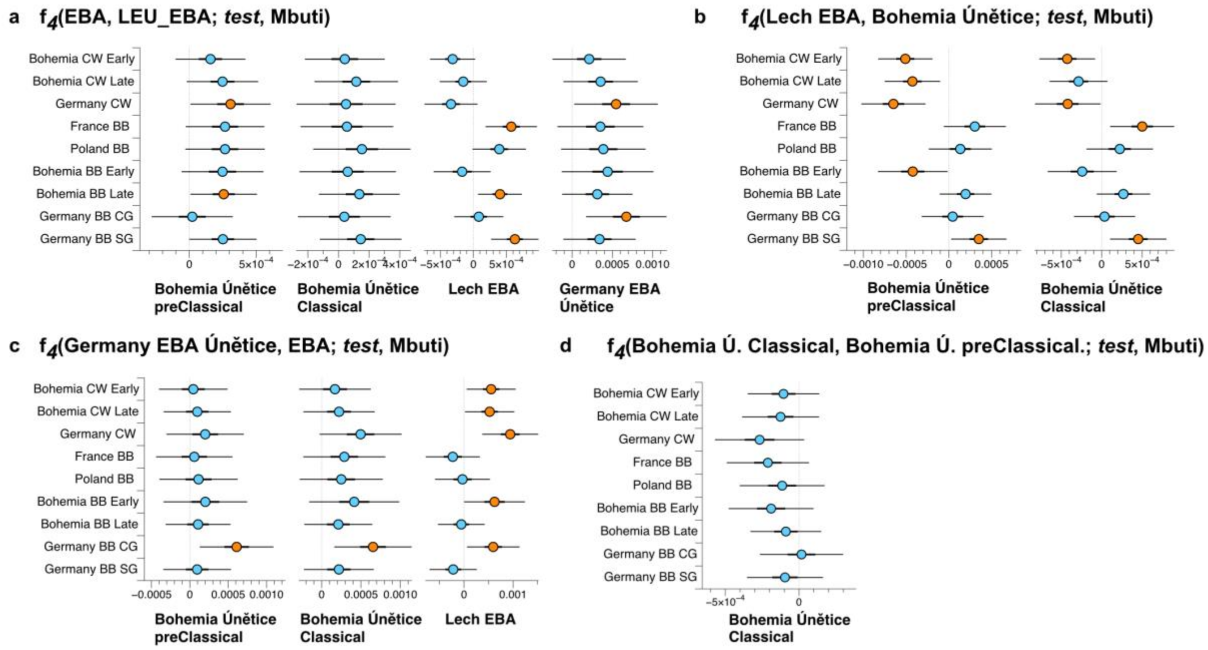

Supplementary Fig. S7 | F_4-Statistics for different relevant EBA groups from Germany and Bohemia. Shared drift between **a,** LEU_EBA **b,** Central German Únětice **c,** Southern German EBA and **d,** Bohemia Únětice and BB- and CW-associated groups with respect to relevant contemporaneous EBA groups.

7. References

1. Ferembach, D. Recomendations for age and sex diagnosis of skeletons. *J. Hum. Evol.* **9**, 517–549 (1980).
2. Rösing, F. W. *et al.* Recommendations for the forensic diagnosis of sex and age from skeletons. *Homo* **58**, 75–89 (2007).
3. Jackman, S. *pscl: Classes and Methods for R Developed in the Political Science Computational Laboratory. United States Studies Centre, University of Sydney. Sydney, New South Wales, Australia*. (2020).
4. Zeileis, A. & Hothorn, T. Diagnostic Checking in Regression Relationships. *R J.* **2**, 7–10 (2002).
5. Lê, S., Josse, J. & Husson, F. FactoMineR: An R Package for Multivariate Analysis. *J. Stat. Softw.* **25**, 1–18 (2008).
6. Bendixen, M. T. Compositional perceptual mapping using chi-squared trees analysis and correspondence analysis. *J. Mark. Manage.* **11**, 571–581 (1995).
7. MacFarland, T. W. & Yates, J. M. Kruskal–Wallis H-Test for Oneway Analysis of Variance (ANOVA) by Ranks. in *Introduction to Nonparametric Statistics for the Biological Sciences Using R* (eds. MacFarland, T. W. & Yates, J. M.) 177–211 (Springer International Publishing, 2016).
8. Mathieson, I. *et al.* Genome-wide patterns of selection in 230 ancient Eurasians. *Nature* **528**, 499–503 (2015).
9. Papac, L. *et al.* Dynamic changes in genomic and social structures in third millennium BCE central Europe. *Sci Adv* **7**, (2021).
10. Mittnik, A. *et al.* Kinship-based social inequality in Bronze Age Europe. *Science* **366**, 731–734 (2019).
11. Haak, W. *et al.* Massive migration from the steppe was a source for Indo-European languages in Europe. *Nature* **522**, 207–211 (2015).
12. Mittnik, A., Wang, C.-C., Svoboda, J. & Krause, J. A Molecular Approach to the Sexing

of the Triple Burial at the Upper Paleolithic Site of Dolní Věstonice. *PLoS One* **11**, e0163019 (2016).

13. Knipper, C. *et al.* Female exogamy and gene pool diversification at the transition from the Final Neolithic to the Early Bronze Age in central Europe. *Proc. Natl. Acad. Sci. U. S. A.* **114**, 10083–10088 (2017).

40. Schwarz, R. Zur Chronologie der Aunjetitzer Kultur in Mitteldeutschland, Typentafeln zur Chronologie in Mitteldeutschland - Die Aunjetitzer Kultur auf Grundlage der Grab- und Siedlungskeramik. *Studia Hercynia* **23**, 36–53 (2019 2021).

50. Rivollat, M. *et al.* Extensive pedigrees reveal the social organization of a Neolithic community. *Nature* 1–7 (2023).
